# Evolving global trends in PCOS burden: a three-decade analysis (1990–2021) with projections to 2036 among adolescents and young adults

**DOI:** 10.3389/fendo.2025.1569694

**Published:** 2025-05-12

**Authors:** Jiameng Wang, Boyu Wang, Chengjia Li, Tianwei Meng, Changxing Liu, Jia Chen, Ying Guo

**Affiliations:** ^1^ Graduate School of Heilongjiang University of Chinese Medicine, Harbin, China; ^2^ Department of Three Gynecology, First Affiliated Hospital of Heilongjiang University of Chinese Medicine, Harbin, China

**Keywords:** adolescents, young women, global burden, PCOS, trends

## Abstract

**Background:**

Polycystic Ovary Syndrome (PCOS) is a common endocrine disorder affecting adolescent and young adult females, yet global data on its burden and trends remains limited.

**Methods:**

We analyzed data from the Global Burden of Disease Study 2021 for females aged 10–24 years in 204 countries (1990-2021). Metrics included cases, age-standardized incidence (ASIR), prevalence (ASPR), disability-adjusted life years (ASDR), and average annual percentage changes (AAPCs). Future trends (2022-2036) and disease reduction gaps were assessed.

**Results:**

From 1990 to 2021, global PCOS cases increased by 56% (incidence), 59% (prevalence), and 58% (DALYs). ASIR rose from 49.45 to 63.26 per 100,000, with an AAPC of 0.8. Southeast Asia, East Asia, and Oceania had the fastest growth, while high-SDI regions bore the highest burden. Girls aged 10–14 showed the steepest age-specific increase. Nationally, the largest increases occurred in Equatorial Guinea, Maldives, and Myanmar, while Italy saw a decline. Forecasts through 2036 indicate continued increases in ASIR (+8.32%), ASPR (+10.87%), and ASDR (+10.39%). Frontier analysis highlighted unachieved reduction potential, especially in high-SDI countries.

**Conclusions:**

PCOS burden among adolescents and young adults has significantly risen globally with disparities by region, SDI, and age, warranting urgent and equitable public health strategies.

## 1 Introduction

Polycystic ovary syndrome (PCOS) is a multifactorial female endocrine disorder associated with environmental or genetic factors. It manifests clinically as infrequent menstruation, chronic or absent ovulation, hyperandrogenemia, and polycystic ovarian changes (1). As a multisystem disease, PCOS not only impacts reproductive function, but also increases the risk of obesity, type 2 diabetes, cardiovascular disease, and anxiety or depression (2–4), creating significant economic burdens on public health and families. An epidemiological study estimated that approximately 11-13% of women worldwide suffer from PCOS, and its prevalence has doubled over the past three decades (5). Among females aged 10–24 years, PCOS affects 6-18% of adolescents (6).

The onset of PCOS can be traced to adolescence, with common clinical symptoms (acne, menstrual irregularities, excessive weight gain) developing during this period (7). Adolescence is a critical window for endocrine regulation and metabolic remodeling in females, and lack of timely intervention may facilitate the progression of PCOS. With disease progression, the likelihood of developing metabolic disorders in adulthood increases (8). However, its symptoms are often overlooked or misdiagnosed during adolescence as they overlap with the physiological changes of puberty (9). Current studies on PCOS predominantly focus on women of reproductive age, leaving a scarcity of long-term epidemiological studies for females aged 10–24 years. Moreover, there is a lack of high-quality evidence tracking and comparing the incidence of PCOS among females aged 10–24 across different regions and countries.

This study aims to analyze the epidemiological characteristics of PCOS in females aged 10–24 using data from the Global Burden of Disease (GBD) study. It seeks to assess the burden of the disease and illuminate its global distribution and associated health impacts among adolescent females. Drawing from GBD 2021 data from 1990 to 2021, the study examines trends in the prevalence, incidence, and disability-adjusted life years (DALYs) of PCOS among adolescent and young women (ages 10-24) at global, regional, and national levels. In addition, this study stratifies trends by age group and sociodemographic index (SDI) to provide a scientific foundation for the early prevention and management of the disease.

## 2 Methods

### 2.1 Study design and data sources

GBD is among the most critical projects for understanding global disease burden (10). Led by the Institute for Health Metrics and Evaluation (IHME), it uses all available data to provide the most up-to-date and comprehensive descriptive epidemiological assessments of diseases for seven super-regions, 21 regions, and 204 countries and territories from 1990 to 2021. Health data is collected globally through collaborative efforts from sources such as life records, registries, censuses, health surveys, population monitoring, scientific research, administrative reports, discharge records, outpatient visits, health insurance claims, and many others. The input data is then processed using algorithms to generate disease burden estimates. All data was queried directly and downloaded from the GBD results tool. A detailed description of the methodology can be found on database help pages and other publications (10). The GBD study used the Bayesian meta-regression tool DisMod-MR 2.1 to generate disease estimates by age, year, and location, ensuring consistency across epidemiological parameters of the studied conditions (10).

This study is a retrospective analysis, and data on global burden of PCOS in adolescents and young adults were obtained using the Global Health Data Exchange query tool from published sources. Data on incidence, DALYs, and prevalence of PCOS from 1990 to 2021 were obtained from GBD 2021 at global, regional, national, and SDI quintile levels. GBD divides the SDI of 21 regions, 204 countries, and territories into five categories (high, high-middle, middle, low-middle, and low) based on total fertility rate, per capita income, and mean years of education (11). The SDI ranges from 0 to 1, with higher values indicating higher levels of socioeconomic development. GBD 2021 data was used to assess changes in disease burden across regions and age groups over time.

Currently, there is no universally accepted diagnostic standard for PCOS. Diagnosis is primarily based on three criteria: the National Institutes of Health (NIH), Rotterdam, and Androgen Excess Society (AES) guidelines. International Classification of Diseases (ICD) codes 10 (ICD-10 code: E28.2) and ICD-9 (E28.2) were used to identify PCOS cases.

It must be emphasized that this secondary analysis focused on adolescents. Adolescence refers to the life stage transitioning from childhood to adulthood, encompassing biological growth as well as social and behavioral factors. The World Health Organization defines adolescence as the period between 10 and 19 years of age. To better understand adolescent growth and this life stage comprehensively, the study further subdivided adolescents into younger adolescents (10–14 years) and older adolescents (15–19 years). Since characteristics of normal pubertal development overlap with adult diagnostic criteria, PCOS diagnosis in adolescents requires cautious judgment. In line with the latest international evidence-based guidelines, a re-evaluation is recommended three years after menarche. Therefore, PCOS data was collected from three age subgroups (10-14, 15-19, and 20–24 years).

### 2.2 Definition and explanation of analytical metrics

DALYs: The sum of years lived with disability (YLDs) and years of life lost (YLLs), representing the quality-of-life decline or long-term health problems caused by PCOS. Higher DALYs indicate a greater disease burden.

Number of new cases: The total number of newly diagnosed adolescent cases of PCOS from 1990 to 2021.

Crude Incidence Rate (CIR): The number of newly diagnosed PCOS cases in the adolescent population during 1990-2021, expressed as a ratio to the total population, typically per 100,000 people. It is a basic metric to measure disease occurrence in a specific region or population.

Age-Standardized Incidence Rate (ASIR): Incidence rate adjusted for differences in population age distribution, expressed per 100,000 people.

Number of prevalent cases: The total number of all existing cases of adolescent PCOS from 1990 to 2021.

Crude Prevalence Rate (CPR): The proportion of adolescent PCOS cases in the population during 1990-2021, expressed per 100,000 people.

Age-Standardized Prevalence Rate (ASPR): Prevalence rate adjusted for differences in population age distribution, expressed per 100,000 people.

### 2.3 Statistical analysis

Descriptive statistical analyses were performed using prevalence, incidence, and DALY counts and ASRs (per 100,000) as indicators to assess the adolescent burden of PCOS, with corresponding 95% uncertainty intervals (UIs). All statistical analyses and visualizations were conducted using R (version 4.4.2), with P < 0.05 considered statistically significant. The detailed statistical methodologies include:

#### 2.3.1 Prediction of prevalence, incidence, and DALYs per 100,000 people

Estimated values and 95% UIs were provided. A smoothed spline model assessed the relationship between disease burden and SDI across seven super-regions, 21 regions, and 204 countries/territories, calculating expected values based on SDI levels and disease incidence. Local regression smoothing fitted the spline model, automatically determining the degree, number, and arrangement of nodes based on data and span parameters. Spearman correlation analysis calculated correlation coefficients (r) and corresponding p-values to evaluate the relationship between ASRs and SDI. Statistical significance was defined as *P* < 0.05. All analyses and visualizations were performed using R software (version 4.4.2).

#### 2.3.2 Time trend analysis with Joinpoint regression

Joinpoint regression analysis was employed to evaluate changes in ASIR, ASPR, DALY, CIR, crude DALY rate (CDR), and CPR during the study period. Using the Joinpoint regression program (version 4.8.0.1), points of significant change in trends were determined, calculating average annual percent change (AAPC) with corresponding 95% confidence intervals (95% CI). This method identifies significant trend changes, aiding in interpreting time patterns.

#### 2.3.3 Frontier analysis

To assess the relationship between PCOS burden and sociodemographic development, data from 1990 to 2021 was used to establish frontier analyses for ASIR, ASPR, and ASDR (Age-Standardized DALY Rate) against the SDI. This approach provided insight into the potential improvements in PCOS incidence and prevalence that a country or region may achieve.

#### 2.3.4 Future trend projection using ARIMA model

The autoregressive integrated moving average (ARIMA) model predicted PCOS burden trends among adolescents from 2021 to 2035. The ARIMA model, implemented in R (version 4.4.2) with the “forecast” package, is a robust time-series analysis tool enabling future value predictions based on historical data patterns. Model accuracy was assessed using the Akaike information criterion and Bayesian information criterion (BIC).

### 2.4 Data quality and uncertainty

All estimates were expressed as 95% UIs, calculated using Monte Carlo simulations. These intervals account for uncertainties in input data and modeling processes, providing a range where the true value is likely to fall. Including UIs is crucial for interpreting the reliability and accuracy of the study results.

## 3 Results

### 3.1 Global trends

**Figure 1:**
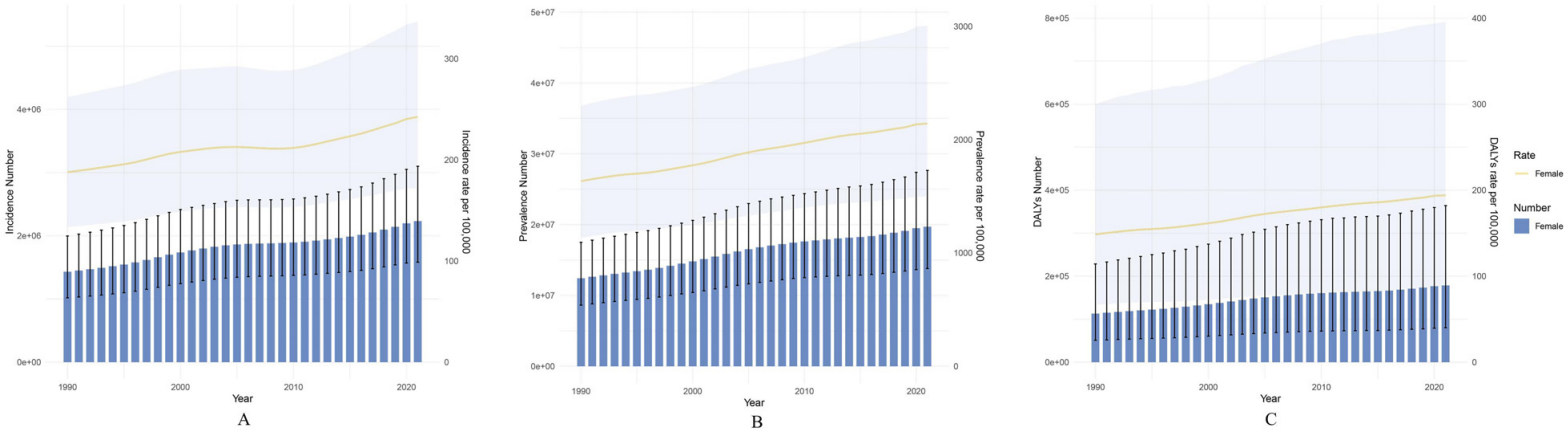
Global trends in the disease burden of PCOS among females aged 10–24 years from 1990 to 2021 (at ten-year intervals): **(A)** Changes in the number of PCOS cases and incidence rate among females aged 10–24 years; **(B)** Changes in the number of PCOS cases and prevalence rate among females aged 10–24 years; **(C)** Changes in the number of PCOS DALYs and DALY rate among females aged 10–24 years. The shaded areas represent the 95% confidence intervals.

Globally, the PCOS incidence among adolescent and young women increased by 56.02%, from 1,430,611 cases (95% UI: 1,017,284-1,996,173) in 1990 to 2,232,036 cases (95% UI: 1,581,502-3,099,410) in 2021 (**Table 1**). Concurrently, global prevalence among adolescent and young women increased by 58.55%, from 12,431,336 cases (95% UI: 8,626,816-17,498,544) in 1990 to 19,709,918 cases (95% UI: 13,796,725-27,652,540) in 2021 (**Supplementary Table S1**). Furthermore, DALYs attributed to PCOS among adolescent and young females reached 178,473 (95% UI: 80,140-363,894) in 2021, up approximately 57.93% from 113,006 (95% UI: 50,849-228,343) in 1990 (**Supplementary Table S2**). Overall, PCOS incidence, prevalence, and DALY rates among adolescent and young women exhibited varying degrees of increase over the past three decades (**Figure 1**). Notably, after age-standardization, the global age-standardized incidence rate increased from 49.45 per 100,000 (95% UI: 35.57-68.45) in 1990 to 63.26 per 100,000 (95% UI: 45.41-87.28) in 2021, with an average annual percent change (AAPC) of 0.8% (95% CI: 0.77-0.831). Age-standardized prevalence and age-standardized DALY rates also showed an upward trend (**Supplementary Figure S1**, **Supplementary Table S3**).

**Table 1:** Incidence and AAPC of PCOS in adolescents and young adults aged 10–24 years at global and regional level, 1990-2021.

	Cases (n), 1990	incidence (per 100,000 population), 1990	Cases (n), 2021	incidence (per 100,000 population), 2021	AAPC (95%CI)	*P* -value
**Global**	1430611 (1,017,284-1,996,173)	187.97 (133.66-262.27)	2,232,036 (1,581,502-3,099,410)	242.53 (171.84-336.77)	0.837(0.797-0.877)	
SDI level						
High SDI	431,649 (313,761-603,127)	452.58 (328.97-632.37)	488,055 (361,230-666,624)	543.58 (402.32-742.46)	0.586(0.489-0.682)	<0.001
High-middle SDI	251,516 (177,567-350,684)	180.67 (127.55-251.91)	294,924 (206,540-416,104)	274.55 (192.27-387.36)	1.372(1.319-1.425)	<0.001
Low SDI	58,180 (39,760-83,797)	75.2 (51.39-108.31)	196,534 (135,391-282,567)	106.86 (73.61-153.64)	1.154(1.103-1.206)	<0.001
Low-middle SDI	199,580 (138521-284257)	111.84 (77.62-159.29)	462,614 (322,035-653,298)	170.5 (118.69-240.77)	1.381(1.311-1.451)	<0.001
Middle SDI	488,690 (342973-688057)	180.98 (127.01-254.81)	788,280 (550,751-1,104,280)	295.04 (206.13-413.31)	1.598(1.553-1.642)	<0.001
GBD super regions						
Central Europe, Eastern Europe, and Central Asia	15,486 (10,388-22,874)	32.71 (21.94-48.31)	16,503 (11,343-23,842)	46.33 (31.84-66.93)	1.137(1.1-1.173)	<0.001
High-income	522,702 (382,162-721,592)	524.77 (383.68-724.45)	554,201 (409,003-754,307)	596.8 (440.44-812.29)	0.394(0.311-0.478)	<0.001
Latin America and Caribbean	172,892 (118,671-247,854)	274.23 (188.23-393.12)	216,578 (151,004-305,373)	304.19 (212.09-428.9)	0.337(0.264-0.41)	<0.001
North Africa and Middle East	126,319 (86,758-181,218)	237.47 (163.1-340.67)	237,511 (165,811-338,089)	302.29 (211.03-430.3)	0.822(0.752-0.892)	<0.001
South Asia	152,142 (105,801-216,076)	94.25 (65.54-133.85)	395,036 (275,861-553,520)	155.26 (108.42-217.55)	1.659(1.581-1.737)	<0.001
Southeast Asia, East Asia, and Oceania	376,315 (260,123-529,247)	146.63 (101.36-206.22)	609,755 (425,410-860,687)	306.65 (213.95-432.85)	2.435(2.347-2.524)	<0.001
Sub-Saharan Africa	64,755 (44,236-93,795)	81.1 (55.4-117.47)	202,453 (139,818-291,702)	107.23 (74.05-154.5)	0.937(0.893-0.98)	<0.001
GBD regions						
Andean Latin America	25,024 (17,074-35,835)	404.33 (275.86-579)	42,169 (29,111-60,035)	504.5 (348.27-718.24)	0.715(0.678-0.752)	<0.001
Australasia	15,827 (11,635-20,710)	669.94 (492.5-876.61)	21,325 (15,273-29,426)	764.81 (547.76-1055.36)	0.422(0.319-0.526)	<0.001
Caribbean	8,984 (6,067-12,796)	167.12 (112.86-238.05)	11,645 (7,832-16,495)	207.88 (139.82-294.47)	0.72(0.682-0.759)	<0.001
Central Asia	5,012 (3,348-7,377)	50.99 (34.06-75.05)	7,591 (5,115-10,823)	70.64 (47.6-100.72)	1.066(1.033-1.098)	<0.001
Central Europe	3,858 (2,485-5,908)	26.99 (17.39-41.34)	2,876 (1,949-4,108)	32.69 (22.16-46.71)	0.627(0.603-0.651)	<0.001
Central Latin America	117,939 (80,753-167,746)	429.49 (294.07-610.87)	141,023 (98,475-198,486)	436.55 (304.84-614.43)	0.055(-0.11-0.221)	0.514
Central Sub-Saharan Africa	5,728 (3,867-8,359)	66.43 (44.85-96.95)	22,990 (15,738-33,397)	102.86 (70.41-149.42)	1.454(1.358-1.551)	<0.001
East Asia	204,584 (140,815-288,916)	112.8 (77.64-159.3)	254,080 (176,216-361,742)	223.96 (155.32-318.86)	2.293(2.129-2.457)	<0.001
Eastern Europe	6,617 (4,324-9,804)	28.49 (18.62-42.22)	6,037 (3,996-8,964)	37.54 (24.85-55.75)	0.903(0.86-0.946)	<0.001
Eastern Sub-Saharan Africa	24,484 (16,750-35,517)	78.04 (53.39-113.21)	73,146 (50,646-105,169)	100.33 (69.47-144.26)	0.828(0.795-0.862)	<0.001
High-income Asia Pacific	157,699 (110,282-225,568)	768.58 (537.48-1099.36)	106,110 (74,706-151,102)	836.26 (588.76-1190.84)	0.285(0.243-0.328)	<0.001
High-income North America	132,380 (92,787-185,703)	442.82 (310.38-621.19)	201,569 (148,264-269,547)	578.26 (425.34-773.28)	0.814(0.617-1.011)	<0.001
North Africa and Middle East	126,319 (86,758-181,218)	237.47 (163.1-340.67)	237,511 (165,811-338,089)	302.29 (211.03-430.3)	0.822(0.752-0.892)	<0.001
Oceania	1,970 (1,357-2,813)	195.28 (134.51-278.83)	5,205 (3,598-7,335)	270.69 (187.1-381.48)	1.047(0.999-1.095)	<0.001
South Asia	152,142 (105,801-216,076)	94.25 (65.54-133.85)	395,036 (275,861-553,520)	155.26 (108.42-217.55)	1.659(1.581-1.737)	<0.001
Southeast Asia	169,761 (117,787-239,852)	228.61 (158.62-323)	350,470 (247,876-490,068)	419.89 (296.97-587.14)	2.007(1.959-2.056)	<0.001
Southern Latin America	12,176 (8,366-17,425)	184.07 (126.47-263.42)	21,189 (14,925-30,636)	280.49 (197.56-405.53)	1.371(1.302-1.441)	<0.001
Southern Sub-Saharan Africa	11,281 (7,793-16,240)	128.95 (89.08-185.63)	17,465 (11,923-25,132)	161.03 (109.93-231.72)	0.754(0.689-0.819)	<0.001
Tropical Latin America	20,946 (13,911-30,845)	87.1904 (57.91-128.4)	21,741 (14,767-30,877)	87.1927 (59.22-123.83)	0.018(-0.151-0.187)	0.839
Western Europe	204,619 (144,424-284,521)	508.81 (359.13-707.5)	204,009 (143,384-285,256)	583.34 (409.99-815.65)	0.442(0.414-0.469)	<0.001
Western Sub-Saharan Africa	23,263 (15,880-33,933)	74.79 (51.05-109.09)	88,851 (61,143-128,440)	107.43 (73.93-155.3)	1.188(1.08-1.297)	<0.001

**Figure 2:**
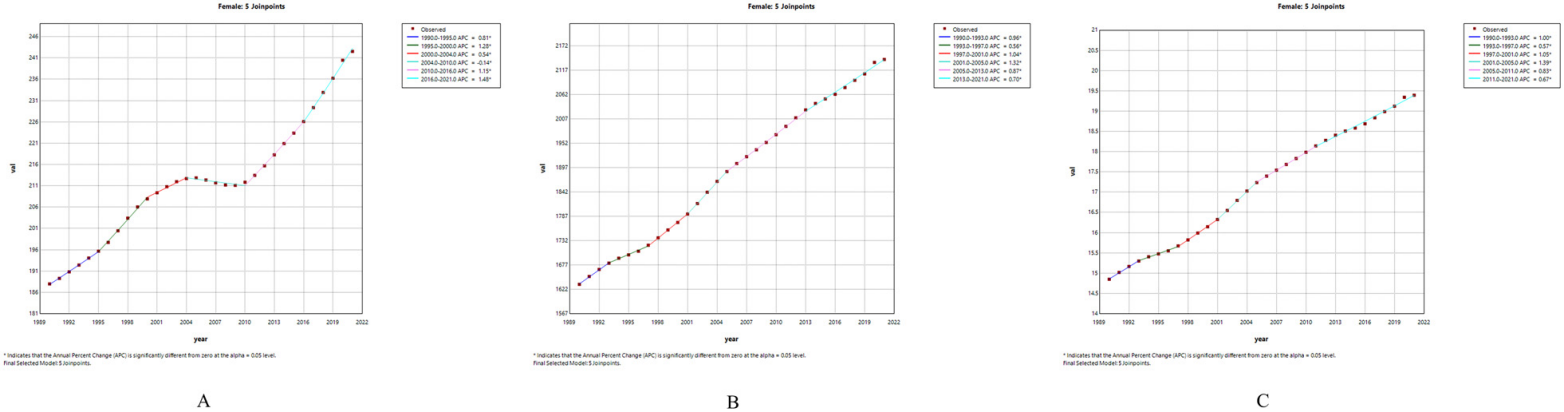
Joinpoint regression analysis of PCOS among females aged 10–24 years globally from 1990 to 2021: **(A)** Trends in incidence rate; **(B)** Trends in prevalence rate; **(C)** Trends in DALYs rate.

To further clarify the trends in PCOS incidence among adolescents and young women over the past three decades, we found that during 1990-2000, the incidence of PCOS increased rapidly (AAPC 1.04 [95% CI 0.971 - 1.109]). From 2001 to 2010, the upward trend continued, although at a slower rate (AAPC 0.084 [95% CI 0.008 - 0.159]). However, during 2011-2021, the incidence showed the fastest rate of increase (AAPC 1.314 [95% CI 1.26 - 1.369]). (**Supplementary Table S4**) Joinpoint regression analysis identified significant changes in PCOS incidence in 1995, 2000, 2004, 2010, and 2016 (**Figures 2A–C**).

### 3.2 Global trends by age group

**Figure 3:**
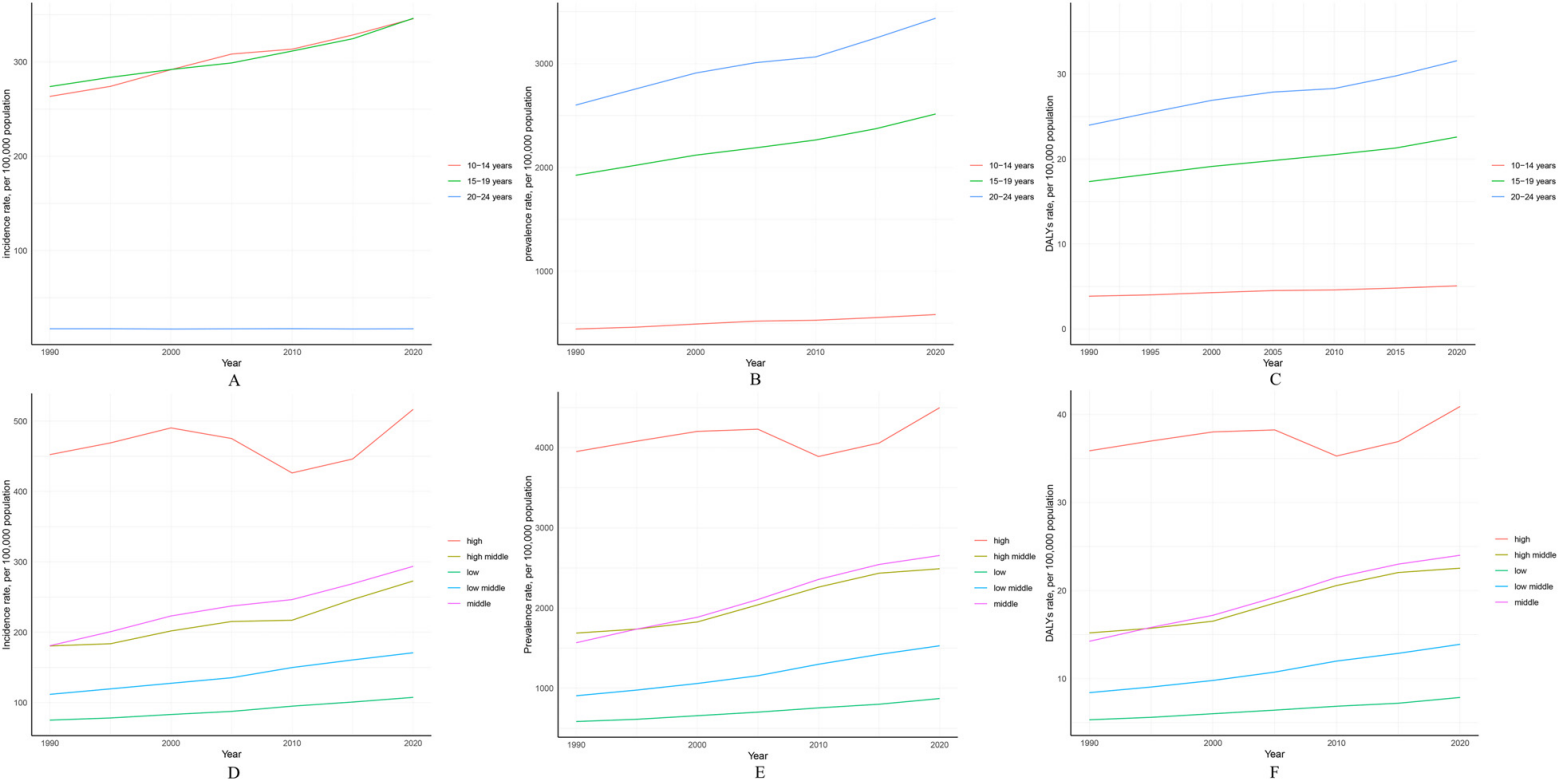
Global trend analysis of PCOS among females aged 10–24 years from 1990 to 2021 (by age groups and SDI quintiles): **(A)** Changes in incidence rate by age group; **(B)** Changes in prevalence rate by age group; **(C)** Changes in DALY rate by age group; **(D)** Changes in incidence rate by SDI quintiles; **(E)** Changes in prevalence rate by SDI quintiles; **(F)** Changes in DALY rate by SDI quintiles.

Globally, during the period from 1990 to 2021, the PCOS incidence rate increased most significantly in the 10–14 age group, from 263.49/100,000 (95% UI 137.65-437.39) in 1990 to 348.52/100,000 (95% UI 185.23-566.54) in 2021, with an AAPC of 0.905 (95% UI 0.843-0.967). The PCOS incidence rate also increased in the 15–19 age group, from 273.92/100,000 (95% UI 168.93-435.15) in 1990 to 347.88/100,000 (95% UI 217.07-540.95) in 2021, with an AAPC of 0.786 (95% UI 0.758-0.815). However, the PCOS incidence rate in the 20–24 age group changed minimally during the same period, from 17.13/100,000 (95% UI 7.16-40.96) to 17.1/100,000 (95% UI 6.97-42.46), with an AAPC of -0.01 (95% UI -0.03-0.01) (**Supplementary Table S5**). In summary, age-specific incidence rates of PCOS were highest in the 10–14 and 15–19 age groups and decreased with increasing age (**Figure 3A**).

Conversely, for the same period, the PCOS age-specific prevalence and DALY rates increased more prominently in the 20–24 age group (**Figures 3B, C**). Prevalence rose from 2,600.45/100,000 (95% UI 1,849.19-3,620.8) in 1990 to 3,453.97/100,000 (95% UI 2,489.13-4,742.61) in 2021, with an AAPC of 0.927 (95% UI 0.891-0.963). Additionally, DALY rates increased from 23.99/100,000 (95% UI 10.67-50.22) in 1990 to 31.71/100,000 (95% UI 14.2-65.97) in 2021, with an AAPC of 0.909 (95% UI 0.871-0.947). Global trends in age-specific PCOS incidence, prevalence, and DALY rates are detailed in the appendix (**Supplementary Figure S2**).

### 3.3 Global trends by SDI

From 1990 to 2021, among all SDI regions, the middle SDI region experienced the largest increase in PCOS incidence, rising from 180.98/100,000 (95% UI 127.01-254.81) in 1990 to 295.04/100,000 (95% UI 206.13-413.31) in 2021, with an AAPC of 1.598 (95% UI 1.553-1.642). In contrast, the high SDI region experienced the smallest increase in PCOS incidence, rising from 452.58/100,000 (95% UI 328.97-632.37) in 1990 to 543.58/100,000 (95% UI 402.32-742.46) in 2021, with an AAPC of 0.586 (95% UI 0.489-0.682) (**Table 1**, **Supplementary Figure S3**).

Furthermore, across all SDI regions, the burden of PCOS in adolescents and young adults increased during this period. Notably, the high SDI region maintained the highest PCOS incidence, prevalence, and DALY rates throughout, while the low SDI region consistently had the lowest rates (**Figures 3D-F**, **Supplementary Figure S3**). Additionally, to better represent the global burden of PCOS in adolescents and young women across SDI regions, age-standardized results reveal similar trends in changes in PCOS incidence, prevalence, and DALY rates across distinct SDI regions (**Supplementary Figure S4**, **Supplementary Table S3**).

### 3.4 Regional trends

The GBD regional classification system encompasses 7 super-regions and 21 regions, highlighting significant differences in the prevalence, incidence, and DALYs of PCOS among adolescents. Notably, between 1990 and 2021, Southeast Asia, East Asia, and Oceania experienced the largest increase in PCOS incidence among adolescents and young women across the super-regions (rising from 146.63 per 100,000 [95% UI 101.36-206.22] in 1990 to 306.65 per 100,000 [95% UI 213.95-432.85] in 2021; AAPC 2.435 [95% UI 2.347-2.524]). In contrast, the Latin America and Caribbean region showed the smallest increase in incidence (increasing from 274.23 per 100,000 [95% UI 188.23-393.12] in 1990 to 304.19 per 100,000 [95% UI 212.09-428.9] in 2021; AAPC 0.337 [95% UI 0.264-0.41]) (**Table 1**). Furthermore, the analysis revealed that among the 7 super-regions, Southeast Asia, East Asia, and Oceania also had the largest increases in both prevalence and DALYs rates (prevalence rising from 1,330.41 per 100,000 [95% UI 922.68-1,904.28] in 1990 to 2,696.22 per 100,000 [95% UI 1,856.12-3,825.58] in 2021; AAPC 2.316 [95% UI 2.273-2.358]; DALYs rate rising from 12.03 per 100,000 [95% UI 5.22-24.96] in 1990 to 24.47 per 100,000 [95% UI 11-50.48] in 2021; AAPC 2.332 [95% UI 2.282-2.382]). Among the 7 super-regions, the High-income region exhibited the smallest increase in prevalence and DALYs rates (AAPC 0.266 [95% UI 0.187-0.344]) (**Supplementary Tables S1**, **S2**). Overall, the prevalence, incidence, and DALYs rates for all GBD super-regions displayed an upward trend.

**Figure 4:**
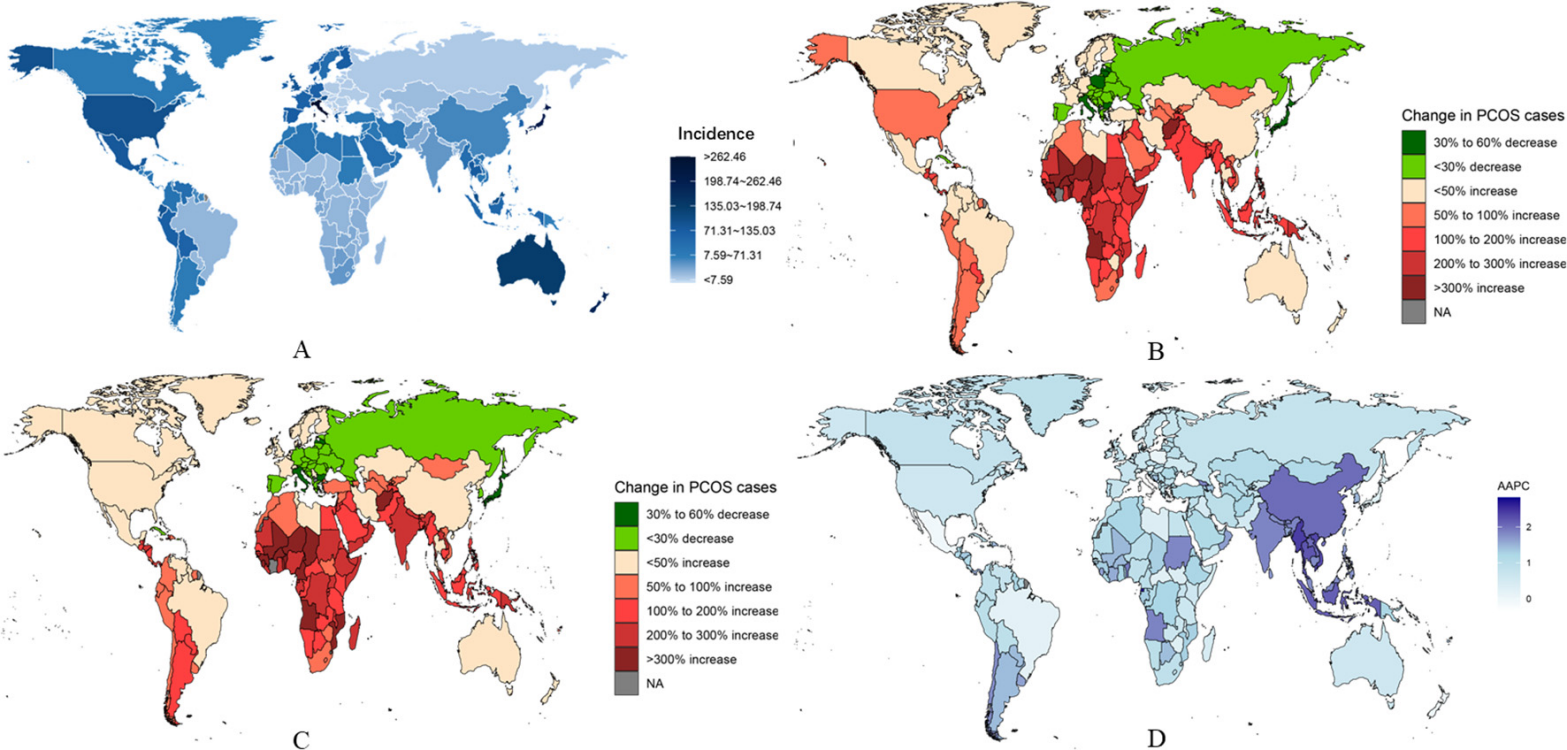
National burden of PCOS. **(A)** Estimated incidence rates in 2021. **(B)** Trends in incidence rates from 1990 to 2019. **(C)** Trends in prevalence rates from 1990 to 2021. **(D)** Average annual percentage change (AAPC) in incidence rates by country.

Specifically, the top three GBD regions with the largest increases in PCOS incidence among adolescents and young women were East Asia (rising from 112.8 per 100,000 [95% UI 77.64-159.3] in 1990 to 223.96 per 100,000 [95% UI 155.32-318.86] in 2021; AAPC 2.293 [95% UI 2.129-2.457]), Southeast Asia (rising from 228.61 per 100,000 [95% UI 158.62-323] in 1990 to 419.89 per 100,000 [95% UI 296.97-587.14] in 2021; AAPC 2.007 [95% UI 1.959-2.056]), and South Asia (rising from 94.25 per 100,000 [95% UI 65.54-133.85] in 1990 to 155.26 per 100,000 [95% UI 108.42-217.55] in 2021; AAPC 1.659 [95% UI 1.581-1.737]). The region with the smallest growth in incidence was Tropical Latin America, with minimal changes (AAPC 0.018 [95% UI -0.151 to 0.187]) (**Figure 4**). It is worth noting that in 2021, High-income Asia Pacific had the highest incidence of PCOS among adolescents and young women, at 836.26 per 100,000 [95% UI 588.76-1,190.84] (**Table 1**). Additionally, to more accurately describe the burden of PCOS among adolescents and young women across the 7 super-regions and 21 regions globally, age-standardized analyses revealed similar trends in PCOS incidence, prevalence, and DALYs rates across regions. The age-standardized regional trends in PCOS incidence, prevalence, and DALYs rates, along with associated AAPCs from 1990 to 2021, are provided in the appendix (**Supplementary Table S3**).

### 3.5 National trends

From 1990 to 2021, the greatest increases in PCOS incidence among adolescents and young women were observed in Equatorial Guinea (rising from 69.19 per 100,000 [95% UI 46.61-102.15] in 1990 to 163.78 per 100,000 [95% UI 109.92-234.64] in 2021; AAPC 2.835 [95% UI 2.651-3.019]), Maldives (rising from 215.82 per 100,000 [95% UI 148.62-304.31] in 1990 to 498.19 per 100,000 [95% UI 344.99-713.13] in 2021; AAPC 2.768 [95% UI 2.685-2.851]), and Myanmar (rising from 183.72 per 100,000 [95% UI 127.62-263.82] in 1990 to 377.38 per 100,000 [95% UI 267.02-534.49] in 2021; AAPC 2.383 [95% UI 2.274-2.491]). In contrast, in 2021, the countries with the highest PCOS incidence among adolescents and young women were Italy (1,225.13 per 100,000 [95% UI 857.51-1,720.08]), Japan (1,012.94 per 100,000 [95% UI 714-1,435.76]), and New Zealand (889.53 per 100,000 [95% UI 629.43-1,231.24]). Conversely, the countries with the lowest PCOS incidence in 2021 were Albania (25.84 per 100,000 [95% UI 16.83-38.89]), Bosnia and Herzegovina (26.4 per 100,000 [95% UI 17.28-39.61]), and North Macedonia (26.72 per 100,000 [95% UI 17.38-40.39]) (**Supplementary Table S14**).

During the same period, Equatorial Guinea, Maldives, and Myanmar also experienced the highest increases in prevalence and DALYs rates(Fiugre 4 and Fiugre S6) (prevalence in Equatorial Guinea rose from 553.71 per 100,000 [95% UI 360.3-860.55] in 1990 to 1,417.57 per 100,000 [95% UI 940.83-2,047.82] in 2021; AAPC 3.084 [95% UI 2.867-3.302]; prevalence in Maldives rose from 1,731.3 per 100,000 [95% UI 1,183.04-2,501.81] in 1990 to 4,249.36 per 100,000 [95% UI 2,848.67-6,218.7] in 2021; AAPC 2.932 [95% UI 2.722-3.142]; prevalence in Myanmar rose from 1,496.46 per 100,000 [95% UI 1,032.06-2,148.29] in 1990 to 3,254.11 per 100,000 [95% UI 2,267.67-4,643.55] in 2021; AAPC 2.573 [95% UI 2.486-2.663]). Changes in DALYs rates were also significant in these countries (e.g., DALYs in Equatorial Guinea rose from 4.96 per 100,000 [95% UI 2.05-10.55] in 1990 to 12.74 per 100,000 [95% UI 5.54-26.64] in 2021; AAPC 3.097 [95% UI 2.858-3.337]; DALYs in Maldives rose from 16.25 per 100,000 [95% UI 7-33.77] in 1990 to 38.91 per 100,000 [95% UI 16.9-84.39] in 2021; AAPC 2.866 [95% UI 2.657-3.075]; DALYs in Myanmar rose from 13.85 per 100,000 [95% UI 5.92-28.11] in 1990 to 29.95 per 100,000 [95% UI 13.4-61.78] in 2021; AAPC 2.553 [95% UI 2.468-2.638]) (**Figure 4**). Moreover, in 2021, the countries with the highest PCOS prevalence and DALYs rates among adolescents and young women were Italy (2021 prevalence: 11,139.35 per 100,000 [95% UI 7,848.9-15,788.9]; 2021 DALYs: 100.84 per 100,000 [95% UI 44.61-213.76]), Japan (2021 prevalence: 7,978.35 per 100,000 [95% UI 5,710.36-10,814.25]; 2021 DALYs: 71.14 per 100,000 [95% UI 31.55-143.42]), and New Zealand (2021 prevalence: 7,573.17 per 100,000 [95% UI 5,338.79-10,428.16]; 2021 DALYs: 67.57 per 100,000 [95% UI 30.14-140.46]) (**Supplementary Tables S6**, **S7**).

Notably, Italy, Mexico, and New Zealand have shown a decreasing trend in incidence rates over the past three decades. Among them, Italy is the only country that experienced a decline in incidence rates (AAPC -0.049 [95% UI -0.143 to 0.044]), prevalence rates (AAPC -0.309 [95% UI -0.368 to -0.249]), and DALYs rates (AAPC -0.245 [95% UI -0.326 to -0.165]) between 1990 and 2021. Furthermore, in order to better describe the burden of PCOS in adolescent and young adult females across 204 countries globally, age-standardized analysis revealed that trends in incidence rates, prevalence rates, and DALYs rates were similar across different regions. For more specific information, refer to the appendix (**Supplementary Table S8**).

A. Estimated incidence rates in 2021.B. Trends in incidence rates from 1990 to 2019.C. Trends in prevalence rates from 1990 to 2021.D. Average annual percentage change (AAPC) in incidence rates by country.

### 3.6 Future forecasts of global burden of PCOS

**Figure 5:**
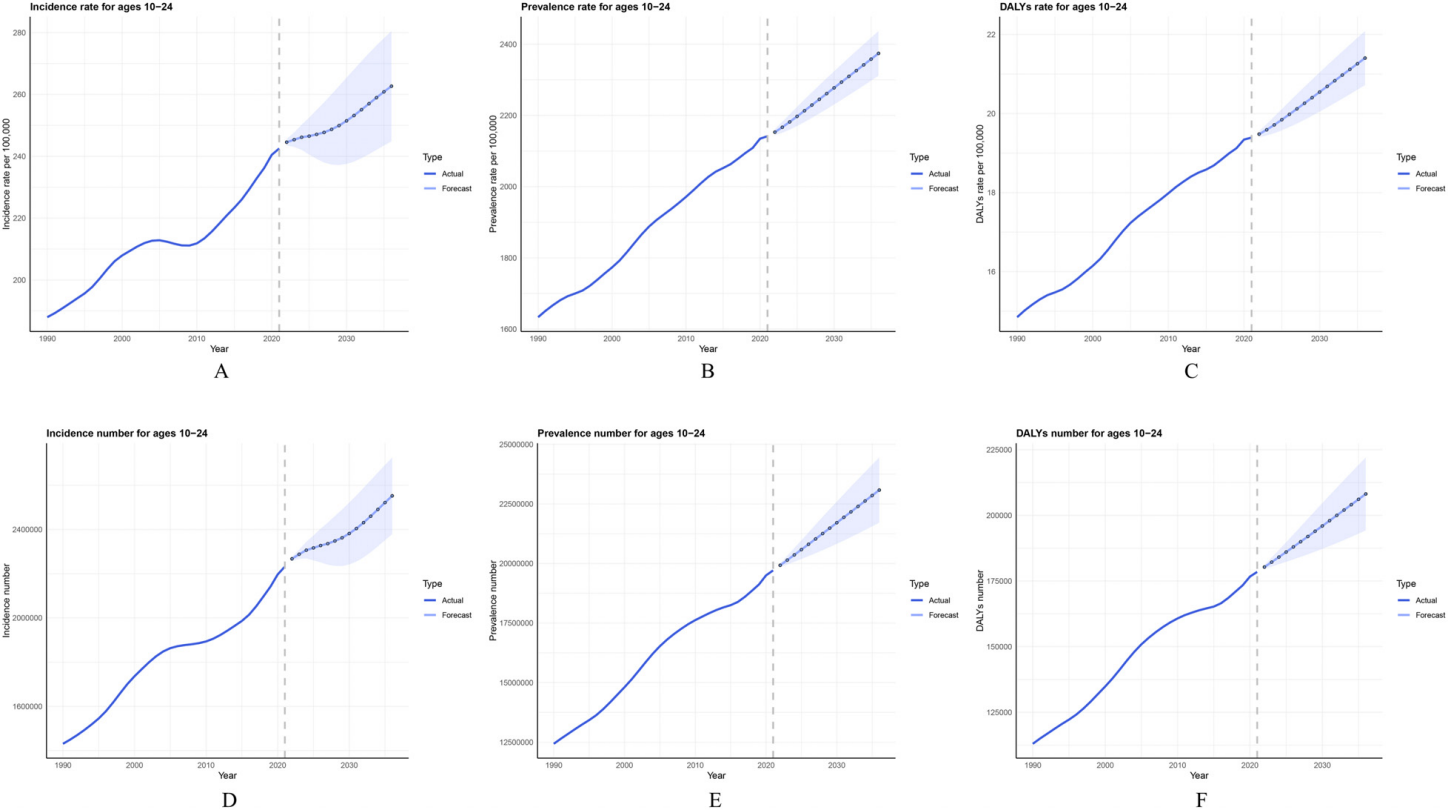
Trends and projections of PCOS incidence, prevalence, and DALYs rate among females aged 10–24 years from 1990 to 2036: **(A)** Actual and projected values of PCOS incidence rate; **(B)** Actual and projected values of PCOS prevalence rate; **(C)** Actual and projected values of PCOS DALYs rate; **(D)** Actual and projected values of PCOS incidence cases; **(E)** Actual and projected values of PCOS prevalence cases; **(F)** Actual and projected values of PCOS DALYs cases. Solid lines represent actual data from 1990 to 2021, while dashed lines and markers represent forecasts from 2022 to 2036. Error bars indicate the uncertainty range of the projections.

From 2022 to 2036, the global burden of PCOS among adolescents and young women aged 10–24 is projected to undergo significant changes, with all indicators showing an upward trend (**Figures 5A–F**). The global incidence of PCOS in this age group is expected to increase gradually, with the estimated number of incident cases reaching 2,552,342 by 2036, resulting in an incidence rate of 262.69 per 100,000. This represents an 8.32% increase compared to the 2021 rate of 242.52 per 100,000. Similarly, projections for the prevalence of PCOS among adolescents and young women globally indicate an upward trend over the same period. By 2036, the total number of prevalent cases is estimated to reach 23,081,840, with the prevalence rate projected to rise from 2,141.62 per 100,000 in 2021 to 2,374.32 per 100,000 in 2036, marking a 10.87% increase compared to 2021 levels.

Additionally, the DALYs rate for PCOS is projected to increase from 19.39 per 100,000 in 2021 to 21.4 per 100,000 by 2036 (**Supplementary Table S9**), underscoring the growing health impact associated with PCOS in adolescents and young women globally. After age-standardization to account for population structure changes, projections of ASIR, ASPR, and ASDR over the next 15 years consistently demonstrate upward trends across all indicators (**Supplementary Figure S5**).

### 3.7 Frontier analysis of PCOS burden

The frontier represents leading countries or regions (pushing the envelope at the frontier), with their SDI associated with the lowest disease burden. Effective difference is defined as the distance from the frontier, representing the gap between a country or region’s observed disease burden and the potentially achievable disease burden based on its SDI. A significant effective difference from the frontier indicates that, given a country or region’s position within the development spectrum, there may be unrealized benefits or opportunities for improvement (reductions in PCOS disability-adjusted life years or DALYs).

**Figure 6:**
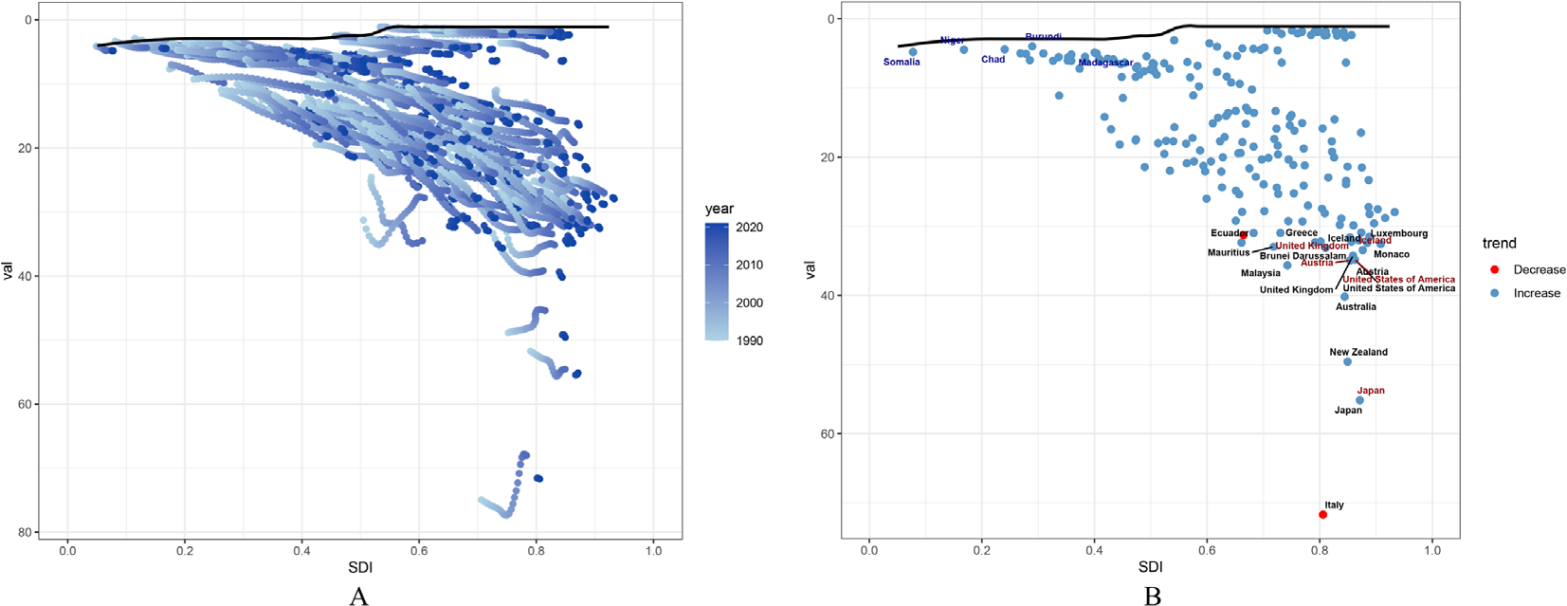
Frontier analysis of PCOS DALYs rate and SDI among females aged 10–24 years: **(A)** Frontier analysis of SDI and PCOS DALYs rate from 1990 to 2021; **(B)** Frontier analysis of SDI and PCOS DALYs rate in 2021.

We used 2021 DALYs and SDI data to estimate the effective difference between each country or region and the frontier (**Figure 6**, **Supplementary Table S10**). The 15 countries or regions with the largest effective differences from the frontier (range: 71.69-32.31) include Italy, Japan, New Zealand, Australia, Malaysia, Austria, United States of America, United Kingdom, Iceland, Brunei Darussalam, Mauritius, Monaco, Luxembourg, Ecuador, and Greece (**Supplementary Table S11**).

Conversely, the five countries or regions with the lowest effective differences (range: 3.98-4.89), reflecting the lowest DALY rates given their position on the development spectrum, were Burundi, Chad, Niger, Madagascar, and Somalia (**Supplementary Table S12**). Additionally, among high-SDI countries, the top five countries or regions with the highest effective differences (range: 55.14-33.43) were Japan, Austria, United States of America, United Kingdom, and Iceland (**Supplementary Table S13**).

The black solid line represents the frontier, with points representing countries and regions. Blue dots indicate an upward trend, while red dots indicate a downward trend.

## 4 Discussion

### 4.1 Principal findings

PCOS is a chronic, complex, and under-recognized disorder, with its onset often traceable to the initiation of puberty (12). At this time, overlap with pubertal symptoms is a common issue for adolescents and young women at risk of PCOS (13). Relevant studies suggest that rapid ectopic fat accumulation disrupts homeostatic effects in the body, leading to dysfunctions in the endocrine axis and triggering endocrine-metabolic imbalances (14), which subsequently manifest as ovulatory dysfunction, acne, hirsutism, irregular menstruation, and other symptoms associated with PCOS (15). This study represents the first comprehensive analysis of the prevalence, incidence, and DALYs of PCOS among adolescents and young women aged 10–24 across 204 countries worldwide from 1990 to 2021. The study findings indicate that although there was a decline between 2004 and 2010 (APC -0.14), the global incidence rate of PCOS among individuals aged 10–24 significantly increased from 187.97 to 242.53 per 100,000 people between 1990 and 2021, reflecting the growing impact of PCOS in this demographic. Prevalence and DALY rates also mirrored this upward trend, underlining the escalating health impacts of PCOS.

Spatiotemporal analysis revealed that high-income regions bear the highest burden of PCOS, which may be closely linked to lifestyle factors. Differences across regions could be attributed to variations in dietary habits, lifestyle patterns, levels of economic development, access to healthcare, and cultural practices (16). Additionally, findings showed that the burden of PCOS among adolescents and young women correlated with age and socioeconomic development index (SDI) levels. Different age groups exhibited distinct patterns of disease burden. The results highlight puberty as the earliest critical period when PCOS may manifest, a time closely tied to the physiological and metabolic changes occurring during puberty. The data suggest a strong link between PCOS and endocrine development as well as metabolic fluctuations within the first two years after menarche.

However, due to inconsistent diagnostic criteria and insufficient data in low- and middle-SDI countries, the global PCOS burden may be underestimated. Nonetheless, the trend of PCOS among 10–24-year-olds is evident, demanding urgent intervention for this demographic.

### 4.2 Results - in context of what is known

In high-SDI and high-income regions (e.g., Italy, Japan, and Australia), the high incidence of PCOS is likely linked to lifestyle factors, particularly higher intake of high-sugar and saturated fat-rich foods (17, 18). Such dietary habits may trigger obesity and insulin resistance (19), further influencing hormone levels, growth, and sexual maturation (20, 21), thereby contributing to reversible endocrine-metabolic dysfunctions. Additionally, exposure to endocrine-disrupting chemicals (e.g., Bisphenol A) during urbanization has been identified as a possible contributing factor to PCOS development. Eleni Palioura et al. identified potential mechanisms by which Bisphenol A disrupts hormone synthesis and metabolism, ovarian follicle maturation, lipid metabolism, and increases insulin resistance, contributing to hormonal imbalances associated with PCOS (22, 23).

Despite these risks, advanced healthcare systems in high-income countries and a higher awareness of women’s health often facilitate early detection, diagnosis, and management of PCOS during adolescence. However, unhealthy dietary patterns, sedentary lifestyles, and widespread stress among women in these regions make PCOS a notable public health issue in high-SDI countries. In moderate-SDI regions, the incidence and burden of PCOS are growing rapidly. Contributing factors may include heightened urbanization, improved health awareness, new diagnostic technologies, dietary/lifestyle shifts (e.g., increased fast food consumption and pollutant exposures) (24–26), and challenges in long-term post-diagnosis management. These factors potentially drive higher PCOS incidences and increase the likelihood of severe long-term complications. For instance, countries in Southeast Asia and East Asia are experiencing prominent PCOS growth among adolescents and young women due to rapid urbanization, lifestyle transitions, and greater health awareness (27, 28). These regions are characterized by increased intake of processed foods and sedentary routines, further exacerbating the rise in PCOS cases. Although the burden of PCOS in low-SDI regions remains the lowest, it has shown a steady increase over the past three decades. This may be related to delays in diagnosis and treatment due to insufficient healthcare resources in these countries (29). Additionally, lower industrialization and slower adoption of processed foods may reduce exposure to potential PCOS-promoting factors in these regions. However, studies have noted that socioeconomic status (SES) is closely tied to healthcare accessibility (30), with higher SES often enabling women to have greater diagnostic opportunities. Consequently, in resource-limited low-SDI areas, PCOS prevention and treatment may not be prioritized in public health initiatives, masking the demand for early diagnosis and intervention.

From a demographic perspective, in developing countries with high fertility rates (such as parts of Africa, South America, and Southeast Asia), the ongoing expansion of the female population base leads to a passive increase in PCOS case numbers. The sheer growth of the young female population alone can elevate the total number of cases, even if no significant rise in age-standardized prevalence is observed. This phenomenon may be obscured under low-screening conditions: limited diagnostic capacity in resource-constrained settings could result in underestimation of the true prevalence, causing the apparent growth in PCOS cases to reflect the statistical outcome of population expansion rather than a genuine change in biological risk. In contrast, in high-income countries with advanced screening infrastructure and relatively stable population sizes, reported increases in case numbers more directly indicate growth in true prevalence driven by environmental or metabolic risk factors. This contrast underscores that in regions undergoing rapid demographic transitions, relying solely on prevalence estimates may underestimate the health burden of PCOS among females aged 10–24, necessitating layered analysis incorporating dynamic shifts in population age structure.

Age-wise, the findings reiterated that puberty is a critical stage for PCOS onset. Adolescents aged 10–14 showed the highest growth in incidence rates among specific age groups, while young individuals aged 20–24 exhibited the highest prevalence and DALYs rates. These results align with prior observations, reinforcing the notion that puberty—a key period for reproductive system maturation—is intrinsically linked to the emergence of PCOS. Hormonal fluctuations during puberty, including androgen excess and insulin resistance, coupled with unstable endocrine systems, accentuate metabolic irregularities, further driving the early onset of PCOS. In real-world scenarios, not all obese adolescent girls are categorized as PCOS-suspect cases. Studies have shown higher genetic predisposition to obesity correlates with increased infant fat storage, particularly in visceral regions (31). This can manifest as early-life central obesity, insulin resistance, and hypertension (32, 33), key risk factors for PCOS (34). Without effective health management during growth stages, continuous ectopic fat deposition undermines homeostatic responses and accelerates sexual maturation (35, 36), leading to earlier menarche and inhibiting linear height growth. Ectopic fat triggers reversible endocrine-metabolic disorders, resulting in typical PCOS symptoms. With socioeconomic advancements and improved living conditions, higher rates of improper infant feeding practices have contributed to a rise in childhood obesity (37), potentially associated with increasing PCOS incidences among females aged 10–14. Yet, overlap between adolescent PCOS manifestations and physiological changes within puberty—especially the first two years post-menarche—has led some physicians to refrain from diagnosing PCOS to avoid psychological stress for the patient and family (38). Furthermore, adolescents with poor self-control often face challenges related to unhealthy eating habits, lack of exercise, and stress, exacerbating metabolic and endocrine issues (39, 40) and accelerating the disease burden among this demographic.

### 4.3 Clinical implications

The significant increase in PCOS burden among adolescents and young women over the next 15 years underscores the need for early interventions and clinical follow-ups to mitigate potential long-term adverse effects. Early intervention is essential in slowing PCOS progression and minimizing its psychosocial impact. Due to societal beauty standards and the emphasis on clear skin, visible PCOS symptoms such as skin issues, hirsutism, and obesity may contribute to psychological distress, particularly in adolescents (41, 42). Research has shown that PCOS leads to severe emotional stress, affecting self-esteem, social interaction, and quality of life (43). This distress may exacerbate bullying, anxiety, depression, and, in severe cases, suicidal tendencies among adolescents (44).

Therefore, educating parents and teachers is paramount to guide timely medical consultations, thereby promoting early PCOS detection and appropriate management. Healthcare providers should adopt a holistic approach to address both the physical and emotional aspects of PCOS, offering guidance on healthy weight management and skin care to alleviate the burden for adolescents with PCOS.

### 4.4 Research implications

The findings emphasize the need for further investigations into PCOS pathogenesis and internal/external risk factors, particularly on how BMI increase, childhood obesity, stress levels, and pollutant exposure exacerbate the condition. Developing multidimensional data integration models to refine regional disparities in disease burden remains crucial. Similarly, age-specific physiological and metabolic changes during puberty warrant deeper analysis to identify specific risk factors for this stage.

This study underscores the necessity to establish a harmonized, multinational database to improve PCOS trend evaluations, transcending limitations associated with inconsistent diagnostic criteria and cultural/policy diversity (29, 30). Moreover, longitudinal studies tracking PCOS trajectories from adolescence to reproductive age could provide valuable insights for tailored interventions and long-term monitoring.

### 4.5 Strengths and limitations

This study is the first to assess the PCOS burden among 10–24-year-old females across 204 countries, filling a critical data gap in understanding PCOS during adolescence and early adulthood. However, limitations include the variability in data source quality and biases due to disparate public health monitoring systems, cultural norms, and policies across countries. First, under recognition of PCOS and resource scarcity in certain regions (particularly low-SDI areas) may lead to underreporting or misclassification. Globally, the temporal evolution of diagnostic criteria and inconsistent application of guidelines—such as the NIH 1990 criteria, Rotterdam 2003 criteria, and AES 2006 criteria—further complicate prevalence estimation and introduce bias. Second, while PCOS manifests with diverse phenotypes (e.g., hyperandrogenic, non-hyperandrogenic, and obesity/metabolic subtypes) and risk factors (e.g., diet, obesity, endocrine-disrupting chemicals, and lifestyle), the GBD database lacks phenotypic stratification and risk factor analysis, potentially limiting the interpretation of PCOS burden in this age group. Third, projections for 2022–2036 rely on statistical models that omit potential confounding variables (e.g., environmental changes, advances in healthcare systems), which may bias predictions. Additionally, as this analysis was based on GBD public data, it identifies associations between SDI and PCOS burden but lacks longitudinal individual-level data to establish causality; findings should thus be interpreted cautiously.

To address these challenges, the establishment of standardized data collection frameworks in low-SDI countries and promoting international collaborations for improved data quality is crucial. In middle- and high-SDI countries, public health education and healthcare worker training could enhance PCOS recognition and diagnosis, while longitudinal follow-up of individual patients and phenotypic stratification of PCOS cases would improve data accuracy. Additionally, differentiating phenotypes among PCOS patients during data collection, establishing personalized diagnostic and treatment strategies, and clarifying disease progression and treatment outcomes are critical. The development of adolescent-specific diagnostic criteria and the use of multicriteria sensitivity analyses could further enhance research accuracy.

Despite methodological limitations, this study reveals changes in the global and regional burden of PCOS from 1990 to 2021, providing a basis for optimizing prevention policies and early interventions. Future efforts should prioritize exploring the broader public health and policy implications of this rising burden, establishing long-term follow-up strategies, and clarifying PCOS progression and treatment outcomes.

## 5 Conclusion

In summary, since 1990, the burden of polycystic ovary syndrome (PCOS) among females aged 10 to 24 has exhibited significant regional, age, and diagnostic standard-specific variations globally, further complicating the trend of its prevalence. Managing this widespread and profoundly impactful endocrine disorder remains a major challenge, requiring more precise diagnostic standards and effective interventions to mitigate its health and socioeconomic burdens. Addressing the regional and age-specific disparities in PCOS prevalence demands an integrated approach that combines multidisciplinary healthcare, psychosocial support, and public health policies. Future research should focus on exploring the potential risk factors and pathophysiological mechanisms of PCOS while striving to develop affordable and targeted therapeutic and preventive strategies to tackle the global health challenge posed by the disorder.

## Data Availability

The datasets presented in this study can be found in online repositories. The names of the repository/repositories and accession number(s) can be found in the article/**Supplementary Material**.
