# Supplementary material for: Evolving global trends in PCOS burden: a three-decade analysis (1990–2021) with projections to 2036 among adolescents and young adults
Source: Front Endocrinol (Lausanne). 2025 May 12;16:1569694. doi: 10.3389/fendo.2025.1569694 (PMC12104063; doi:10.3389/fendo.2025.1569694)
Supplement: Supplementary file 1 [file DataSheet1.pdf]

Table S1. prevalence and AAPC of PCOS in adolescents and young adults aged 10-24 years at global and regional level, 1990-2021

	Cases (n), 1990	prevalence (per 100 000 population), 1990	Cases (n), 2021	prevalence (per 100 000 population), 2021	AAPC(95% CI)	*P* value
**Global**	12431336 (8626816,17498544)	1633.33 (1133.46,2299.1)	19709918 (13796725,27652540)	2141.62 (1499.11,3004.64)	0.875(0.823, 0.928)	<0.001
**SDI level**						
High SDI	3768684 (2675963,5238380)	3951.4 (2805.7,5492.35)	4216132 (3081581,5758595)	4695.75 (3432.14,6413.69)	0.535(0.469, 0.6)	<0.001
High-middle SDI	2349707 (1644747,3312353)	1687.87 (1181.48,2379.37)	2668088 (1867326,3743161)	2483.8 (1738.35,3484.62)	1.251(1.18,1.322)	<0.001
Low SDI	452785 (303241,673024)	585.21 (391.93,869.87)	1600769 (1083526,2349489)	870.36 (589.13,1277.45)	1.304(1.26,1.347)	<0.001
Low-middle SDI	1618051 (1097482,2335540)	906.73 (615.01,1308.8)	4134612 (2821138,5955474)	1523.82 (1039.73,2194.9)	1.699(1.67,1.728)	<0.001
Middle SDI	4233190 (2907310,5954015)	1567.69 (1076.67,2204.96)	7075603 (4939058,9961540)	2648.26 (1848.59,3728.41)	1.711(1.68,1.742)	<0.001
**GBD super regions**						
Central Europe, Eastern Europe, and Central Asia	128938 (81630,203730)	272.34 (172.42,430.32)	140060 (91746,208148)	393.2 (257.56,584.34)	1.199(1.153, 1.245)	<0.001
High-income	4614298	4632.58 (3278.3,6404.12)	4714395	5076.78	0.266(0.187,	<0.0

	(3265361,6378845)		(3413917,6443751)	(3676.33,6939.06)	0.344)	01
Latin America and Caribbean	1449842 (983767,2058595)	2299.6 (1560.36,3265.15)	2037289 (1394722,2860024)	2861.41 (1958.91,4016.96)	0.712(0.593, 0.832)	<0.001
North Africa and Middle East	1063335 (719382,1533498)	1998.97 (1352.37,2882.84)	2143681 (1461268,3046452)	2728.35 (1859.81,3877.34)	1.047(0.957, 1.137)	<0.001
South Asia	1245289 (854124,1794731)	771.41 (529.1,1111.77)	3665869 (2498276,5234304)	1440.81 (981.9,2057.25)	2.079(1.987, 2.171)	<0.001
Southeast Asia, East Asia, and Oceania	3414319 (2367918,4887061)	1330.41 (922.68,1904.28)	5361179 (3690728,7606803)	2696.22 (1856.12,3825.58)	2.316(2.273, 2.358)	<0.001
Sub-Saharan Africa	515317 (341203,761375)	645.37 (427.32,953.53)	1647444 (1103435,2390413)	872.57 (584.44,1266.09)	0.996(0.917, 1.076)	<0.001
**GBD regions**						
Andean Latin America	199055 (136353,283545)	3216.21 (2203.11,4581.35)	385860 (262340,557081)	4616.31 (3138.56,6664.74)	1.214(1.136, 1.292)	<0.001
Australasia	130812 (94909,176618)	5537.06 (4017.33,7475.94)	175161 (125068,241368)	6282.07 (4485.5,8656.54)	0.405(0.373, 0.437)	<0.001
Caribbean	86179 (57287,122375)	1603.21 (1065.73,2276.57)	113122 (75570,162485)	2019.41 (1349.05,2900.62)	0.761(0.704, 0.818)	<0.001
Central Asia	42892 (27362,64937)	436.36 (278.36,660.63)	66217 (43939,96192)	616.23 (408.91,895.18)	1.133(1.027, 1.24)	<0.001
Central Europe	32690 (19630,52138)	228.74 (137.36,364.83)	26398 (17098,38911)	300.13 (194.39,442.39)	0.879(0.868, 0.89)	<0.001
Central Latin America	993587 (679580,1409050)	3618.3 (2474.79,5131.27)	1333355 (914019,1852635)	4127.55 (2829.44,5735.03)	0.429(0.247, 0.613)	<0.001

Central Sub-Saharan Africa	45114 (29642,67823)	523.26 (343.8,786.65)	182638 (120739,270108)	817.12 (540.18,1208.46)	1.47(1.359,1.58)	<0.001
East Asia	1998234 (1365038,2883696)	1101.76 (752.63,1589.97)	2208979 (1521893,3142326)	1947.09 (1341.46,2769.78)	1.841(1.738,1.943)	<0.001
Eastern Europe	53355 (32278,84715)	229.75 (138.99,364.79)	47445 (30107,74884)	295.06 (187.23,465.7)	0.821(0.766,0.876)	<0.001
Eastern Sub-Saharan Africa	191571 (125543,285234)	610.63 (400.17,909.19)	598292 (398753,873224)	820.67 (546.97,1197.79)	0.988(0.933,1.044)	<0.001
High-income Asia Pacific	1188814 (847009,1641401)	5793.95 (4128.09,7999.74)	853375 (608597,1159446)	6725.49 (4796.38,9137.64)	0.473(0.395,0.551)	<0.001
High-income North America	1185291 (831896,1667724)	3964.9 (2782.76,5578.68)	1666580 (1227032,2238899)	4781.08 (3520.1,6422.94)	0.529(0.337,0.722)	<0.001
North Africa and Middle East	1063335 (719382,1533498)	1998.97 (1352.37,2882.84)	2143681 (1461268,3046452)	2728.35 (1859.81,3877.34)	1.047(0.957,1.137)	<0.001
Oceania	16342 (10949,23542)	1619.93 (1085.35,2333.6)	44254 (30217,63414)	2301.46 (1571.44,3297.9)	1.116(1.049,1.183)	<0.001
South Asia	1245289 (854124,1794731)	771.41 (529.1,1111.77)	3665869 (2498276,5234304)	1440.81 (981.9,2057.25)	2.079(1.987,2.171)	<0.001
Southeast Asia	1399742 (972712,1993387)	1884.95 (1309.89,2684.38)	3107946 (2133488,4370294)	3723.54 (2556.07,5235.92)	2.243(2.169,2.317)	<0.001
Southern Latin America	96540 (67078,139270)	1459.41 (1014.03,2105.36)	185139 (129368,264863)	2450.72 (1712.47,3506.03)	1.72(1.666,1.774)	<0.001
Southern Sub-Saharan Africa	94500 (62230,136804)	1080.22 (711.34,1563.79)	147944 (100113,214162)	1364.02 (923.03,1974.55)	0.798(0.653,0.943)	<0.001
Tropical Latin America	171020	711.9 (455.53,1066.44)	204952	821.96 (542.95,1190.19)	0.447(0.341,	<0.0

	(109433,256190)		(135381,296770)		0.552)	01
Western Europe	2012841 (1406079,2813244)	5005.2 (3496.41,6995.51)	1834139 (1291622,2559540)	5244.49 (3693.23,7318.68)	0.148(0.133, 0.163)	<0.001
Western Sub-Saharan Africa	184131 (121364,276465)	591.96 (390.17,888.8)	718570 (479364,1051075)	868.86 (579.62,1270.9)	1.264(1.133, 1.394)	<0.001

Table S2. DALYs and AAPC of PCOS in adolescents and young adults aged 10-24 years at global and regional level, 1990-2021

	Cases (n), 1990	DALYs (per 100 000 population), 1990	Cases (n), 2021	DALYs (per 100 000 population), 2021	AAPC(95% CI)	*P* value
Global	113006 (50849,228343)	14.85 (6.68,30)	178473 (80140,363894)	19.39 (8.71,39.54)	0.862(0.798, 0.926)	<0.001
SDI level						
High SDI	34213 (15575,69512)	35.87 (16.33,72.88)	38279 (17538,77784)	42.63 (19.53,86.63)	0.535(0.454, 0.617)	<0.001
High-middle SDI	21147 (9563,42805)	15.19 (6.87,30.75)	24153 (10872,49162)	22.49 (10.12,45.77)	1.272(1.198, 1.346)	<0.001
Low SDI	4119 (1781,8742)	5.32 (2.3,11.3)	14415 (6260,30615)	7.84 (3.4,16.65)	1.286(1.217, 1.355)	<0.001
Low-middle SDI	15003 (6493,31549)	8.41 (3.64,17.68)	37559 (16526,78679)	13.84 (6.09,29)	1.635(1.6,1.67)	<0.001
Middle SDI	38443 (16965,78288)	14.24 (6.28,28.99)	63933 (28477,131523)	23.93 (10.66,49.23)	1.695(1.663, 1.728)	<0.001
GBD super regions						
Central Europe, Eastern Europe, and Central Asia	1196 (498,2596)	2.53 (1.05,5.48)	1289 (554,2724)	3.62 (1.56,7.65)	1.175(1.12,1.229)	<0.001

High-income	41781 (18868,85870)	41.95 (18.94,86.21)	42850 (19557,87244)	46.14 (21.06,93.95)	0.278(0.185, 0.371)	<0.001
Latin America and Caribbean	12987 (5774,26989)	20.6 (9.16,42.81)	18167 (8056,37642)	25.52 (11.31,52.87)	0.697(0.584, 0.81)	<0.001
North Africa and Middle East	9914 (4334,20509)	18.64 (8.15,38.55)	19609 (8628,40674)	24.96 (10.98,51.77)	0.978(0.857, 1.098)	<0.001
South Asia	11597 (5162,24407)	7.18 (3.2,15.12)	33057 (14338,69201)	12.99 (5.64,27.2)	1.954(1.877, 2.031)	<0.001
Southeast Asia, East Asia, and Oceania	30872 (13397,64060)	12.03 (5.22,24.96)	48664 (21867,100370)	24.47 (11,50.48)	2.332(2.282, 2.382)	<0.001
Sub-Saharan Africa	4659 (1992,9892)	5.84 (2.5,12.39)	14836 (6428,31177)	7.86 (3.4,16.51)	0.985(0.893, 1.078)	<0.001
GBD regions						
Andean Latin America	1749 (762,3769)	28.25 (12.3,60.9)	3399 (1488,7063)	40.66 (17.81,84.5)	1.202(1.134, 1.27)	<0.001
Australasia	1166 (523,2374)	49.34 (22.12,100.47)	1561 (707,3180)	55.97 (25.35,114.06)	0.404(0.362, 0.446)	<0.001
Caribbean	796 (346,1680)	14.8 (6.43,31.24)	1032 (462,2175)	18.43 (8.25,38.83)	0.724(0.666, 0.782)	<0.001
Central Asia	392 (164,836)	3.98 (1.67,8.51)	603 (256,1284)	5.61 (2.38,11.95)	1.123(1.026, 1.221)	<0.001
Central Europe	298 (122,632)	2.09 (0.86,4.42)	239 (103,491)	2.72 (1.17,5.58)	0.857(0.824, 0.89)	<0.001
Central Latin America	8878 (3945,18541)	32.33 (14.37,67.52)	11861 (5319,24583)	36.72 (16.47,76.1)	0.416(0.248, 0.584)	<0.001
Central Sub-Saharan Africa	405 (171,853)	4.7 (1.99,9.9)	1643 (710,3399)	7.35 (3.18,15.21)	1.457(1.31,1	<0.00

					.604)	1
East Asia	17762 (7680,37225)	9.79 (4.23,20.52)	19772 (8861,41053)	17.43 (7.81,36.19)	1.861(1.755, 1.968)	<0.00 1
Eastern Europe	506 (198,1113)	2.18 (0.85,4.79)	448 (182,989)	2.78 (1.13,6.15)	0.81(0.744,0 .876)	<0.00 1
Eastern Sub-Saharan Africa	1726 (727,3661)	5.5 (2.32,11.67)	5354 (2321,11228)	7.34 (3.18,15.4)	0.963(0.903, 1.022)	<0.00 1
High-income Asia Pacific	10629 (4703,21388)	51.8 (22.92,104.24)	7621 (3374,15485)	60.06 (26.59,122.03)	0.47(0.392,0 .547)	<0.00 1
High-income North America	10761 (4814,21584)	36 (16.1,72.2)	15128 (7028,30363)	43.4 (20.16,87.11)	0.55(0.317,0 .783)	<0.00 1
North Africa and Middle East	9914 (4334,20509)	18.64 (8.15,38.55)	19609 (8628,40674)	24.96 (10.98,51.77)	0.978(0.857, 1.098)	<0.00 1
Oceania	146 (65,305)	14.46 (6.46,30.28)	390 (174,830)	20.28 (9.04,43.18)	1.086(1.022, 1.149)	<0.00 1
South Asia	11597 (5162,24407)	7.18 (3.2,15.12)	33057 (14338,69201)	12.99 (5.64,27.2)	1.954(1.877, 2.031)	<0.00 1
Southeast Asia	12964 (5694,26644)	17.46 (7.67,35.88)	28502 (12699,58488)	34.15 (15.21,70.07)	2.197(2.124, 2.271)	<0.00 1
Southern Latin America	887 (397,1814)	13.4 (6,27.43)	1701 (757,3419)	22.51 (10.03,45.25)	1.727(1.665, 1.789)	<0.00 1
Southern Sub-Saharan Africa	863 (372,1876)	9.86 (4.25,21.44)	1340 (582,2800)	12.36 (5.37,25.82)	0.777(0.675, 0.879)	<0.00 1
Tropical Latin America	1565 (664,3275)	6.52 (2.76,13.63)	1874 (814,3841)	7.52 (3.26,15.41)	0.446(0.352, 0.541)	<0.00 1
Western Europe	18339	45.6 (20.34,93.93)	16840	48.15 (21.06,98.18)	0.172(0.13,0	<0.00

	(8179,37773)		(7365,34336)		.214)	1
Western Sub-Saharan Africa	1666 (718,3547)	5.36 (2.31,11.4)	6499 (2791,13706)	7.86 (3.37,16.57)	1.26(1.133,1.387)	<0.001

Table S3. Age-standardized rate and AAPC of PCOS in adolescents and young adults aged 10-24 years at global and regional level, 1990-2021

	Incidence				Prevalence				DALYs			
	Age-standardized rate (per 100 000 population), 1990	Age-standardized rate (per 100 000 population), 2021	AAPC	*P* value	Age-standardized rate (per 100 000 population), 1990	Age-standardized rate (per 100 000 population), 2021	AAPC	*P* value	Age-standardized rate (per 100 000 population), 1990	Age-standardized rate (per 100 000 population), 2021	AAPC	*P* value
Global	49.45 (35.57,68.45)	63.26 (45.41,87.28)	0.8(0.77,0.831)	<0.001	1372.77 (984.64,1891.6)	1757.83 (1253.36,2421.26)	0.81(0.77,0.849)	<0.001	12.08 (5.38,25.21)	15.4 (6.91,32.13)	0.795(0.754,0.835)	<0.001
SDI level												
High SDI	120.81 (88.32,167.22)	144.89 (107.28,196.81)	0.571(0.497,0.644)	<0.001	3007.94 (2186.43,4172.47)	3554.29 (2624.21,4816.07)	0.53(0.497,0.563)	<0.001	26.66 (12.08,55.34)	31.37 (14.37,64)	0.513(0.478,0.548)	<0.001
High-middle SDI	49.47 (35.43,68.6)	72.52 (51.14,101.53)	1.251(1.211,1.291)	<0.001	1262.73 (892.37,1745.92)	1817.62 (1277.35,2529.47)	1.186(1.17,1.202)	<0.001	11.05 (4.96,22.99)	15.91 (7.1,33.31)	1.191(1.169,1.212)	<0.001
Low	20.08	27.79	1.078(1.021,	<0.0	500.06	714.31	1.184(1.101,	<0.0	4.35	6.2	1.177(1.084,	<0.0

SDI	(14.45,28.07)	(19.81,38.87)	1.135)	01	(353.56,711.57)	(504.52,1007.4)	1.266)	01	(1.86,9.22)	(2.67,13.05)	1.271)	01
Low-middle SDI	29.32 (21.09,40.77)	45.2 (31.82,62.95)	1.412(1.376,1.448)	<0.001	745.13 (526.92,1040.82)	1188.74 (828.69,1668.94)	1.525(1.498,1.553)	<0.001	6.59 (2.84,13.85)	10.43 (4.56,21.89)	1.499(1.467,1.531)	<0.001
Middle SDI	48.52 (34.54,67.44)	77.38 (54.44,107.63)	1.524(1.489,1.558)	<0.001	1176.94 (825.22,1630.44)	1971.07 (1395.79,2724.23)	1.687(1.646,1.729)	<0.001	10.32 (4.57,21.76)	17.26 (7.68,36.24)	1.679(1.65,1.709)	<0.001
GBD super regions												
Central Europe, Eastern Europe, and Central Asia	9.31 (6.58,13.19)	12.77 (9.1,17.89)	1.04(1.017,1.063)	<0.001	224.24 (153.91,326.1)	312.73 (216.79,443.56)	1.092(1.063,1.121)	<0.001	1.98 (0.83,4.17)	2.75 (1.17,5.8)	1.08(1.053,1.107)	<0.001
High-income	140.65 (102.33,194.32)	157.39 (116.26,212.41)	0.351(0.294,0.409)	<0.001	3487.86 (2537.97,4764.65)	3946.73 (2914.89,5347.81)	0.387(0.353,0.421)	<0.001	30.92 (14.16,64.2)	34.9 (15.94,71.42)	0.376(0.337,0.416)	<0.001
Latin America and Caribbean	69.4 (48.09,98.87)	82.16 (57.63,115.2)	0.548(0.508,0.588)	<0.001	1676.34 (1150.68,2353.79)	1998.61 (1401.44,2781.11)	0.571(0.494,0.648)	<0.001	14.69 (6.46,30.51)	17.37 (7.66,36.07)	0.544(0.463,0.626)	<0.001

North Africa and Middle East	59.01 (41.15,83.29)	77.15 (54.45,109.17)	0.897(0.827,0.968)	<0.001	1548.77 (1073.96,2200.34)	2075.28 (1453.41,2932.69)	0.971(0.9,1.041)	<0.001	13.96 (6.1,29.29)	18.4 (8.24,39.14)	0.912(0.828,0.995)	<0.001
South Asia	25.33 (18.35,34.97)	42.08 (29.94,58.32)	1.676(1.563,1.791)	<0.001	642.8 (462.57,893.35)	1135.87 (799.7,1591.05)	1.868(1.812,1.923)	<0.001	5.68 (2.49,12.07)	9.94 (4.34,20.79)	1.836(1.801,1.872)	<0.001
Southeast Asia, East Asia, and Oceania	40.39 (28.79,56.47)	80.13 (56.57,111.88)	2.254(2.191,2.318)	<0.001	1026.3 (717.66,1448.48)	2020.68 (1418.6,2842.16)	2.218(2.139,2.297)	<0.001	8.92 (3.87,18.46)	17.66 (7.82,36.61)	2.234(2.136,2.333)	<0.001
Sub-Saharan Africa	21.53 (15.41,30.11)	27.83 (19.82,39.2)	0.85(0.752,0.948)	<0.001	545.32 (384.16,780.33)	727.03 (512.13,1029.5)	0.957(0.91,1.005)	<0.001	4.71 (2,9.96)	6.28 (2.72,13.22)	0.958(0.909,1.006)	<0.001
GBD regions												
Andean Latin America	100.3 (68.88,142.65)	134.14 (92.65,190.7)	0.955(0.914,0.996)	<0.001	2419.72 (1673.59,3379.44)	3333.66 (2301.61,4690.91)	1.065(0.981,1.149)	<0.001	20.98 (9.37,45.26)	28.78 (12.57,60.15)	1.048(0.984,1.113)	<0.001
Austral	173.96	202.25	0.487(0.447,	<0.0	4122.5	4786.95	0.478(0.45,0.	<0.0	35.97	41.77	0.475(0.431,	<0.0

asia	(127.23,229.44)	(145.07,278.86)	0.527)	01	(3027.98,5452.98)	(3430.23,6607.27)	506)	01	(16.34,74.76)	(18.75,86.35)	0.52)	01
Caribbean	44.33 (30.33,62.54)	55.61 (37.61,78.19)	0.74(0.687,0.792)	<0.001	1186.23 (799.13,1702.73)	1485.97 (1008.31,2136.92)	0.73(0.667,0.793)	<0.001	10.5 (4.58,21.73)	13.03 (5.7,27.37)	0.705(0.639,0.771)	<0.001
Central Asia	13.68 (9.46,19.63)	18.79 (13.06,26.29)	1.039(0.999,1.079)	<0.001	348.5 (234.84,511.4)	483.56 (330.71,678.72)	1.07(1.037,1.104)	<0.001	3.06 (1.28,6.52)	4.24 (1.81,9.1)	1.062(1.036,1.088)	<0.001
Central Europe	7.35 (5.01,10.81)	8.97 (6.22,12.67)	0.647(0.635,0.659)	<0.001	185.82 (122.77,278.52)	228.93 (157.78,326.2)	0.676(0.661,0.691)	<0.001	1.63 (0.68,3.36)	2 (0.86,4.17)	0.666(0.653,0.68)	<0.001
Central Latin America	107.23 (73.94,151.71)	115.89 (81.09,162.25)	0.221(0.114,0.328)	<0.001	2695.62 (1856.93,3736.43)	2967.64 (2076.22,4124.04)	0.312(0.168,0.456)	<0.001	23.55 (10.46,49.22)	25.74 (11.36,53.72)	0.289(0.137,0.441)	<0.001
Central Sub-Saharan Africa	17.63 (12.55,24.87)	26.2 (18.46,36.97)	1.314(1.226,1.402)	<0.001	445.38 (309.5,646.83)	673.86 (465.36,968.69)	1.392(1.266,1.519)	<0.001	3.83 (1.63,8)	5.82 (2.51,12.02)	1.379(1.273,1.484)	<0.001
East Asia	32.14 (22.85,44.87)	58.63 (41.2,82.32)	1.975(1.899,2.052)	<0.001	845.57 (600.94,1185.47)	1548.43 (1085.52,2170.68)	1.992(1.914,2.071)	<0.001	7.25 (3.13,15.07)	13.38 (5.91,27.61)	2.018(1.946,2.091)	<0.001
Eastern Europe	8.56 (5.98,12.09)	10.86 (7.64,15.38)	0.783(0.742,0.824)	<0.001	206.51 (139.23,298.06)	264.99 (182.67,381.43)	0.82(0.795,0.844)	<0.001	1.83 (0.76,3.89)	2.35 (0.97,4.92)	0.814(0.788,0.839)	<0.001
Eastern	20.71	26.26	0.781(0.757,	<0.0	516.67	669.17	0.857(0.813,	<0.0	4.46	5.77	0.856(0.811,	<0.0

Sub-Saharan Africa	(14.82,29.05)	(18.57,36.96)	0.804)	01	(364.08,741.26)	(472.35,949.85)	0.901)	01	(1.88,9.4)	(2.49,12.17)	0.902)	01
High-income Asia Pacific	193.02 (137.42,268.99)	225.11 (160.59,317.34)	0.495(0.462,0.528)	<0.001	4750.21 (3430.32,6586.66)	5237.62 (3779.21,7307.15)	0.32(0.298,0.342)	<0.001	41.4 (18.34,83.4)	45.62 (20.81,92.93)	0.316(0.293,0.338)	<0.001
High-income North America	119.24 (83.99,166.82)	149.79 (111.27,197.64)	0.653(0.484,0.822)	<0.001	2975.32 (2098.69,4185.41)	3729.48 (2777.89,4876.97)	0.659(0.415,0.904)	<0.001	26.6 (11.77,54.7)	33.02 (15.2,66.9)	0.646(0.488,0.804)	<0.001
North Africa and Middle East	59.01 (41.15,83.29)	77.15 (54.45,109.17)	0.897(0.827,0.968)	<0.001	1548.77 (1073.96,2200.34)	2075.28 (1453.41,2932.69)	0.971(0.9,1.041)	<0.001	13.96 (6.1,29.29)	18.4 (8.24,39.14)	0.912(0.828,0.995)	<0.001
Oceania	49.56 (34.79,69.76)	69.33 (48.42,97.52)	1.062(1.001,1.123)	<0.001	1278.85 (881.22,1798.41)	1772.65 (1232.67,2531.47)	1.022(0.967,1.077)	<0.001	11.14 (5.06,23.3)	15.41 (6.71,32.47)	1.017(0.965,1.069)	<0.001
South Asia	25.33 (18.35,34.97)	42.08 (29.94,58.32)	1.676(1.563,1.791)	<0.001	642.8 (462.57,893.35)	1135.87 (799.7,1591.05)	1.868(1.812,1.923)	<0.001	5.68 (2.49,12.07)	9.94 (4.34,20.79)	1.836(1.801,1.872)	<0.001
Southeast Asia	58.95 (41.5,82.07)	110.11 (77.87,153.89)	2.065(2.009,2.121)	<0.001	1522.31 (1075.34,2154.4)	2842.65 (1993.15,3997.51)	2.065(1.986,2.144)	<0.001	13.5 (5.92,27.49)	25.06 (11.1,51.68)	2.024(1.96,2.088)	<0.001

Southern Latin America	46.78 (32.37,66.78)	74.53 (52.77,106.57)	1.526(1.465, 1.587)	<0.001	1185.87 (822.02,1727.03)	1892.5 (1326.86,2707.18)	1.531(1.465, 1.597)	<0.001	10.5 (4.71,21.66)	16.74 (7.33,34.66)	1.538(1.469, 1.607)	<0.001
Southern Sub-Saharan Africa	33.79 (23.76,47.64)	42.33 (29.66,59.97)	0.764(0.69,0.839)	<0.001	870.65 (601.45,1246.58)	1094.76 (749.33,1548.05)	0.776(0.697, 0.855)	<0.001	7.59 (3.27,16.39)	9.47 (4.1,19.92)	0.736(0.659, 0.813)	<0.001
Tropical Latin America	23.31 (15.97,33.28)	24.9 (17.12,35.01)	0.203(0.111, 0.295)	<0.001	558.68 (379.28,801.65)	610 (419.25,869.52)	0.268(0.107, 0.429)	0.001	4.97 (2.11,10.47)	5.38 (2.33,11.29)	0.241(0.152, 0.329)	<0.001
Western Europe	143.35 (100.92,200.55)	154.64 (109.19,215.84)	0.242(0.221, 0.264)	<0.001	3547.26 (2498.52,4925.03)	3942.92 (2761.02,5529.65)	0.337(0.315, 0.36)	<0.001	31.64 (14.36,65.86)	35.16 (15.84,72.77)	0.334(0.308, 0.36)	<0.001
Western Sub-Saharan Africa	20.04 (14.4,27.97)	27.8 (19.81,39.19)	1.074(0.971, 1.177)	<0.001	501.95 (354.54,717.07)	722.68 (508.32,1026.25)	1.195(1.087, 1.303)	<0.001	4.32 (1.85,9.1)	6.24 (2.69,13.23)	1.203(1.099, 1.307)	<0.001

Table S4. Global AAPCs incidence, prevalence and DALYs of PCOS

	Incidence		Prevalence		DALYs	
	AAPC(95% CI)	*P* value	AAPC(95% CI)	*P* value	AAPC(95% CI)	*P* value
1990-2000	1.04(0.971,1.109)	<0.001	0.824(0.705,0.943)	<0.001	0.841(0.7,0.982)	<0.001
2001-2010	0.084(0.008,0.159)	0.03	1.069(0.976,1.163)	<0.001	1.079(0.954,1.203)	<0.001
2011-2021	1.314(1.26,1.369)	<0.001	0.737(0.7,0.774)	<0.001	0.674(0.633,0.715)	<0.001
1990-2021	0.837(0.797,0.877)	<0.001	0.875(0.823,0.928)	<0.001	0.862(0.798,0.926)	<0.001

Table S5. Age-specific prevalence, incidence, and DALYs of PCOS in adolescents and young adults aged 10-24 years at global level, 1990-2021

Age group(years)	Case in 1990 (million)	Age-specific rate in 1990 (per 100 000)	Case in 2021 (million)	Age-specific rate in 2021 (per 100 000)	AAPC(95% CI)	*P* value
	Prevalence (95% UI)					
10-14	1163280 (606486,1939157)	444.97 (231.99,741.75)	1900416 (1006274,3100291)	588.5 (311.61,960.06)	0.904(0.843,0.966)	<0.001
15-19	4919456 (3335366,7017528)	1925.13 (1305.23,2746.17)	7663403 (5183310,10971947)	2523.76 (1707,3613.35)	0.88(0.859,0.901)	<0.001
20-24	6348600 (4514512,8839643)	2600.45 (1849.19,3620.8)	10146099 (7311868,13931506)	3453.97 (2489.13,4742.61)	0.927(0.891,0.963)	<0.001
	Incidence (95% UI)					
10-14	688832 (359850,1143474)	263.49 (137.65,437.39)	1125449 (598170,1829495)	348.52 (185.23,566.54)	0.905(0.843,0.967)	<0.001
15-19	699967	273.92 (168.93,435.15)	1056349	347.88 (217.07,540.95)	0.786(0.758,0	<0.00

	(431670,1111990)		(659123,1642603)		.815)	1
20-24	41811 (17488,99990)	17.13 (7.16,40.96)	50237 (20474,124736)	17.1 (6.97,42.46)	-0.01(-0.03,0.01)	0.338
	DALYs (95% UI)					
10-14	10102 (4079,22437)	3.86 (1.56,8.58)	16509 (6691,36570)	5.11 (2.07,11.32)	0.902(0.822,0.981)	<0.001
15-19	44340 (19608,93893)	17.35 (7.67,36.74)	68812 (30360,145847)	22.66 (10,48.03)	0.879(0.857,0.901)	<0.001
20-24	58564 (26050,122606)	23.99 (10.67,50.22)	93151 (41705,193793)	31.71 (14.2,65.97)	0.909(0.871,0.947)	<0.001

Table S6. Prevalence of PCOS in adolescents and young adults aged 10-24 years in 2021 and their AAPCs between 1990-2021 in 204 countries and territories

	Cases (n), 1990	Prevalence (per 100,000 population), 1990	Cases (n), 2021	Prevalence (per 100,000 population), 2021	AAPC (95%CI)	*P* value
Afghanistan	19768 (13220,29040)	1142.69 (764.17,1678.62)	86989 (58529,124321)	1683.06 (1132.42,2405.35)	1.308(1.105,1.512)	<0.001
Albania	897 (534,1415)	185.48 (110.54,292.66)	664 (416,1019)	265.11 (166.04,406.96)	1.178(1.149,1.206)	<0.001
Algeria	82542 (54914,119769)	1971.87 (1311.85,2861.21)	143884 (96685,207812)	2893.46 (1944.29,4179.02)	1.28(1.151,1.409)	<0.001
American Samoa	199 (135,283)	2716.83 (1835.06,3853.09)	236 (159,345)	3492.77 (2357.89,5115.25)	0.785(0.683,0.886)	<0.001
Andorra	231 (158,326)	3993.74 (2726.92,5643.69)	301 (205,424)	4787.15 (3263.42,6743.34)	0.555(0.426,0.	<0.00

					684)	1
Angola	7478 (4871,11175)	469.55 (305.85,701.7)	47292 (30821,70914)	876.88 (571.48,1314.87)	2.063(1.923,2.202)	<0.001
Antigua and Barbuda	142 (93,203)	1627.9 (1071.48,2330.34)	218 (146,313)	2251.29 (1507.72,3237.38)	1.07(1.013,1.127)	<0.001
Argentina	60965 (42223,88108)	1397.77 (968.06,2020.1)	122721 (84923,176945)	2320.18 (1605.56,3345.36)	1.703(1.655,1.751)	<0.001
Armenia	1762 (1138,2700)	411.5 (265.85,630.37)	1576 (1037,2340)	620.6 (408.23,921.29)	1.371(1.239,1.502)	<0.001
Australia	99086 (72372,132519)	5086.47 (3715.13,6802.7)	138192 (98455,190207)	6008.06 (4280.41,8269.47)	0.537(0.508,0.566)	<0.001
Austria	43313 (30224,60059)	5459.59 (3809.72,7570.44)	37086 (25671,52540)	5470.59 (3786.83,7750.27)	-0.001(-0.047,0.046)	0.98
Azerbaijan	4489 (2877,6989)	429.64 (275.35,668.85)	6929 (4483,10214)	666.2 (431,981.95)	1.432(1.335,1.528)	<0.001
Bahamas	846 (556,1243)	2094.44 (1377.37,3078.49)	1232 (810,1809)	2469.74 (1624.17,3625.78)	0.532(0.486,0.578)	<0.001
Bahrain	1789 (1190,2561)	2875.78 (1912.34,4115.82)	4806 (3297,6950)	3353.06 (2300.13,4849.12)	0.524(0.27,0.779)	<0.001
Bangladesh	77411 (50270,119671)	437.36 (284.01,676.11)	191890 (129240,282839)	813.77 (548.08,1199.47)	2.036(1.877,2.195)	<0.001
Barbados	700 (468,1000)	2094.08 (1399.43,2991.71)	698 (465,981)	2487.59 (1657.23,3497.66)	0.558(0.506,0.611)	<0.001
Belarus	2673 (1681,4114)	242.95 (152.79,373.95)	2202 (1375,3460)	319.4 (199.33,501.75)	0.941(0.817,1.065)	<0.001
Belgium	40715	4134.78 (2814.49,5934.86)	45568	4736.49 (3285.48,6660.6)	0.447(0.397,0.	<0.00

	(27714,58440)		(31609,64080)		496)	1
Belize	470 (308,672)	1507.82 (987.65,2155.09)	1554 (1023,2213)	2341.44 (1542.04,3334.78)	1.438(1.354,1.523)	<0.001
Benin	4057 (2686,6075)	548.38 (363.06,821.14)	23508 (15414,34245)	1054.69 (691.54,1536.39)	2.136(2.055,2.216)	<0.001
Bermuda	162 (107,235)	2631.07 (1731.44,3819.01)	127 (85,186)	2793.3 (1861.54,4078.8)	0.205(0.128,0.281)	<0.001
Bhutan	690 (459,1041)	663 (440.66,1000.06)	1299 (865,1880)	1297.28 (864.41,1878.29)	2.185(2.085,2.285)	<0.001
Bolivia (Plurinational State of)	29008 (19286,42193)	2879.27 (1914.25,4188.05)	65954 (43861,94410)	4124.74 (2743.06,5904.4)	1.201(1.104,1.298)	<0.001
Bosnia and Herzegovina	872 (498,1463)	156.95 (89.59,263.39)	677 (412,1077)	256.49 (156.12,408.26)	1.624(1.539,1.709)	<0.001
Botswana	1928 (1269,2787)	821.13 (540.46,1187.3)	4654 (3064,6690)	1431.19 (942.28,2057.37)	1.815(1.654,1.976)	<0.001
Brazil	168188 (107719,251956)	718.51 (460.18,1076.37)	197267 (130270,285562)	823.31 (543.69,1191.81)	0.422(0.315,0.529)	<0.001
Brunei Darussalam	1084 (750,1514)	2946.15 (2037.76,4116.14)	2396 (1728,3328)	4859.32 (3504.71,6750.76)	1.634(1.603,1.665)	<0.001
Bulgaria	1986 (1189,3163)	220.37 (131.96,351.1)	1324 (811,2006)	281.3 (172.43,426.33)	0.778(0.717,0.84)	<0.001
Burkina Faso	7454 (4870,11208)	498.3 (325.59,749.28)	32627 (21546,47429)	862.54 (569.59,1253.85)	1.789(1.69,1.889)	<0.001
Burundi	4323 (2824,6348)	504.47 (329.59,740.74)	11653 (7624,17252)	526.24 (344.32,779.1)	0.142(0.077,0.207)	<0.001
Cabo Verde	346 (227,526)	602.27 (394.78,916.6)	829 (554,1218)	1118.63 (747.21,1643.8)	2.029(1.942,2.	<0.00

					116)	1
Cambodia	22581 (15393,32233)	1370 (933.89,1955.61)	57715 (39595,82400)	2549.97 (1749.41,3640.62)	2.04(1.913,2.168)	<0.001
Cameroon	14172 (8939,21227)	859.59 (542.2,1287.5)	60871 (39224,90454)	1172.49 (755.52,1742.3)	1.008(0.957,1.06)	<0.001
Canada	54039 (36778,78144)	1889.36 (1285.86,2732.13)	74532 (51909,105433)	2379.24 (1657.07,3365.69)	0.739(0.688,0.79)	<0.001
Central African Republic	2774 (1835,4256)	652.09 (431.46,1000.56)	6501 (4258,9638)	706.42 (462.68,1047.29)	0.308(0.215,0.402)	<0.001
Chad	3620 (2339,5547)	385.53 (249.1,590.79)	18031 (11959,26884)	609.47 (404.22,908.71)	1.497(1.354,1.639)	<0.001
Chile	30000 (20755,43451)	1600.97 (1107.64,2318.8)	53207 (36893,75482)	2784.64 (1930.85,3950.44)	1.799(1.742,1.856)	<0.001
China	1906381 (1302031,2759785)	1085.55 (741.42,1571.51)	2112556 (1450762,3013952)	1936.41 (1329.8,2762.65)	1.873(1.751,1.995)	<0.001
Colombia	116098 (77652,168254)	2232.81 (1493.4,3235.87)	182560 (123190,267008)	3128.35 (2110.99,4575.46)	1.141(1.045,1.237)	<0.001
Comoros	609 (398,946)	790.6 (515.79,1227.49)	1099 (727,1596)	1005.42 (665.48,1459.6)	0.795(0.696,0.895)	<0.001
Congo	2836 (1839,4261)	688.7 (446.71,1034.68)	8359 (5510,12258)	993.56 (654.97,1457.04)	1.198(1.135,1.262)	<0.001
Cook Islands	78 (52,112)	2723.74 (1811.01,3925.21)	89 (60,130)	4229.46 (2856.7,6162.12)	1.418(1.372,1.465)	<0.001
Costa Rica	12905 (8721,18780)	2818.43 (1904.68,4101.53)	21292 (14348,30153)	3895.4 (2624.94,5516.49)	1.052(1.013,1.091)	<0.001

Coted'Ivoire	10875 (7066,16640)	566.52 (368.1,866.88)	40203 (26274,59715)	965.97 (631.28,1434.8)	1.763(1.66,1.865)	<0.001
Croatia	1031 (628,1661)	203.75 (124.24,328.41)	914 (565,1393)	283.52 (175.32,432.1)	1.097(1.028,1.167)	<0.001
Cuba	29467 (19449,42274)	1938.33 (1279.39,2780.81)	23127 (15554,33205)	2451.56 (1648.74,3519.82)	0.775(0.633,0.918)	<0.001
Cyprus	2752 (1891,3959)	3011.64 (2068.63,4331.33)	4673 (3204,6799)	4478.12 (3070.85,6515.05)	1.269(1.173,1.364)	<0.001
Czechia	2365 (1374,3729)	203.6 (118.29,321.08)	1982 (1211,3071)	260.46 (159.2,403.6)	0.786(0.633,0.939)	<0.001
Democratic People's Republic of Korea	35482 (24510,51244)	1194.58 (825.2,1725.28)	35568 (24316,49986)	1332.57 (911.03,1872.77)	0.35(0.312,0.389)	<0.001
Democratic Republic of the Congo	30402 (19898,45915)	509.46 (333.44,769.43)	113474 (75226,169090)	773.55 (512.81,1152.68)	1.422(1.261,1.584)	<0.001
Denmark	18518 (12556,26356)	3473.21 (2354.94,4943.25)	22592 (15619,31764)	4413.05 (3050.98,6204.74)	0.778(0.738,0.819)	<0.001
Djibouti	441 (292,674)	634.53 (420.1,969.76)	1802 (1180,2673)	1137.14 (744.57,1687.39)	1.868(1.728,2.009)	<0.001
Dominica	174 (115,257)	1582.85 (1044.3,2332.9)	178 (116,253)	2225.41 (1455.38,3171.76)	1.108(1.044,1.172)	<0.001
Dominican Republic	16827 (11058,24462)	1369.72 (900.16,1991.22)	32274 (21293,46710)	2278.29 (1503.12,3297.36)	1.664(1.624,1.705)	<0.001
Ecuador	61925 (42824,87962)	3766.94 (2604.99,5350.8)	122990 (85481,173376)	5127.63 (3563.84,7228.3)	1.008(0.945,1.071)	<0.001
Egypt	198329 (131321,285110)	2391.11 (1583.24,3437.36)	442911 (301272,640188)	3090.88 (2102.45,4467.58)	0.869(0.822,0.917)	<0.001

El Salvador	19987 (13374,29701)	2211.43 (1479.72,3286.16)	29191 (19541,42467)	3351.07 (2243.25,4875.1)	1.368(1.308,1.428)	<0.001
Equatorial Guinea	372 (242,578)	553.71 (360.3,860.55)	3243 (2152,4684)	1417.57 (940.83,2047.82)	3.084(2.867,3.302)	<0.001
Eritrea	2449 (1588,3762)	446.93 (289.92,686.72)	7249 (4805,10975)	722.16 (478.62,1093.33)	1.621(1.477,1.765)	<0.001
Estonia	410 (252,642)	258.8 (158.76,405.45)	361 (226,579)	366.4 (229.13,588.36)	1.123(0.998,1.247)	<0.001
Eswatini	1611 (1055,2318)	1120.27 (733.75,1611.97)	2666 (1774,3808)	1472.6 (980.27,2103.49)	0.94(0.806,1.075)	<0.001
Ethiopia	33770 (21925,51252)	421.23 (273.49,639.29)	131452 (87591,195673)	708.07 (471.81,1053.99)	1.701(1.676,1.726)	<0.001
Fiji	2437 (1642,3541)	2086.14 (1405.55,3031.67)	3781 (2545,5472)	3226.06 (2171.56,4669.36)	1.407(1.341,1.472)	<0.001
Finland	16289 (11152,23286)	3419.78 (2341.27,4888.57)	19515 (13221,27797)	4366.26 (2958.15,6219.39)	0.784(0.707,0.862)	<0.001
France	215164 (144408,308060)	3469.6 (2328.63,4967.59)	252635 (172469,360452)	4226.15 (2885.11,6029.74)	0.637(0.614,0.66)	<0.001
Gabon	1253 (813,1844)	796.17 (516.54,1171.4)	3769 (2425,5707)	1262.54 (812.51,1911.96)	1.501(1.4,1.603)	<0.001
Gambia	905 (587,1343)	565.02 (366.47,838.15)	3692 (2454,5578)	889.37 (591.19,1343.58)	1.489(1.364,1.614)	<0.001
Georgia	3433 (2160,5137)	541.92 (340.98,810.86)	2791 (1851,3980)	953.68 (632.41,1359.8)	1.885(1.682,2.087)	<0.001
Germany	257820 (178005,357957)	3555.74 (2454.96,4936.78)	249134 (173253,353552)	4168.5 (2898.86,5915.61)	0.509(0.459,0.559)	<0.001

Ghana	13822 (8977,20772)	585.65 (380.37,880.14)	49285 (32770,72388)	938.69 (624.13,1378.71)	1.535(1.39,1.679)	<0.001
Greece	44427 (30600,63830)	3869.44 (2665.15,5559.47)	36259 (24990,51202)	4785.19 (3298.08,6757.38)	0.657(0.609,0.705)	<0.001
Greenland	114 (79,163)	1742.35 (1206.61,2477.56)	117 (82,164)	2111.37 (1490.91,2969.39)	0.608(0.522,0.693)	<0.001
Grenada	169 (110,246)	1306.52 (848.5,1899.18)	250 (167,353)	2074.51 (1386.86,2928.19)	1.519(1.403,1.636)	<0.001
Guam	546 (365,795)	3053.56 (2044.51,4451.04)	727 (493,1036)	4206.11 (2851.82,5995.44)	1.025(0.978,1.072)	<0.001
Guatemala	24304 (16252,34951)	1770.93 (1184.23,2546.73)	72106 (47291,104359)	2893.6 (1897.79,4187.91)	1.577(1.467,1.687)	<0.001
Guinea	4273 (2788,6430)	498.84 (325.45,750.64)	17648 (11731,25641)	794.69 (528.25,1154.6)	1.531(1.42,1.642)	<0.001
Guinea-Bissau	818 (536,1269)	494.58 (323.77,766.81)	2684 (1771,4058)	794.17 (524.2,1200.75)	1.539(1.433,1.644)	<0.001
Guyana	1854 (1229,2759)	1410.66 (935.14,2098.68)	2228 (1463,3147)	2150.99 (1413.06,3039.09)	1.434(1.357,1.511)	<0.001
Haiti	10048 (6485,14946)	999.83 (645.28,1487.13)	24157 (16143,35033)	1290.2 (862.18,1871.11)	0.856(0.76,0.953)	<0.001
Honduras	13575 (9082,19968)	1755.21 (1174.3,2581.78)	46332 (31173,68141)	2902.29 (1952.72,4268.41)	1.636(1.612,1.659)	<0.001
Hungary	2464 (1467,3881)	219.16 (130.43,345.14)	2019 (1287,3128)	286.09 (182.39,443.17)	0.86(0.778,0.942)	<0.001
Iceland	1237 (859,1741)	3990.22 (2770.24,5614.02)	1639 (1120,2320)	4917.65 (3360.26,6961.94)	0.663(0.55,0.777)	<0.001

India	1024739 (699744,1474525)	828.45 (565.71,1192.08)	3100776 (2125454,4440239)	1623.66 (1112.95,2325.04)	2.228(2.143,2.314)	<0.001
Indonesia	559301 (386438,794979)	1873.81 (1294.67,2663.4)	1283532 (886215,1820463)	3826.78 (2642.2,5427.61)	2.344(2.291,2.397)	<0.001
Iran (Islamic Republic of)	190097 (128765,272720)	2096.82 (1420.32,3008.18)	251317 (169159,359006)	2907.36 (1956.91,4153.16)	1.061(0.743,1.38)	<0.001
Iraq	67537 (45650,100284)	2292.56 (1549.6,3404.16)	165287 (109540,241482)	2729.69 (1809.03,3988.04)	0.597(0.469,0.724)	<0.001
Ireland	16802 (11408,24241)	3535.36 (2400.44,5100.6)	21319 (14677,30240)	4420.56 (3043.34,6270.46)	0.729(0.693,0.765)	<0.001
Israel	20355 (13924,29627)	3062.7 (2095.04,4457.8)	44222 (30125,62753)	4018.74 (2737.64,5702.78)	0.872(0.834,0.91)	<0.001
Italy	752018 (530655,1048850)	12258.02 (8649.76,17096.43)	467091 (329117,662055)	11139.35 (7848.9,15788.9)	-0.309(-0.368,-0.249)	<0.001
Jamaica	5945 (3952,8603)	1543.19 (1025.85,2233.35)	7700 (5055,11010)	2208.94 (1450.06,3158.59)	1.178(1.105,1.25)	<0.001
Japan	1015433 (720633,1396740)	7400.81 (5252.21,10179.91)	682730 (488652,925406)	7978.35 (5710.36,10814.25)	0.227(0.131,0.323)	<0.001
Jordan	13869 (9362,20169)	2170.43 (1465.13,3156.35)	50356 (33807,71815)	2799.71 (1879.62,3992.76)	0.856(0.774,0.938)	<0.001
Kazakhstan	9947 (6410,15120)	463.07 (298.41,703.91)	13065 (8460,19066)	647.36 (419.15,944.66)	1.107(1.007,1.208)	<0.001
Kenya	27610 (18399,40777)	685.19 (456.59,1011.94)	78200 (52332,115016)	905.95 (606.26,1332.47)	0.941(0.714,1.169)	<0.001
Kiribati	202 (136,287)	1825.58 (1230.45,2589.21)	461 (312,657)	2608.62 (1764.81,3715.97)	1.143(1.05,1.2	<0.00

					35)	1
Kuwait	6973 (4780,10110)	3092.44 (2119.8,4483.5)	15359 (10487,21973)	3786.58 (2585.39,5417.05)	0.664(0.601,0.727)	<0.001
Kyrgyzstan	2692 (1693,4057)	420.14 (264.29,633.28)	4432 (2818,6657)	508.03 (323.06,763.13)	0.616(0.557,0.674)	<0.001
Lao People's Democratic Republic	10246 (6987,14459)	1549.02 (1056.38,2186.05)	32955 (23044,47365)	3189.31 (2230.14,4583.87)	2.365(2.328,2.403)	<0.001
Latvia	694 (435,1125)	263.5 (165.15,427.11)	440 (284,684)	333.74 (215.45,518.12)	0.733(0.579,0.887)	<0.001
Lebanon	10197 (6870,14800)	2402.45 (1618.58,3486.73)	17610 (11994,25638)	3153.63 (2148.04,4591.48)	0.88(0.776,0.984)	<0.001
Lesotho	1879 (1239,2780)	741.25 (488.67,1096.94)	3789 (2526,5596)	1239.32 (826.03,1830.18)	1.667(1.555,1.779)	<0.001
Liberia	2178 (1415,3339)	587.7 (381.75,901.22)	7964 (5248,11787)	888.41 (585.46,1314.88)	1.381(1.226,1.535)	<0.001
Libya	18789 (12532,27422)	2579.37 (1720.39,3764.55)	26689 (18232,38435)	3122.27 (2132.92,4496.39)	0.639(0.538,0.74)	<0.001
Lithuania	952 (571,1527)	237.47 (142.24,380.73)	687 (440,1099)	341.86 (218.94,547.09)	1.19(1.086,1.295)	<0.001
Luxembourg	1422 (966,2020)	4003.95 (2720.52,5688.23)	2547 (1749,3628)	4929.45 (3385.6,7021.09)	0.679(0.61,0.749)	<0.001
Madagascar	11022 (7125,16720)	570.49 (368.8,865.4)	33327 (21787,49532)	683.5 (446.83,1015.84)	0.629(0.563,0.694)	<0.001
Malawi	13030 (8437,19750)	809.03 (523.88,1226.31)	32465 (21277,46737)	894.27 (586.09,1287.4)	0.335(0.249,0.421)	<0.001
Malaysia	79485	2982.91 (2066.36,4372.29)	219241	5581.66 (3787.99,7878.67)	2.051(1.922,2.	<0.00

	(55062,116508)		(148788,309465)		181)	1
Maldives	620 (424,896)	1731.3 (1183.04,2501.81)	1906 (1278,2789)	4249.36 (2848.67,6218.7)	2.932(2.722,3.142)	<0.001
Mali	5427 (3559,8345)	418.21 (274.3,643.09)	28395 (18506,42631)	708.21 (461.57,1063.28)	1.728(1.635,1.821)	<0.001
Malta	1341 (913,1899)	3380.65 (2301.97,4789.03)	1507 (1031,2143)	4879.13 (3337.54,6941.76)	1.205(1.151,1.259)	<0.001
Marshall Islands	103 (70,150)	1379.13 (932.91,2003.58)	211 (144,302)	2581.67 (1767.52,3694.75)	2.038(1.99,2.086)	<0.001
Mauritania	2363 (1562,3631)	733.31 (484.87,1126.96)	7750 (5113,11451)	1057.61 (697.74,1562.71)	1.198(1.11,1.286)	<0.001
Mauritius	4724 (3245,6896)	2954.43 (2029.14,4312.86)	6681 (4624,9570)	5217.44 (3611.29,7474.43)	1.867(1.779,1.956)	<0.001
Mexico	705231 (476622,1001215)	4789.1 (3236.66,6799.08)	834432 (578601,1151855)	5056.93 (3506.51,6980.61)	0.149(-0.001,0.3)	0.052
Micronesia (Federated States of)	308 (207,445)	1823.72 (1224.95,2631.22)	439 (294,634)	2883.22 (1935.78,4169.84)	1.445(1.375,1.515)	<0.001
Monaco	96 (65,136)	4524.38 (3067.93,6384.75)	126 (86,179)	4908.37 (3362.06,6968.85)	0.255(0.196,0.313)	<0.001
Mongolia	1394 (862,2076)	396.25 (245.18,590.32)	2140 (1384,3189)	572.48 (370.33,853.24)	1.208(1.122,1.294)	<0.001
Montenegro	167 (103,264)	217.71 (134.66,344.74)	163 (101,249)	289.38 (179.36,442.74)	0.937(0.885,0.99)	<0.001
Morocco	80946 (53926,114390)	1991.89 (1327,2814.87)	127990 (85936,186731)	2791.52 (1874.29,4072.68)	1.121(0.978,1.264)	<0.001
Mozambique	11937	547.57 (359.4,840.41)	48727	884.85 (581.77,1320.44)	1.579(1.504,1.	<0.00

	(7835,18320)		(32037,72714)		655)	1
Myanmar	98819 (68152,141863)	1496.46 (1032.06,2148.29)	248274 (173013,354282)	3254.11 (2267.67,4643.55)	2.573(2.486,2.66)	<0.001
Namibia	1956 (1321,2802)	811.4 (548.01,1162.26)	4271 (2880,6113)	1136 (766.05,1626)	1.109(1.044,1.175)	<0.001
Nauru	34 (23,48)	2237 (1508.35,3195.7)	55 (38,80)	3318.1 (2272.22,4805.9)	1.254(1.182,1.325)	<0.001
Nepal	13563 (9044,20745)	451.15 (300.84,690.04)	43590 (28914,63478)	907.53 (601.97,1321.59)	2.286(2.162,2.411)	<0.001
Netherlands	56735 (38287,79315)	3540.19 (2389.05,4949.16)	63008 (43359,89315)	4282.53 (2947.02,6070.54)	0.604(0.573,0.635)	<0.001
New Zealand	31726 (21934,45095)	7654.89 (5292.28,10880.44)	36969 (26062,50905)	7573.17 (5338.79,10428.16)	-0.043(-0.091,0.005)	0.082
Nicaragua	13499 (8878,20119)	2017.83 (1327.11,3007.26)	28307 (18868,41075)	3072.74 (2048.16,4458.71)	1.362(1.326,1.398)	<0.001
Niger	5496 (3552,8194)	431.57 (278.95,643.43)	26158 (17463,39023)	612.31 (408.77,913.45)	1.142(1.085,1.199)	<0.001
Nigeria	93630 (61741,141875)	624.57 (411.85,946.39)	352366 (235537,512975)	862.54 (576.56,1255.69)	1.064(0.942,1.185)	<0.001
Niue	7 (5,10)	2378.38 (1568.36,3496.17)	7 (5,10)	3865.73 (2649.36,5568.55)	1.608(1.347,1.87)	<0.001
North Macedonia	446 (261,733)	178.61 (104.62,293.97)	473 (297,747)	265.68 (166.5,419.42)	1.303(1.27,1.336)	<0.001
Northern Mariana Islands	209 (142,299)	3070.5 (2079.9,4391.33)	197 (133,285)	3564.35 (2417.2,5160.56)	0.463(-0.159,1.088)	0.145
Norway	17583	3929.34 (2734.91,5577.44)	20538	4289.23 (2959.08,6143.05)	0.274(0.179,0.	<0.00

	(12238,24958)		(14169,29415)		369)	1
Oman	4214 (2753,6271)	1729.07 (1129.68,2573.06)	13588 (9278,19481)	3135.21 (2140.83,4494.98)	1.976(1.877,2.075)	<0.001
Pakistan	128885 (85157,191704)	761.45 (503.11,1132.59)	328315 (220252,477431)	938.76 (629.77,1365.13)	0.725(0.546,0.905)	<0.001
Palau	60 (41,87)	2680.07 (1807.55,3858.57)	61 (42,89)	3750.71 (2582.57,5418.21)	1.062(0.993,1.131)	<0.001
Palestine	6582 (4374,9439)	1985.5 (1319.4,2847.45)	20789 (13901,30043)	2579.52 (1724.93,3727.78)	0.854(0.769,0.939)	<0.001
Panama	7448 (4913,10939)	1991.95 (1313.87,2925.49)	17398 (11779,25555)	3234.97 (2190.24,4751.65)	1.557(1.5,1.615)	<0.001
Papua New Guinea	8855 (5880,12720)	1407.67 (934.79,2022.09)	30852 (20996,44360)	2130.51 (1449.91,3063.31)	1.318(1.221,1.415)	<0.001
Paraguay	2832 (1753,4335)	460.42 (284.96,704.89)	7684 (4922,11420)	788.8 (505.2,1172.22)	1.759(1.674,1.844)	<0.001
Peru	108122 (72601,154932)	3056.25 (2052.19,4379.41)	196916 (132689,291626)	4515.32 (3042.57,6687.04)	1.299(1.179,1.42)	<0.001
Philippines	202069 (137721,288893)	1984.18 (1352.32,2836.74)	569582 (392541,815836)	3595.53 (2477.94,5150.03)	1.965(1.852,2.077)	<0.001
Poland	12981 (7834,20802)	309.56 (186.82,496.06)	10251 (6933,14527)	361.2 (244.29,511.84)	0.485(0.453,0.516)	<0.001
Portugal	38651 (26552,53961)	3144.26 (2160.01,4389.68)	35760 (24484,50525)	4450.08 (3046.79,6287.43)	1.122(1.066,1.179)	<0.001
Puerto Rico	11867 (7837,17090)	2429.82 (1604.61,3499.19)	9874 (6474,14481)	3272.7 (2145.83,4799.92)	0.97(0.931,1.009)	<0.001
Qatar	1132 (762,1636)	2876.76 (1935.65,4156.27)	5805 (3871,8465)	3425.05 (2284.15,4994.7)	0.586(0.472,0.	<0.00

					7)	1
Republic of Korea	159860 (113037,223780)	2511.22 (1775.68,3515.32)	151862 (108078,209919)	4075.87 (2900.74,5634.1)	1.662(1.496,1.828)	<0.001
Republic of Moldova	979 (604,1566)	191.98 (118.31,306.94)	816 (512,1269)	307.65 (192.95,478.18)	1.555(1.441,1.668)	<0.001
Romania	5388 (3222,8619)	185.81 (111.11,297.24)	4028 (2504,6302)	267.3 (166.2,418.26)	1.204(1.097,1.311)	<0.001
Russian Federation	35913 (21676,57159)	232.64 (140.41,370.26)	34698 (21877,54989)	301.81 (190.29,478.29)	0.871(0.721,1.021)	<0.001
Rwanda	7759 (5180,11572)	670.98 (447.94,1000.75)	19969 (13293,29504)	917.13 (610.53,1355.06)	1.002(0.961,1.043)	<0.001
Saint Kitts and Nevis	114 (76,167)	1808.13 (1210.75,2638.2)	164 (108,235)	2685.33 (1771.67,3853.85)	1.289(1.241,1.337)	<0.001
Saint Lucia	339 (218,487)	1484.2 (954.39,2132.99)	383 (256,550)	2164.55 (1449.14,3113.13)	1.226(1.123,1.329)	<0.001
Saint Vincent and the Grenadines	246 (161,359)	1333.15 (871.53,1947.2)	274 (182,387)	2121.66 (1411.94,3000.55)	1.543(1.503,1.584)	<0.001
Samoa	613 (401,887)	2256.78 (1478.62,3265.65)	940 (635,1363)	3099.04 (2094.83,4493.2)	1.024(0.946,1.103)	<0.001
San Marino	116 (80,162)	4151.97 (2872.45,5830.27)	125 (85,176)	4761.16 (3255.52,6695.39)	0.434(0.391,0.478)	<0.001
Sao Tome and Principe	118 (78,176)	588.91 (387.46,877.11)	338 (221,489)	955.15 (625.02,1382.85)	1.57(1.459,1.682)	<0.001
Saudi Arabia	55519 (37649,80377)	2388.39 (1619.65,3457.77)	136651 (92574,199008)	3727.18 (2524.96,5427.96)	1.468(1.421,1.514)	<0.001
Senegal	8212 (5414,12423)	652.85 (430.43,987.67)	21918	869.26 (577.53,1277.37)	0.938(0.881,0.	<0.00

			(14562,32209)		995)	1
Serbia	1968 (1172,3149)	184.01 (109.56,294.37)	2000 (1234,3099)	261.84 (161.52,405.68)	1.147(1.092,1.203)	<0.001
Seychelles	358 (249,523)	3208.24 (2228.44,4693.49)	481 (333,684)	4532.25 (3131.41,6444.18)	1.12(1.032,1.208)	<0.001
Sierra Leone	3241 (2141,4881)	516.2 (340.88,777.25)	13647 (8880,20899)	919.32 (598.22,1407.87)	1.887(1.721,2.053)	<0.001
Singapore	12437 (8916,17377)	3148.63 (2257.11,4399.21)	16388 (11680,22836)	4600.44 (3278.83,6410.75)	1.262(1.155,1.37)	<0.001
Slovakia	1162 (689,1842)	186.07 (110.42,295.11)	1112 (705,1699)	273.11 (173.16,417.32)	1.247(1.178,1.317)	<0.001
Slovenia	442 (269,694)	203.04 (123.38,318.79)	408 (256,620)	284.4 (178.65,432.81)	1.099(1,1.197)	<0.001
Solomon Islands	811 (539,1170)	1440.67 (957.9,2079.7)	2400 (1634,3383)	2356.5 (1603.93,3321.45)	1.569(1.492,1.646)	<0.001
Somalia	6284 (4099,9518)	536.06 (349.63,811.97)	23519 (15550,35827)	675.85 (446.85,1029.54)	0.744(0.673,0.815)	<0.001
South Africa	70183 (46248,102134)	1161.41 (765.32,1690.14)	105661 (70665,153397)	1496.49 (1000.83,2172.57)	0.833(0.691,0.975)	<0.001
South Sudan	6094 (4002,9332)	644.06 (422.9,986.19)	11810 (7819,18082)	710.01 (470.07,1087.11)	0.333(0.248,0.417)	<0.001
Spain	163888 (111443,229641)	3445.56 (2342.96,4827.94)	145699 (98994,208289)	4274.45 (2904.24,6110.68)	0.703(0.671,0.735)	<0.001
Sri Lanka	61994 (43332,89107)	2409.59 (1684.25,3463.44)	106791 (74316,151969)	4092.05 (2847.68,5823.21)	1.735(1.604,1.867)	<0.001
Sudan	41791	1297.75 (851.11,1897.77)	166988	2402.74 (1624.9,3496.78)	2.008(1.95,2.0	<0.00

	(27408,61113)		(112929,243022)		67)	1
Suriname	945 (631,1359)	1594.57 (1064.8,2293.04)	1510 (1006,2135)	2182.87 (1453.61,3085.36)	1.029(0.998,1.059)	<0.001
Sweden	23810 (16371,34089)	2946.91 (2026.17,4219.09)	28482 (19443,41026)	3333.4 (2275.53,4801.44)	0.407(0.299,0.515)	<0.001
Switzerland	25474 (17402,36184)	3949.38 (2697.87,5609.73)	27638 (18923,39134)	4213.21 (2884.7,5965.69)	0.202(0.161,0.244)	<0.001
Syrian Arab Republic	40937 (27667,58709)	1927.05 (1302.38,2763.67)	68197 (45732,98218)	2831.72 (1898.94,4078.28)	1.287(1.15,1.424)	<0.001
Taiwan (Province of China)	56372 (37390,82340)	2024.78 (1342.98,2957.5)	60856 (42130,83795)	3612.3 (2500.77,4973.96)	1.896(1.771,2.021)	<0.001
Tajikistan	2723 (1733,4218)	328.69 (209.18,509.17)	5992 (3844,9119)	443.52 (284.53,674.97)	0.978(0.925,1.031)	<0.001
Thailand	206046 (143898,296185)	2334.86 (1630.61,3356.28)	278630 (192095,393604)	4786.37 (3299.84,6761.42)	2.356(2.239,2.474)	<0.001
Timor-Leste	1708 (1186,2475)	1567.26 (1087.85,2271.3)	6089 (4270,8801)	2623.69 (1839.98,3792.14)	1.692(1.618,1.766)	<0.001
Togo	3118 (2021,4760)	518.83 (336.22,791.99)	10647 (6975,15796)	834.4 (546.64,1237.84)	1.543(1.406,1.681)	<0.001
Tokelau	4 (3,6)	2043.35 (1388.48,2927.8)	6 (4,8)	3322.95 (2250.82,4825.95)	1.583(1.484,1.683)	<0.001
Tonga	416 (282,596)	2580.55 (1747.79,3699.46)	548 (378,777)	3628.79 (2504.04,5142.66)	1.086(1.03,1.142)	<0.001
Trinidad and Tobago	2623 (1692,3786)	1544.51 (996.45,2229.28)	3142 (2085,4512)	2333.42 (1548.46,3350.13)	1.37(1.249,1.49)	<0.001
Tunisia	25820	1962.69 (1277.6,2875.25)	34577	2827.13 (1900.02,4097.87)	1.234(1.152,1.	<0.00

	(16807,37825)		(23238,50119)		317)	1
Turkey	166242 (109571,242378)	1849.59 (1219.08,2696.67)	255657 (172701,362873)	2724.96 (1840.76,3867.73)	1.205(0.882,1.529)	<0.001
Turkmenistan	2433 (1540,3725)	428.96 (271.48,656.8)	4170 (2724,6227)	654.75 (427.74,977.76)	1.373(1.295,1.451)	<0.001
Tuvalu	24 (17,35)	1984.49 (1348.28,2839.31)	51 (34,74)	3145.78 (2084.49,4517.19)	1.481(1.445,1.517)	<0.001
Uganda	19138 (12501,28764)	659.15 (430.55,990.7)	62908 (42387,91351)	832.86 (561.18,1209.43)	0.779(0.705,0.854)	<0.001
Ukraine	11733 (7201,18605)	219.23 (134.54,347.61)	8240 (5065,13029)	257.77 (158.46,407.58)	0.564(0.397,0.731)	<0.001
United Arab Emirates	4427 (3008,6463)	2415.7 (1641.33,3526.61)	17464 (11920,25926)	3245.98 (2215.59,4818.89)	1.002(0.86,1.143)	<0.001
United Kingdom	252431 (174809,361748)	4335.66 (3002.45,6213.26)	305061 (211031,432945)	5126.56 (3546.38,7275.65)	0.542(0.513,0.57)	<0.001
United Republic of Tanzania	34008 (22015,51447)	769.29 (498,1163.77)	95748 (62166,139192)	966.95 (627.81,1405.7)	0.749(0.719,0.779)	<0.001
United States of America	1131110 (793185,1589855)	4185.08 (2934.77,5882.43)	1591906 (1176071,2128577)	5018.75 (3707.76,6710.69)	0.508(0.313,0.704)	<0.001
United States Virgin Islands	322 (213,466)	2284.95 (1509.58,3306.98)	205 (134,297)	2959.77 (1939.19,4284.33)	0.831(0.794,0.869)	<0.001
Uruguay	5571 (3836,8047)	1468.81 (1011.36,2121.59)	9201 (6431,13189)	2598.67 (1816.24,3725.14)	1.87(1.758,1.981)	<0.001
Uzbekistan	14019 (8871,21085)	439.9 (278.35,661.62)	25121 (16450,36426)	643.07 (421.1,932.47)	1.236(1.171,1.3)	<0.001

Vanuatu	394 (264,565)	1692.29 (1136.98,2428.01)	1206 (826,1756)	2625.89 (1798.76,3823.64)	1.424(1.384,1.464)	<0.001
Venezuela (Bolivarian Republic of)	80539 (54715,118070)	2698.83 (1833.48,3956.47)	101738 (67260,149289)	3388.6 (2240.23,4972.41)	0.715(0.618,0.812)	<0.001
Viet Nam	149766 (103399,211885)	1383.17 (954.95,1956.87)	291734 (198432,411036)	2841.12 (1932.47,4002.97)	2.362(2.282,2.441)	<0.001
Yemen	25253 (16737,37236)	1244.75 (824.98,1835.44)	88767 (58319,131508)	1642.32 (1079,2433.09)	0.917(0.836,0.997)	<0.001
Zambia	12961 (8311,19675)	905.87 (580.86,1375.12)	37843 (24419,57134)	1123.77 (725.13,1696.62)	0.695(0.612,0.778)	<0.001
Zimbabwe	16943 (11040,24333)	924.76 (602.57,1328.08)	26902 (18078,38427)	1035.67 (695.96,1479.37)	0.378(0.297,0.459)	<0.001

Figure S7. DALYs of PCOS in adolescents and young adults aged 10-24 years in 2021 and their AAPCs between 1990-2021 in 204 countries and territories

	Cases (n), 1990	DALYs (per 100 000 population), 1990	Cases (n), 2021	DALYs (per 100 000 population), 2021	AAPC (95%CI)	*P* value
Afghanistan	173 (76,378)	9.98 (4.38,21.87)	778 (336,1666)	15.05 (6.49,32.23)	1.39(1.187,1.593)	<0.001
Albania	8 (3,19)	1.7 (0.68,3.86)	6 (2,14)	2.44 (0.96,5.51)	1.203(1.063,1.343)	<0.001
Algeria	753 (313,1530)	17.98 (7.47,36.55)	1302 (566,2646)	26.18 (11.38,53.2)	1.251(1.095,1.409)	<0.001
American Samoa	2 (1,4)	24.45 (10.67,50.39)	2 (1,4)	31.28 (13.7,65.76)	0.773(0.685,0.861)	<0.001

Andorra	2 (1,4)	37.02 (16.46,76.31)	3 (1,6)	44.04 (19.57,89.57)	0.53(0.396,0.663)	<0.001
Angola	67 (29,140)	4.23 (1.83,8.77)	426 (178,886)	7.9 (3.29,16.43)	2.082(1.886,2.279)	<0.001
Antigua and Barbuda	1 (1,3)	15.06 (6.53,30.55)	2 (1,4)	20.79 (8.88,42.52)	1.06(1,1.12)	<0.001
Argentina	559 (246,1166)	12.82 (5.63,26.73)	1129 (506,2284)	21.35 (9.57,43.18)	1.695(1.61,1.78)	<0.001
Armenia	16 (6,33)	3.7 (1.44,7.68)	14 (6,30)	5.5 (2.34,11.66)	1.319(1.105,1.533)	<0.001
Australia	884 (392,1809)	45.39 (20.11,92.87)	1231 (559,2531)	53.51 (24.3,110.04)	0.53(0.495,0.564)	<0.001
Austria	400 (182,820)	50.46 (22.95,103.39)	342 (149,688)	50.42 (21.94,101.47)	-0.013(-0.071,0.045)	0.66
Azerbaijan	42 (16,92)	3.99 (1.57,8.78)	64 (26,141)	6.15 (2.49,13.58)	1.409(1.259,1.559)	<0.001
Bahamas	8 (3,17)	19.35 (8.25,41.65)	11 (5,25)	22.78 (10.13,49.45)	0.527(0.481,0.572)	<0.001
Bahrain	16 (7,35)	26.25 (11.07,56.51)	44 (19,89)	30.55 (13.57,62.3)	0.508(0.251,0.764)	<0.001
Bangladesh	729 (313,1531)	4.12 (1.77,8.65)	1789 (741,3776)	7.59 (3.14,16.02)	1.993(1.885,2.102)	<0.001
Barbados	6 (3,13)	19.34 (8.45,40.3)	6 (3,14)	22.93 (10.21,49.27)	0.554(0.474,0.634)	<0.001
Belarus	25 (10,53)	2.29 (0.88,4.8)	21 (8,47)	2.99 (1.22,6.76)	0.913(0.76,1.067)	<0.001

Belgium	381 (168,780)	38.7 (17.03,79.26)	423 (190,888)	43.93 (19.72,92.27)	0.42(0.345,0.495)	<0.001
Belize	4 (2,9)	14.1 (6.21,29.43)	14 (6,31)	21.71 (9.54,46.74)	1.414(1.307,1.52)	<0.001
Benin	36 (15,75)	4.87 (2.06,10.2)	211 (90,446)	9.48 (4.05,20)	2.178(2.053,2.303)	<0.001
Bermuda	1 (1,3)	24.3 (10.31,51.58)	1 (1,2)	25.74 (11.26,54.61)	0.199(0.122,0.276)	<0.001
Bhutan	6 (3,13)	6.02 (2.54,12.9)	12 (5,25)	11.77 (5.15,24.5)	2.182(2.065,2.3)	<0.001
Bolivia (Plurinational State of)	256 (108,547)	25.39 (10.68,54.25)	581 (255,1286)	36.31 (15.93,80.4)	1.192(1.053,1.331)	<0.001
Bosnia and Herzegovina	8 (3,18)	1.43 (0.55,3.21)	6 (2,14)	2.32 (0.93,5.3)	1.592(1.46,1.723)	<0.001
Botswana	17 (7,36)	7.23 (3.13,15.26)	41 (18,84)	12.67 (5.62,25.9)	1.812(1.625,1.998)	<0.001
Brazil	1540 (652,3217)	6.58 (2.79,13.74)	1804 (785,3693)	7.53 (3.27,15.41)	0.422(0.326,0.519)	<0.001
Brunei Darussalam	10 (4,20)	26.51 (11.99,54.57)	21 (10,46)	43.53 (19.8,92.77)	1.614(1.578,1.649)	<0.001
Bulgaria	18 (7,43)	2 (0.79,4.82)	12 (5,25)	2.53 (1.02,5.37)	0.778(0.663,0.894)	<0.001
Burkina Faso	70 (29,158)	4.67 (1.94,10.56)	294 (121,635)	7.76 (3.19,16.79)	1.636(1.522,1.75)	<0.001
Burundi	39 (16,84)	4.56 (1.91,9.79)	103 (42,215)	4.67 (1.91,9.69)	0.06(-0.082,0.201)	0.409

Cabo Verde	3 (1,7)	5.42 (2.34,11.46)	7 (3,16)	10.01 (4.25,21.05)	2.004(1.904,2.103)	<0.001
Cambodia	205 (90,417)	12.44 (5.46,25.32)	523 (235,1074)	23.09 (10.38,47.46)	2.016(1.841,2.191)	<0.001
Cameroon	129 (54,273)	7.85 (3.3,16.53)	559 (240,1172)	10.77 (4.61,22.58)	1.038(0.965,1.11)	<0.001
Canada	496 (219,1014)	17.33 (7.67,35.44)	681 (302,1416)	21.74 (9.64,45.21)	0.73(0.664,0.797)	<0.001
Central African Republic	26 (11,56)	6 (2.57,13.23)	60 (25,127)	6.47 (2.74,13.84)	0.265(0.155,0.375)	<0.001
Chad	32 (13,67)	3.4 (1.4,7.15)	160 (67,342)	5.41 (2.25,11.56)	1.518(1.379,1.657)	<0.001
Chile	276 (123,569)	14.73 (6.59,30.38)	487 (213,966)	25.49 (11.17,50.56)	1.788(1.695,1.882)	<0.001
China	16938 (7318,35476)	9.64 (4.17,20.2)	18909 (8479,39246)	17.33 (7.77,35.97)	1.895(1.769,2.02)	<0.001
Colombia	1054 (457,2251)	20.28 (8.79,43.29)	1653 (701,3583)	28.32 (12.01,61.39)	1.13(1.041,1.219)	<0.001
Comoros	6 (2,12)	7.32 (3.09,15.96)	10 (4,21)	9.35 (3.97,18.82)	0.81(0.684,0.936)	<0.001
Congo	25 (10,52)	6.14 (2.5,12.55)	74 (32,153)	8.8 (3.82,18.24)	1.18(1.099,1.261)	<0.001
Cook Islands	1 (0,1)	24.43 (10.66,52.4)	1 (0,2)	37.85 (16.48,81.62)	1.406(1.345,1.467)	<0.001
Costa Rica	116 (51,251)	25.35 (11.18,54.77)	191 (84,411)	35.03 (15.34,75.12)	1.063(1.004,1.121)	<0.001

Coted'Ivoire	98 (40,215)	5.12 (2.11,11.22)	361 (158,764)	8.67 (3.79,18.35)	1.736(1.628,1.844)	<0.001
Croatia	9 (3,20)	1.83 (0.68,3.95)	8 (3,18)	2.57 (1.01,5.53)	1.121(1.039,1.203)	<0.001
Cuba	272 (116,571)	17.92 (7.64,37.57)	213 (92,439)	22.58 (9.78,46.51)	0.75(0.64,0.86)	<0.001
Cyprus	26 (11,53)	28.07 (12.41,57.7)	43 (19,91)	41.51 (17.95,87.39)	1.237(1.131,1.342)	<0.001
Czechia	21 (8,46)	1.83 (0.7,3.98)	18 (7,37)	2.33 (0.93,4.82)	0.817(0.628,1.007)	<0.001
Democratic People's Republic of Korea	319 (140,685)	10.74 (4.7,23.08)	320 (136,678)	11.98 (5.11,25.4)	0.347(0.265,0.429)	<0.001
Democratic Republic of the Congo	273 (114,594)	4.57 (1.91,9.95)	1020 (442,2142)	6.95 (3.01,14.6)	1.373(1.21,1.537)	<0.001
Denmark	167 (73,348)	31.31 (13.77,65.31)	207 (91,425)	40.37 (17.86,82.97)	0.819(0.768,0.87)	<0.001
Djibouti	4 (2,8)	5.67 (2.4,11.9)	16 (7,34)	10.2 (4.36,21.29)	1.882(1.727,2.038)	<0.001
Dominica	2 (1,3)	14.63 (6.27,30.24)	2 (1,3)	20.58 (9.19,43.57)	1.101(1.029,1.173)	<0.001
Dominican Republic	155 (67,321)	12.62 (5.48,26.16)	295 (127,619)	20.83 (8.96,43.72)	1.632(1.571,1.694)	<0.001
Ecuador	547 (241,1163)	33.3 (14.66,70.72)	1086 (469,2262)	45.27 (19.53,94.31)	1.015(0.951,1.08)	<0.001
Egypt	1888 (820,3887)	22.77 (9.89,46.86)	4087 (1775,8313)	28.52 (12.39,58.01)	0.733(0.671,0.795)	<0.001

El Salvador	179 (78,386)	19.85 (8.64,42.69)	261 (113,561)	29.96 (12.96,64.38)	1.364(1.271,1.458)	<0.001
Equatorial Guinea	3 (1,7)	4.96 (2.05,10.55)	29 (13,61)	12.74 (5.54,26.64)	3.097(2.858,3.337)	<0.001
Eritrea	23 (9,47)	4.13 (1.66,8.61)	66 (27,139)	6.54 (2.71,13.83)	1.567(1.39,1.744)	<0.001
Estonia	4 (2,8)	2.46 (0.95,5.25)	3 (1,7)	3.44 (1.37,7.56)	1.087(0.99,1.184)	<0.001
Eswatini	15 (6,30)	10.21 (4.27,20.6)	24 (10,50)	13.43 (5.61,27.71)	0.908(0.805,1.012)	<0.001
Ethiopia	306 (128,636)	3.81 (1.6,7.94)	1196 (510,2528)	6.44 (2.75,13.62)	1.718(1.638,1.799)	<0.001
Fiji	22 (10,46)	18.96 (8.54,39.75)	34 (15,71)	29.2 (13.17,60.87)	1.4(1.325,1.475)	<0.001
Finland	146 (66,299)	30.73 (13.81,62.73)	180 (79,366)	40.2 (17.69,81.81)	0.867(0.805,0.929)	<0.001
France	2018 (880,4150)	32.54 (14.2,66.93)	2371 (1046,4907)	39.67 (17.5,82.08)	0.632(0.578,0.686)	<0.001
Gabon	11 (5,24)	7.13 (2.96,15.49)	34 (14,72)	11.34 (4.76,24.12)	1.529(1.385,1.673)	<0.001
Gambia	8 (3,18)	5.08 (2.11,11)	33 (14,66)	8.01 (3.35,15.95)	1.495(1.328,1.662)	<0.001
Georgia	31 (13,68)	4.94 (2.01,10.68)	25 (11,54)	8.66 (3.71,18.36)	1.851(1.729,1.973)	<0.001
Germany	2379 (1083,4969)	32.81 (14.93,68.53)	2276 (1001,4507)	38.09 (16.74,75.41)	0.473(0.406,0.541)	<0.001

Ghana	124 (50,268)	5.27 (2.14,11.35)	440 (184,904)	8.38 (3.51,17.21)	1.516(1.299,1.733)	<0.001
Greece	411 (182,844)	35.77 (15.84,73.54)	333 (145,682)	43.93 (19.17,89.97)	0.637(0.564,0.71)	<0.001
Greenland	1 (0,2)	16.04 (7.18,32.84)	1 (0,2)	19.22 (8.45,39.55)	0.577(0.486,0.667)	<0.001
Grenada	2 (1,3)	12.08 (4.97,25.08)	2 (1,5)	19.21 (8.55,41.36)	1.527(1.43,1.624)	<0.001
Guam	5 (2,10)	27.37 (11.89,55.67)	7 (3,14)	37.7 (16.67,78.17)	1.027(0.956,1.098)	<0.001
Guatemala	214 (91,455)	15.6 (6.63,33.16)	634 (273,1336)	25.44 (10.97,53.63)	1.586(1.455,1.717)	<0.001
Guinea	38 (15,82)	4.45 (1.8,9.52)	157 (66,331)	7.07 (2.97,14.91)	1.527(1.413,1.64)	<0.001
Guinea-Bissau	7 (3,16)	4.45 (1.87,9.73)	24 (10,51)	7.13 (2.96,15.07)	1.526(1.388,1.664)	<0.001
Guyana	17 (7,36)	13 (5.45,27.43)	20 (9,43)	19.79 (8.38,41.52)	1.401(1.323,1.479)	<0.001
Haiti	92 (39,201)	9.2 (3.88,20.04)	215 (94,464)	11.49 (5.05,24.81)	0.756(0.648,0.864)	<0.001
Honduras	121 (52,269)	15.61 (6.78,34.81)	409 (179,878)	25.64 (11.22,55)	1.624(1.567,1.681)	<0.001
Hungary	22 (9,53)	1.98 (0.8,4.72)	18 (8,40)	2.58 (1.08,5.69)	0.862(0.749,0.975)	<0.001
Iceland	11 (5,24)	36.85 (16.75,75.92)	15 (7,31)	45.21 (20.19,92.71)	0.645(0.532,0.758)	<0.001

India	9530 (4263,19844)	7.7 (3.45,16.04)	27787 (12105,58016)	14.55 (6.34,30.38)	2.095(2.02,2.171)	<0.001
Indonesia	5236 (2288,10785)	17.54 (7.66,36.13)	11872 (5365,24583)	35.39 (16,73.29)	2.297(2.253,2.341)	<0.001
Iran (Islamic Republic of)	1800 (790,3749)	19.85 (8.72,41.35)	2379 (1044,5000)	27.52 (12.08,57.84)	1.058(0.752,1.364)	<0.001
Iraq	616 (269,1271)	20.91 (9.13,43.15)	1499 (650,3117)	24.76 (10.73,51.47)	0.611(0.463,0.759)	<0.001
Ireland	155 (67,308)	32.53 (14.12,64.81)	196 (85,395)	40.58 (17.7,81.81)	0.721(0.644,0.798)	<0.001
Israel	187 (82,387)	28.06 (12.34,58.16)	404 (178,845)	36.7 (16.2,76.8)	0.866(0.824,0.907)	<0.001
Italy	6669 (2930,14187)	108.71 (47.75,231.26)	4228 (1870,8963)	100.84 (44.61,213.76)	-0.245(-0.326,-0.165)	<0.001
Jamaica	55 (24,115)	14.29 (6.24,29.87)	71 (30,150)	20.43 (8.68,43.12)	1.179(1.082,1.275)	<0.001
Japan	9067 (3961,18313)	66.09 (28.87,133.47)	6088 (2699,12273)	71.14 (31.55,143.42)	0.223(0.124,0.322)	<0.001
Jordan	129 (57,268)	20.22 (8.87,41.98)	467 (203,969)	25.95 (11.26,53.88)	0.822(0.699,0.944)	<0.001
Kazakhstan	91 (38,196)	4.26 (1.76,9.14)	119 (49,256)	5.92 (2.42,12.7)	1.087(0.98,1.193)	<0.001
Kenya	249 (107,530)	6.18 (2.66,13.14)	699 (303,1487)	8.1 (3.51,17.23)	0.927(0.716,1.138)	<0.001
Kiribati	2 (1,4)	16.4 (7.33,34.63)	4 (2,9)	23.41 (10.25,48.19)	1.146(1.071,1.222)	<0.001

Kuwait	64 (28,133)	28.29 (12.28,58.81)	140 (61,299)	34.55 (15.04,73.69)	0.656(0.593,0.718)	<0.001
Kyrgyzstan	24 (10,54)	3.81 (1.55,8.45)	40 (17,86)	4.6 (1.89,9.87)	0.616(0.511,0.722)	<0.001
Lao People's Democratic Republic	95 (42,197)	14.34 (6.28,29.78)	302 (135,607)	29.21 (13.11,58.72)	2.333(2.25,2.415)	<0.001
Latvia	7 (3,15)	2.48 (0.99,5.52)	4 (2,9)	3.11 (1.24,6.74)	0.699(0.565,0.833)	<0.001
Lebanon	92 (40,188)	21.62 (9.45,44.26)	158 (68,328)	28.34 (12.24,58.76)	0.895(0.768,1.021)	<0.001
Lesotho	17 (7,37)	6.76 (2.95,14.54)	34 (15,77)	11.27 (4.86,25.18)	1.714(1.634,1.794)	<0.001
Liberia	20 (8,42)	5.35 (2.28,11.39)	70 (30,150)	7.85 (3.33,16.76)	1.268(1.093,1.443)	<0.001
Libya	171 (74,358)	23.5 (10.2,49.14)	244 (106,514)	28.55 (12.38,60.12)	0.65(0.569,0.73)	<0.001
Lithuania	9 (3,20)	2.24 (0.86,5.11)	6 (2,13)	3.17 (1.2,6.64)	1.101(0.948,1.255)	<0.001
Luxembourg	13 (6,28)	37.3 (16.74,77.57)	23 (10,49)	45.46 (20.31,93.9)	0.65(0.564,0.736)	<0.001
Madagascar	100 (42,216)	5.16 (2.18,11.19)	305 (128,638)	6.26 (2.63,13.07)	0.642(0.549,0.736)	<0.001
Malawi	117 (49,251)	7.26 (3.03,15.59)	287 (127,566)	7.92 (3.51,15.58)	0.285(0.154,0.416)	<0.001
Malaysia	717 (319,1474)	26.9 (11.98,55.32)	1959 (858,3975)	49.88 (21.83,101.19)	2.011(1.87,2.152)	<0.001

Maldives	6 (3,12)	16.25 (7,33.77)	17 (8,38)	38.91 (16.9,84.39)	2.866(2.657,3.075)	<0.001
Mali	50 (20,108)	3.85 (1.57,8.3)	255 (108,522)	6.37 (2.69,13.03)	1.649(1.511,1.786)	<0.001
Malta	12 (5,25)	31.29 (13.79,63.24)	14 (6,28)	45.05 (19.57,92.14)	1.195(1.137,1.253)	<0.001
Marshall Islands	1 (0,2)	12.39 (5.45,27.11)	2 (1,4)	23.16 (10.25,47.92)	2.032(1.97,2.093)	<0.001
Mauritania	22 (9,46)	6.71 (2.88,14.42)	71 (30,145)	9.64 (4.14,19.79)	1.188(1.068,1.308)	<0.001
Mauritius	43 (19,90)	26.99 (11.87,56.6)	61 (27,127)	47.35 (21.02,99.17)	1.84(1.721,1.96)	<0.001
Mexico	6284 (2765,13268)	42.68 (18.78,90.1)	7398 (3261,15338)	44.83 (19.76,92.95)	0.134(-0.027,0.296)	0.103
Micronesia (Federated States of)	3 (1,6)	16.41 (7.2,33.81)	4 (2,8)	25.86 (11.47,52.61)	1.469(1.434,1.504)	<0.001
Monaco	1 (0,2)	42.19 (18.5,87.16)	1 (1,2)	45.14 (19.81,94.56)	0.21(0.16,0.26)	<0.001
Mongolia	13 (5,28)	3.62 (1.47,7.99)	19 (8,44)	5.22 (2.16,11.66)	1.179(1.041,1.318)	<0.001
Montenegro	1 (1,3)	1.96 (0.75,4.25)	1 (1,3)	2.61 (1.01,5.82)	0.941(0.877,1.006)	<0.001
Morocco	761 (330,1581)	18.73 (8.12,38.91)	1178 (514,2431)	25.69 (11.2,53.02)	1.075(0.938,1.212)	<0.001
Mozambique	107 (44,223)	4.9 (2,10.24)	432 (182,938)	7.84 (3.31,17.04)	1.571(1.407,1.734)	<0.001

Myanmar	915 (391,1857)	13.85 (5.92,28.11)	2285 (1023,4713)	29.95 (13.4,61.78)	2.553(2.468,2.638)	<0.001
Namibia	17 (7,38)	7.22 (3.07,15.63)	39 (17,80)	10.26 (4.61,21.22)	1.166(1.021,1.312)	<0.001
Nauru	0 (0,1)	20.03 (8.78,41.2)	0 (0,1)	29.72 (13.23,63.25)	1.255(1.156,1.353)	<0.001
Nepal	123 (50,254)	4.09 (1.67,8.44)	410 (173,862)	8.53 (3.6,17.94)	2.397(2.236,2.558)	<0.001
Netherlands	529 (238,1080)	33.01 (14.83,67.36)	582 (256,1191)	39.56 (17.37,80.94)	0.559(0.508,0.61)	<0.001
New Zealand	282 (124,577)	67.92 (29.98,139.31)	330 (147,686)	67.57 (30.14,140.46)	-0.023(-0.083,0.036)	0.442
Nicaragua	121 (51,258)	18.05 (7.6,38.53)	252 (109,553)	27.41 (11.88,60.06)	1.355(1.29,1.421)	<0.001
Niger	52 (22,108)	4.05 (1.7,8.49)	235 (94,507)	5.5 (2.21,11.86)	1.009(0.925,1.092)	<0.001
Nigeria	843 (362,1785)	5.62 (2.41,11.91)	3201 (1373,6734)	7.84 (3.36,16.48)	1.102(0.974,1.231)	<0.001
Niue	0 (0,0)	21.34 (9.31,44.21)	0 (0,0)	34.65 (15.12,69.86)	1.607(1.372,1.842)	<0.001
North Macedonia	4 (1,9)	1.64 (0.57,3.57)	4 (2,9)	2.41 (0.99,5.16)	1.282(1.162,1.401)	<0.001
Northern Mariana Islands	2 (1,4)	27.62 (12.27,58.45)	2 (1,4)	31.94 (14.03,67.99)	0.405(-0.179,0.993)	0.175
Norway	164 (73,338)	36.73 (16.42,75.44)	191 (84,391)	39.96 (17.52,81.62)	0.268(0.188,0.347)	<0.001

Oman	38 (16,81)	15.64 (6.62,33.34)	123 (54,257)	28.39 (12.41,59.22)	1.961(1.893,2.028)	<0.001
Pakistan	1209 (508,2656)	7.14 (3,15.69)	3059 (1305,6511)	8.75 (3.73,18.62)	0.682(0.551,0.813)	<0.001
Palau	1 (0,1)	24.01 (10.3,50.7)	1 (0,1)	33.56 (14.63,68.68)	1.052(0.969,1.135)	<0.001
Palestine	60 (25,127)	18.08 (7.66,38.24)	189 (83,398)	23.47 (10.34,49.38)	0.857(0.752,0.962)	<0.001
Panama	66 (28,143)	17.65 (7.55,38.27)	154 (67,334)	28.63 (12.45,62.19)	1.57(1.483,1.658)	<0.001
Papua New Guinea	78 (34,165)	12.48 (5.39,26.17)	270 (120,573)	18.63 (8.27,39.56)	1.293(1.187,1.399)	<0.001
Paraguay	26 (11,54)	4.22 (1.72,8.73)	70 (30,145)	7.17 (3.07,14.9)	1.746(1.658,1.835)	<0.001
Peru	945 (403,2119)	26.72 (11.39,59.9)	1732 (756,3717)	39.72 (17.34,85.23)	1.305(1.127,1.484)	<0.001
Philippines	1833 (773,3683)	18 (7.59,36.16)	5184 (2207,10416)	32.73 (13.93,65.75)	1.976(1.889,2.062)	<0.001
Poland	120 (50,248)	2.86 (1.19,5.91)	93 (42,194)	3.29 (1.47,6.82)	0.453(0.4,0.506)	<0.001
Portugal	358 (160,725)	29.09 (13.02,58.97)	329 (149,666)	41 (18.54,82.85)	1.09(1.012,1.168)	<0.001
Puerto Rico	110 (46,242)	22.47 (9.46,49.59)	91 (41,193)	30.1 (13.46,63.9)	0.962(0.908,1.016)	<0.001
Qatar	10 (4,21)	26.15 (11.11,52.7)	53 (23,110)	31.1 (13.77,64.77)	0.58(0.461,0.7)	<0.001

Republic of Korea	1440 (640,2936)	22.62 (10.05,46.12)	1365 (595,2892)	36.63 (15.96,77.63)	1.645(1.462,1.827)	<0.001
Republic of Moldova	9 (4,19)	1.82 (0.72,3.79)	8 (3,17)	2.9 (1.12,6.27)	1.542(1.401,1.683)	<0.001
Romania	49 (19,106)	1.69 (0.65,3.64)	36 (15,76)	2.4 (0.99,5.04)	1.164(0.988,1.34)	<0.001
Russian Federation	339 (134,760)	2.2 (0.87,4.92)	326 (132,719)	2.84 (1.14,6.26)	0.866(0.732,1.001)	<0.001
Rwanda	71 (30,151)	6.14 (2.61,13.08)	178 (75,384)	8.19 (3.46,17.62)	0.908(0.829,0.987)	<0.001
Saint Kitts and Nevis	1 (0,2)	16.73 (7.21,35.35)	2 (1,3)	24.8 (10.8,51.14)	1.269(1.21,1.328)	<0.001
Saint Lucia	3 (1,6)	13.75 (5.9,28.25)	4 (2,8)	20.03 (8.86,42.46)	1.213(1.155,1.271)	<0.001
Saint Vincent and the Grenadines	2 (1,5)	12.36 (5.17,26.97)	3 (1,5)	19.66 (8.79,41.22)	1.542(1.503,1.581)	<0.001
Samoa	5 (2,11)	20.23 (8.83,41.71)	8 (4,18)	27.75 (12.19,58.89)	1.02(0.943,1.098)	<0.001
San Marino	1 (0,2)	38.56 (17.18,80.26)	1 (1,2)	43.8 (19.08,90.94)	0.396(0.356,0.436)	<0.001
Sao Tome and Principe	1 (0,2)	5.25 (2.2,11.61)	3 (1,6)	8.54 (3.64,17.58)	1.578(1.461,1.694)	<0.001
Saudi Arabia	504 (218,1026)	21.66 (9.38,44.16)	1241 (554,2649)	33.84 (15.11,72.26)	1.467(1.409,1.525)	<0.001
Senegal	76 (32,167)	6.01 (2.56,13.31)	200 (89,402)	7.91 (3.52,15.95)	0.895(0.768,1.022)	<0.001

Serbia	18 (7,39)	1.67 (0.66,3.64)	18 (7,39)	2.37 (0.96,5.07)	1.155(1.067,1.243)	<0.001
Seychelles	3 (1,7)	29.4 (12.97,61.89)	4 (2,9)	41.25 (18.03,85.34)	1.1(1.025,1.176)	<0.001
Sierra Leone	29 (12,60)	4.58 (1.91,9.5)	121 (51,260)	8.15 (3.46,17.49)	1.857(1.668,2.047)	<0.001
Singapore	112 (50,234)	28.39 (12.75,59.26)	146 (66,314)	41.08 (18.64,88.14)	1.23(1.125,1.335)	<0.001
Slovakia	11 (4,25)	1.68 (0.64,3.97)	10 (4,22)	2.47 (0.94,5.41)	1.254(1.111,1.397)	<0.001
Slovenia	4 (1,8)	1.8 (0.68,3.88)	4 (1,7)	2.52 (1.03,5.21)	1.084(1.011,1.156)	<0.001
Solomon Islands	7 (3,15)	12.95 (5.75,27.23)	22 (10,46)	21.14 (9.4,45.11)	1.563(1.475,1.652)	<0.001
Somalia	56 (23,122)	4.8 (1.99,10.39)	211 (87,455)	6.07 (2.49,13.09)	0.769(0.664,0.874)	<0.001
South Africa	644 (276,1386)	10.66 (4.56,22.94)	961 (420,1985)	13.61 (5.95,28.11)	0.83(0.766,0.893)	<0.001
South Sudan	55 (23,121)	5.81 (2.41,12.83)	106 (46,235)	6.39 (2.76,14.15)	0.333(0.161,0.505)	<0.001
Spain	1479 (663,3072)	31.09 (13.93,64.59)	1321 (571,2773)	38.74 (16.74,81.35)	0.715(0.656,0.773)	<0.001
Sri Lanka	578 (260,1181)	22.48 (10.09,45.89)	975 (436,1968)	37.37 (16.71,75.42)	1.66(1.541,1.779)	<0.001
Sudan	383 (167,838)	11.89 (5.2,26.03)	1477 (656,3087)	21.25 (9.43,44.42)	1.899(1.83,1.969)	<0.001

Suriname	9 (4,18)	14.69 (6.08,30.59)	14 (6,31)	20.12 (9,44.51)	1.038(0.962,1.114)	<0.001
Sweden	224 (98,464)	27.71 (12.13,57.42)	265 (117,547)	31 (13.75,64.06)	0.378(0.276,0.48)	<0.001
Switzerland	236 (105,491)	36.61 (16.32,76.12)	255 (112,514)	38.92 (17.03,78.32)	0.186(0.135,0.238)	<0.001
Syrian Arab Republic	374 (166,797)	17.62 (7.8,37.53)	615 (270,1296)	25.53 (11.21,53.82)	1.236(1.089,1.383)	<0.001
Taiwan (Province of China)	505 (222,1051)	18.15 (7.99,37.73)	543 (237,1095)	32.25 (14.04,64.99)	1.885(1.735,2.035)	<0.001
Tajikistan	25 (10,55)	3.02 (1.24,6.61)	55 (22,121)	4.11 (1.62,8.93)	0.979(0.845,1.113)	<0.001
Thailand	1929 (857,3911)	21.86 (9.71,44.32)	2580 (1169,5419)	44.33 (20.08,93.09)	2.344(2.246,2.441)	<0.001
Timor-Leste	16 (7,31)	14.55 (6.38,28.87)	56 (25,117)	24.18 (10.86,50.58)	1.678(1.561,1.794)	<0.001
Togo	28 (12,59)	4.69 (1.93,9.89)	96 (41,206)	7.55 (3.22,16.13)	1.559(1.413,1.705)	<0.001
Tokelau	0 (0,0)	18.37 (8.12,38.88)	0 (0,0)	29.73 (13.33,61.92)	1.573(1.465,1.68)	<0.001
Tonga	4 (2,8)	23.08 (9.95,47.68)	5 (2,10)	32.43 (14.2,66.81)	1.081(1.014,1.149)	<0.001
Trinidad and Tobago	24 (11,52)	14.31 (6.28,30.85)	29 (13,60)	21.58 (9.37,44.86)	1.355(1.239,1.471)	<0.001
Tunisia	241 (104,503)	18.35 (7.9,38.23)	317 (140,668)	25.95 (11.42,54.65)	1.189(1.092,1.286)	<0.001

Turkey	1568 (683,3256)	17.44 (7.6,36.23)	2337 (1034,5019)	24.91 (11.02,53.5)	1.207(1.099,1.315)	<0.001
Turkmenistan	22 (9,48)	3.93 (1.61,8.39)	38 (15,81)	5.92 (2.42,12.65)	1.361(1.277,1.446)	<0.001
Tuvalu	0 (0,0)	17.81 (8.08,36.39)	0 (0,1)	28.17 (12.59,59.9)	1.465(1.4,1.53)	<0.001
Uganda	172 (71,371)	5.94 (2.46,12.77)	564 (240,1152)	7.46 (3.17,15.25)	0.752(0.676,0.828)	<0.001
Ukraine	113 (42,242)	2.1 (0.79,4.51)	79 (31,178)	2.47 (0.98,5.58)	0.563(0.389,0.736)	<0.001
United Arab Emirates	40 (18,83)	21.92 (9.58,45.18)	158 (69,332)	29.45 (12.8,61.73)	1.001(0.851,1.151)	<0.001
United Kingdom	2354 (1043,4818)	40.43 (17.92,82.76)	2822 (1244,5842)	47.42 (20.91,98.18)	0.515(0.484,0.545)	<0.001
United Republic of Tanzania	305 (128,628)	6.91 (2.89,14.2)	839 (371,1791)	8.47 (3.74,18.08)	0.669(0.595,0.743)	<0.001
United States of America	10264 (4578,20587)	37.98 (16.94,76.17)	14446 (6731,28930)	45.54 (21.22,91.21)	0.531(0.294,0.768)	<0.001
United States Virgin Islands	3 (1,6)	21.1 (9.18,45.36)	2 (1,4)	27.21 (12.02,59.4)	0.825(0.759,0.891)	<0.001
Uruguay	51 (22,104)	13.48 (5.88,27.49)	84 (38,174)	23.83 (10.79,49.01)	1.887(1.838,1.936)	<0.001
Uzbekistan	127 (52,281)	3.98 (1.63,8.81)	227 (98,491)	5.82 (2.5,12.56)	1.263(1.11,1.416)	<0.001
Vanuatu	4 (2,7)	15.21 (6.8,31.7)	11 (5,23)	23.59 (10.27,49.04)	1.415(1.37,1.46)	<0.001

Venezuela (Bolivarian Republic of)	722 (309,1504)	24.19 (10.37,50.39)	909 (400,1898)	30.26 (13.33,63.23)	0.706(0.604,0.809)	<0.001
Viet Nam	1369 (596,2842)	12.64 (5.51,26.25)	2644 (1144,5438)	25.75 (11.14,52.96)	2.338(2.214,2.462)	<0.001
Yemen	228 (99,478)	11.23 (4.88,23.56)	805 (349,1721)	14.9 (6.45,31.84)	0.939(0.828,1.049)	<0.001
Zambia	115 (48,253)	8.02 (3.33,17.66)	337 (143,713)	9.99 (4.25,21.16)	0.73(0.621,0.839)	<0.001
Zimbabwe	152 (66,325)	8.31 (3.59,17.75)	240 (103,516)	9.26 (3.98,19.86)	0.364(0.266,0.463)	<0.001

Table S8. Age-standardized rate of PCOS in adolescents and young adults aged 10-24 years in 2021 and their AAPCs between 1990-2021 in 204 countries and territories

	Incidence				Prevalence				DALYs			
	Age-standardized rate (per 100 000 population), 1990	Age-standardized rate (per 100 000 population), 2021	AAPC	*P* value	Age-standardized rate (per 100 000 population), 1990	Age-standardized rate (per 100 000 population), 2021	AAPC	*P* value	Age-standardized rate (per 100 000 population), 1990	Age-standardized rate (per 100 000 population), 2021	AAPC	*P* value
Afghanistan	34.78 (24.32,48.87)	50.11 (34.4,71.4)	1.231(1.085,1.377)	0	899.66 (622.33,1286.08)	1295.31 (889.64,1847.76)	1.232(1.08,1.384)	<0.001	7.67 (3.36,16.31)	11.11 (4.78,23.89)	1.256(1.09,1.422)	<0.001
Albania	5.8 (3.89,8.74)	7.7 (5.23,11.2	0.936(0.845,1.027)	0	145.74 (96.31,222.5	191.44 (126.23,291.	0.912(0.787,1.037)	<0.001	1.28 (0.52,2.76	1.69 (0.72,3.69	0.922(0.79,1.055)	<0.001

		9)			2)	31)			)	)		
Algeria	57.74 (40.27,81.59)	84.01 (56.67,120.59)	1.249(1.163,1.334)	0	1505.48 (1043.68,2150.72)	2205.99 (1497.75,3172.01)	1.259(1.213,1.306)	<0.001	13.39 (5.72,26.79)	19.42 (8.47,40.73)	1.24(1.202,1.277)	<0.001
American Samoa	78.14 (54.84,109.65)	108.31 (75.38,157.79)	1.049(1.025,1.073)	0	2015.89 (1400,2848.69)	2789.56 (1942.9,4075)	1.044(1.02,1.067)	<0.001	17.71 (7.67,37.05)	24.41 (10.56,51.54)	1.033(1.011,1.056)	<0.001
Andorra	113.41 (77.62,162.49)	140.95 (97.02,199.68)	0.707(0.689,0.724)	0	2892.36 (1997.17,4119.21)	3580.57 (2469.01,5055.23)	0.694(0.672,0.715)	<0.001	25.98 (11.68,53.94)	32.02 (14.34,67.46)	0.675(0.642,0.707)	<0.001
Angola	16.18 (11.29,22.74)	28.41 (19.49,40.98)	1.859(1.764,1.954)	0	407.71 (281.65,588.77)	734.31 (499.67,1062.3)	1.943(1.84,2.047)	<0.001	3.51 (1.49,7.15)	6.34 (2.72,12.95)	1.957(1.85,2.064)	<0.001
Antigua and Barbuda	45.57 (30.83,63.86)	60.64 (41.8,86.38)	0.921(0.853,0.99)	0	1184.04 (795.74,1697.4)	1577.69 (1081.35,2242.53)	0.922(0.861,0.984)	<0.001	10.48 (4.48,21.66)	13.92 (6.13,28.54)	0.915(0.858,0.972)	<0.001
Argentina	45.96 (31.87,65.7)	70.92 (50.34,101.64)	1.445(1.402,1.488)	0	1164.03 (804.69,1679.22)	1794.47 (1259.48,2552.32)	1.424(1.354,1.494)	<0.001	10.31 (4.61,21.22)	15.89 (7.02,32.73)	1.422(1.377,1.466)	<0.001
Armenia	12.77 (8.85,18.3)	19.23 (13.21,27.95)	1.344(1.285,1.403)	0	321.28 (217.72,473.58)	478.73 (323.9,700.39)	1.311(1.268,1.354)	<0.001	2.79 (1.15,6.05)	4.15 (1.79,8.78)	1.302(1.251,1.353)	<0.001
Australia	160.56 (117.92,208.47)	195.1 (138.82,272.46)	0.615(0.56,0.669)	0	3818.98 (2800.78,4961.92)	4606.14 (3265.61,6417.26)	0.594(0.54,0.649)	<0.001	33.35 (15.01,68.31)	40.2 (18.03,83.48)	0.602(0.548,0.657)	<0.001
Austria	144.37	152.33	0.161(0.116,	0	3733.72	3942.67	0.173(0.096,	<0.0	33.14	34.94	0.154(0.077,	<0.0

	(103.01,198.9)	(105.34,213.46)	0.205)		(2614.91,5155.02)	(2723.35,5569.98)	0.251)	01	(14.93,67.79)	(15.26,71.48)	0.23)	01
Azerbaijan	12.95 (9.01,18.48)	19.73 (13.21,27.45)	1.375(1.328,1.421)	0	327.36 (221.69,486.18)	505.58 (337.65,727.07)	1.419(1.377,1.461)	<0.001	2.9 (1.19,6.23)	4.46 (1.81,9.72)	1.404(1.327,1.481)	<0.001
Bahamas	57.85 (38.8,83.78)	69.02 (46.9,100.8)	0.582(0.558,0.607)	0	1506.12 (1025.77,2176.77)	1796.01 (1213.4,2608.97)	0.573(0.559,0.588)	<0.001	13.32 (5.68,28.05)	15.86 (7.03,33.97)	0.566(0.526,0.607)	<0.001
Bahrain	80.02 (54.78,114.15)	91.28 (63.71,129.23)	0.463(0.416,0.51)	0	2104.49 (1428.61,2989.85)	2399.15 (1652.22,3399.21)	0.463(0.416,0.51)	<0.001	18.57 (7.92,38.78)	21.12 (9.3,44.61)	0.463(0.415,0.511)	<0.001
Bangladesh	14.95 (10.29,21.01)	24.43 (17.17,33.89)	1.608(1.477,1.74)	0	371.45 (253.24,531.81)	614.52 (422.62,875.78)	1.655(1.561,1.749)	<0.001	3.3 (1.41,6.91)	5.43 (2.32,11.5)	1.624(1.487,1.762)	<0.001
Barbados	57.13 (38.35,81.8)	67.19 (45.35,94.29)	0.536(0.473,0.599)	0	1485.65 (1014.05,2116.97)	1747.68 (1187.58,2498.66)	0.537(0.473,0.601)	<0.001	13.14 (5.79,27.84)	15.41 (6.75,33)	0.523(0.451,0.596)	<0.001
Belarus	8.06 (5.53,11.58)	10.64 (7.38,15.35)	0.925(0.87,0.979)	0	199.64 (134.86,295.4)	265.96 (180.29,396.6)	0.956(0.926,0.987)	<0.001	1.77 (0.73,3.79)	2.35 (0.98,5.15)	0.94(0.872,1.008)	<0.001
Belgium	116.37 (79.66,167.29)	137.03 (96.6,192.74)	0.54(0.5,0.58)	0	2993.3 (2057.38,4280.33)	3545.39 (2465.51,4947.93)	0.561(0.52,0.603)	<0.001	26.96 (11.92,55.81)	31.59 (14.47,66.14)	0.529(0.484,0.575)	<0.001
Belize	45.72 (31.04,64.98)	65.92 (44.95,94.84)	1.202(1.13,1.274)	0	1175.11 (799.55,1652.17)	1698.95 (1166.35,2399.27)	1.206(1.135,1.278)	<0.001	10.55 (4.66,22.06)	15.12 (6.67,32.25)	1.177(1.095,1.259)	<0.001

Benin	18.29 (12.86,25.72)	32.87 (22.4,46.94)	1.911(1.807,2.016)	0	458.38 (316.55,656.35)	841.31 (570.3,1209.54)	1.981(1.878,2.085)	<0.001	3.91 (1.7,8.3)	7.2 (3.11,15.23)	1.999(1.847,2.151)	<0.001
Bermuda	69.24 (45.53,99.42)	77.01 (52.53,113.93)	0.355(0.293,0.417)	0	1802.08 (1183.92,2586.26)	2003.91 (1376.5,2920.75)	0.35(0.298,0.402)	<0.001	15.96 (6.92,33.23)	17.71 (7.61,36.75)	0.346(0.287,0.405)	<0.001
Bhutan	21.13 (14.8,29.45)	36.96 (25.68,52.75)	1.835(1.791,1.878)	0	535.23 (369.69,764.72)	951.35 (651.15,1365.52)	1.887(1.841,1.934)	<0.001	4.73 (2.02,9.85)	8.41 (3.68,17.67)	1.897(1.835,1.959)	<0.001
Bolivia (Plurinational State of)	91.21 (61.22,132.39)	120.85 (81.51,169.9)	0.923(0.899,0.947)	0	2240.54 (1511.12,3238.64)	3013.26 (2024.42,4266.89)	0.986(0.925,1.048)	<0.001	19.44 (8.33,41.19)	26.01 (11.34,55.81)	0.968(0.896,1.04)	<0.001
Bosnia and Herzegovina	4.93 (3.25,7.58)	7.59 (5.07,11.17)	1.429(1.337,1.521)	0	123.47 (79.04,194.68)	191.47 (125.22,293.78)	1.449(1.257,1.641)	<0.001	1.08 (0.45,2.28)	1.67 (0.7,3.64)	1.433(1.365,1.502)	<0.001
Botswana	27.37 (19.32,39.21)	42.27 (28.68,59.53)	1.423(1.255,1.591)	0	688.23 (471.44,985.2)	1081.33 (733.16,1530.54)	1.476(1.315,1.637)	<0.001	5.88 (2.51,12.62)	9.23 (4.02,19.29)	1.473(1.305,1.642)	<0.001
Brazil	23.54 (16.12,33.61)	24.97 (17.19,35.09)	0.181(0.086,0.276)	0	562.85 (382.22,807.58)	610.2 (419.76,868.28)	0.245(0.081,0.409)	0.003	5.01 (2.13,10.55)	5.38 (2.33,11.29)	0.218(0.127,0.309)	<0.001
Brunei Darussalam	101.06 (70.16,145.53)	160.01 (111.25,224.46)	1.498(1.444,1.551)	0	2426.72 (1692.08,3533.53)	3797.3 (2681.33,5308.98)	1.463(1.424,1.501)	<0.001	21.24 (9.32,43.91)	33.12 (15.02,68)	1.45(1.412,1.487)	<0.001

Bulgaria	6.76 (4.49,10.01)	8.65 (5.87,12.8)	0.811(0.776,0.847)	0	171.08 (108.99,259.83)	218.84 (144.56,326.54)	0.81(0.77,0.851)	<0.001	1.49 (0.61,3.22)	1.9 (0.77,3.97)	0.811(0.75,0.872)	<0.001
Burkina Faso	17.36 (12.19,24.59)	27.1 (18.9,37.99)	1.456(1.382,1.53)	0	432.11 (296.79,624.25)	691.63 (474.65,990.3)	1.548(1.471,1.626)	<0.001	3.8 (1.59,8.15)	5.98 (2.53,12.65)	1.469(1.384,1.555)	<0.001
Burundi	17.44 (12.3,24.45)	18.62 (13,25.75)	0.208(0.164,0.252)	0	435.65 (302.05,617.45)	460.99 (314.65,664.66)	0.185(0.138,0.233)	<0.001	3.78 (1.61,8.15)	3.98 (1.68,8.3)	0.16(0.123,0.196)	<0.001
Cabo Verde	19.72 (13.89,27.83)	32.43 (22.54,45.9)	1.625(1.514,1.735)	0	498.89 (347.78,717.89)	837.21 (575.08,1210.69)	1.686(1.611,1.761)	<0.001	4.32 (1.86,9.06)	7.22 (3.12,15.1)	1.679(1.597,1.761)	<0.001
Cambodia	45.59 (31.31,64.68)	78.36 (54.92,111.79)	1.797(1.752,1.842)	0	1159.79 (803.42,1661.48)	2006.79 (1398.68,2881.22)	1.807(1.746,1.868)	<0.001	10.04 (4.38,19.89)	17.44 (7.75,35.71)	1.827(1.768,1.886)	<0.001
Cameroon	26.78 (18.38,38.15)	35.51 (23.66,50.89)	0.916(0.857,0.976)	0	687.74 (465.13,991.43)	922.85 (613.78,1361.89)	0.96(0.896,1.023)	<0.001	5.98 (2.53,12.73)	8.08 (3.48,17.05)	0.992(0.895,1.089)	<0.001
Canada	57.32 (39.7,82.42)	73.82 (51.22,106.18)	0.815(0.779,0.851)	0	1436.89 (997.82,2061.2)	1841.49 (1289.91,2661.42)	0.784(0.729,0.838)	<0.001	12.89 (5.68,26.49)	16.48 (7.06,33.24)	0.787(0.761,0.814)	<0.001
Central African Republic	20.77 (14.67,29.52)	22.41 (15.6,31.42)	0.271(0.212,0.329)	0	531.39 (364.17,777.43)	575.39 (392.54,824.48)	0.284(0.217,0.35)	<0.001	4.63 (1.99,9.89)	5.02 (2.15,10.67)	0.291(0.218,0.365)	<0.001
Chad	13.77 (9.68,19.5	20.44 (14.31,28.	1.296(1.179,1.413)	0	339.92 (233.39,487.	517.4 (356.47,746.	1.372(1.249,1.494)	<0.001	2.89 (1.24,6)	4.41 (1.91,9.39	1.373(1.275,1.472)	<0.001

	8)	68)			64)	8)				)		
Chile	48.73 (34.13,70.44)	84.21 (59.19,121.13)	1.781(1.758,1.805)	0	1233.67 (858.83,1787.18)	2126.29 (1491.17,3082.69)	1.77(1.745,1.795)	<0.001	10.92 (4.73,22.46)	18.75 (8.02,38.32)	1.753(1.717,1.79)	<0.001
China	31.62 (22.52,44.15)	58.61 (41.19,82.36)	2.034(1.921,2.148)	0	832.95 (590.98,1169.17)	1544.17 (1081.83,2169.82)	2.034(1.916,2.153)	<0.001	7.13 (3.08,14.86)	13.34 (5.88,27.55)	2.062(1.947,2.178)	<0.001
Colombia	66.14 (44.55,96.83)	86.7 (59.59,127.64)	0.892(0.853,0.932)	0	1671.46 (1119.62,2409.5)	2190.96 (1493.43,3183.33)	0.89(0.81,0.969)	<0.001	14.9 (6.39,31.36)	19.45 (8.41,41.39)	0.897(0.845,0.949)	<0.001
Comoros	25.33 (17.66,36.38)	29.89 (20.52,42.21)	0.559(0.459,0.659)	0	649.44 (445.6,947.31)	771.62 (529.54,1112.21)	0.577(0.484,0.67)	<0.001	5.75 (2.38,12.06)	6.86 (2.96,14.2)	0.589(0.483,0.695)	<0.001
Congo	22.32 (15.62,31.72)	30.79 (21.28,43.98)	1.056(0.991,1.121)	0	569.57 (392.21,818.16)	794.25 (548.1,1139.69)	1.089(1.019,1.159)	<0.001	4.87 (2.04,9.58)	6.79 (2.99,14.29)	1.081(1.01,1.152)	<0.001
Cook Islands	80.62 (54.64,116.39)	120.25 (82.98,174.65)	1.276(1.206,1.345)	0	2079.73 (1435.05,3015.09)	3094.71 (2112.97,4501.41)	1.266(1.187,1.345)	<0.001	18.23 (8.07,38.52)	27.02 (11.92,58.02)	1.253(1.169,1.337)	<0.001
Costa Rica	80.05 (54.28,118.8)	106.18 (71.68,151.78)	0.923(0.891,0.955)	0	2072.61 (1414.64,3000.01)	2742.27 (1860.44,3961.65)	0.916(0.875,0.957)	<0.001	18.09 (8.12,37.61)	23.96 (10.36,52.06)	0.911(0.898,0.924)	<0.001
Coted'Ivoire	18.52 (12.78,26.58)	29.82 (20.56,43.32)	1.56(1.504,1.617)	0	466.87 (319.36,681.94)	769.68 (528.59,1131.62)	1.645(1.587,1.703)	<0.001	4 (1.67,8.65)	6.63 (2.89,14.01)	1.659(1.6,1.719)	<0.001
Croatia	6.24	8.37	0.974(0.9,1.	0	157.09	211.96	0.99(0.919,1	<0.0	1.37	1.85	1.011(0.919,	<0.0

	(4.15,9.44)	(5.6,12.16)	048)		(101.76,240.28)	(139.84,319.03)	.06)	01	(0.58,2.94)	(0.75,3.94)	1.102)	01
Cuba	49.87 (33.56,70.8)	66.06 (44.66,92.86)	0.915(0.874,0.956)	0	1294.89 (869.23,1852.94)	1720.78 (1167.5,2445.64)	0.921(0.889,0.954)	<0.001	11.42 (4.92,23.71)	15.18 (6.57,31.88)	0.924(0.874,0.974)	<0.001
Cyprus	89.38 (61.72,125.61)	130.16 (89.94,187.01)	1.18(1.072,1.289)	0	2292.82 (1594.76,3235.1)	3316.4 (2294.61,4779.62)	1.169(1.102,1.236)	<0.001	20.69 (9.21,42.26)	29.81 (13.38,61.05)	1.147(1.05,1.245)	<0.001
Czechia	6.4 (4.14,9.49)	8.17 (5.52,12.08)	0.818(0.762,0.874)	0	162.01 (103.51,240.99)	207.67 (136.98,311.84)	0.814(0.767,0.86)	<0.001	1.41 (0.57,3.01)	1.8 (0.75,3.78)	0.812(0.721,0.903)	<0.001
Democratic People's Republic of Korea	34.19 (24.4,48.54)	38.25 (26.36,53.31)	0.363(0.351,0.375)	0	874.92 (614.67,1268.56)	980.6 (674.56,1378.28)	0.366(0.346,0.386)	<0.001	7.57 (3.18,15.9)	8.48 (3.64,17.74)	0.364(0.316,0.412)	<0.001
Democratic Republic of the Congo	17.28 (12.13,24.51)	24.89 (17.44,35.27)	1.25(1.123,1.377)	0	435.63 (300.69,634.61)	635.93 (437.9,918.11)	1.285(1.155,1.415)	<0.001	3.74 (1.57,7.77)	5.49 (2.38,11.37)	1.299(1.149,1.45)	<0.001
Denmark	104.99 (71.35,150.67)	132.29 (90.98,188.09)	0.75(0.739,0.761)	0	2544.93 (1771.22,3562.47)	3274.15 (2274.55,4525.94)	0.817(0.8,0.833)	<0.001	23.04 (10.56,46.51)	29.59 (13.5,60.47)	0.816(0.795,0.838)	<0.001
Djibouti	20.2 (14.29,28.	33.73 (22.91,49.	1.69(1.619,1.761)	0	513.82 (355.15,762.	875.22 (590.56,1273	1.759(1.678,1.841)	<0.001	4.44 (1.87,9.34	7.58 (3.33,16.2	1.765(1.683,1.847)	<0.001

	93)	09)			5)	.9)			)	2)		
Dominica	45.28 (30.78,66)	62.11 (41.74,88.32)	1.031(0.956,1.105)	0	1176.15 (793.43,1687.7)	1614.82 (1087.5,2326.9)	1.026(0.954,1.098)	<0.001	10.42 (4.45,21.62)	14.28 (6.26,30)	1.02(0.954,1.087)	<0.001
Dominican Republic	40.08 (27.3,57.39)	63.06 (42.23,90)	1.473(1.424,1.523)	0	1027.9 (701.99,1483.43)	1628.37 (1096.59,2382.98)	1.502(1.446,1.558)	<0.001	9.19 (4.01,18.76)	14.3 (6.06,29.63)	1.428(1.359,1.496)	<0.001
Ecuador	114.4 (79.03,161.06)	151.32 (105.93,208.83)	0.905(0.869,0.942)	0	2827.8 (1969.41,3967.57)	3731.44 (2625.38,5158.52)	0.908(0.862,0.954)	<0.001	24.55 (11,51.85)	32.36 (14.2,67.26)	0.901(0.863,0.94)	<0.001
Egypt	69 (47.37,98.84)	87.73 (61.35,124.82)	0.804(0.745,0.862)	0	1820.58 (1239.38,2595.06)	2307.93 (1600.55,3266.64)	0.775(0.717,0.833)	<0.001	16.63 (7.3,35.04)	20.62 (9.17,42.88)	0.712(0.651,0.774)	<0.001
El Salvador	66.04 (44.5,97.91)	94.25 (63.08,136.91)	1.155(1.117,1.192)	0	1691.6 (1143.78,2473.81)	2401.53 (1646.79,3446.62)	1.141(1.097,1.185)	<0.001	14.88 (6.45,31.36)	20.9 (9.13,43.82)	1.109(1.071,1.146)	<0.001
Equatorial Guinea	18.36 (12.99,26.31)	41.85 (28.77,59.15)	2.703(2.535,2.871)	0	465.91 (322.91,681.45)	1093.3 (742.52,1576.3)	2.8(2.601,2.999)	<0.001	3.99 (1.7,8.52)	9.41 (4.12,19.17)	2.821(2.621,3.022)	<0.001
Eritrea	15.42 (10.84,21.36)	22.62 (15.9,32.39)	1.266(1.148,1.383)	0	384.65 (262.31,548.86)	577.34 (393.92,836.34)	1.357(1.255,1.459)	<0.001	3.36 (1.45,7.14)	5.04 (2.1,10.68)	1.353(1.221,1.485)	<0.001
Estonia	8.59 (6.01,12.24)	11.98 (8.26,17.46)	1.126(1.077,1.176)	0	213.33 (143.63,309.74)	301.92 (202.68,453.96)	1.175(1.127,1.223)	<0.001	1.9 (0.76,3.96)	2.68 (1.11,5.9)	1.165(1.101,1.23)	<0.001
Eswatini	35.63	44.09	0.726(0.602,	0	905.27	1123.36	0.732(0.6,0.	<0.0	7.95	9.78	0.693(0.576,	<0.0

	(24.62,50.96)	(30.37,62.02)	0.851)		(619.73,1310.38)	(768,1598.3)	865)	01	(3.36,16.46)	(4.11,19.83)	0.811)	01
Ethiopia	16.08 (11.54,22.37)	23.6 (16.74,33.05)	1.259(1.216,1.302)	0	390.46 (273.42,554.7)	595.16 (421.96,860.31)	1.385(1.345,1.426)	<0.001	3.39 (1.44,7.03)	5.18 (2.21,11.22)	1.391(1.356,1.425)	<0.001
Fiji	61.8 (43.03,89.04)	94.66 (65.78,135.6)	1.37(1.324,1.416)	0	1605.55 (1105,2317.44)	2456.86 (1705.88,3525.51)	1.367(1.313,1.421)	<0.001	14.17 (6.24,28.85)	21.64 (9.6,44.18)	1.363(1.301,1.424)	<0.001
Finland	105.15 (71.1,151.56)	131.64 (90.39,188.24)	0.728(0.708,0.747)	0	2626.78 (1815.63,3706.76)	3328.88 (2300.34,4678.11)	0.768(0.756,0.78)	<0.001	23.6 (10.51,49.06)	29.88 (13.55,61.35)	0.763(0.738,0.788)	<0.001
France	97.69 (67.45,140.16)	122.95 (84.55,174.44)	0.74(0.723,0.758)	0	2522.94 (1719.41,3590.99)	3174.8 (2171.54,4513.88)	0.741(0.733,0.75)	<0.001	23.1 (10.34,48.15)	28.86 (13.07,59.63)	0.72(0.699,0.74)	<0.001
Gabon	24.92 (17.47,35.43)	37.2 (25.29,54.15)	1.32(1.243,1.397)	0	644.26 (444.31,926.07)	972.75 (645.02,1427.42)	1.356(1.268,1.444)	<0.001	5.55 (2.37,11.6)	8.36 (3.54,17.77)	1.346(1.26,1.432)	<0.001
Gambia	18.62 (13.1,25.81)	27.37 (19.17,38.79)	1.266(1.135,1.396)	0	469.83 (325.81,671.72)	702.01 (485.66,1015.59)	1.322(1.211,1.434)	<0.001	4.05 (1.7,8.55)	6.05 (2.56,12.22)	1.313(1.212,1.414)	<0.001
Georgia	16.12 (10.92,23.06)	28.18 (19.41,38.83)	1.845(1.777,1.913)	0	410.16 (271.31,599.43)	723.25 (493.76,1013.69)	1.873(1.807,1.939)	<0.001	3.6 (1.44,7.66)	6.34 (2.74,13.2)	1.869(1.787,1.95)	<0.001
Germany	99.39 (69.44,138.47)	123.43 (85.89,173.38)	0.685(0.613,0.757)	0	2467.39 (1744.88,3387.74)	3088.44 (2154.31,4291.35)	0.711(0.667,0.755)	<0.001	22.31 (10.32,46.48)	27.54 (12.63,55.81)	0.677(0.653,0.701)	<0.001

Ghana	19.32 (13.57,27.07)	28.4 (20.22,40.84)	1.257(1.184,1.331)	0	486.05 (337.32,700.95)	728.65 (505.44,1058.76)	1.323(1.201,1.444)	<0.001	4.18 (1.81,8.99)	6.26 (2.7,12.8)	1.313(1.214,1.413)	<0.001
Greece	112.92 (77.51,160.11)	142.85 (96.28,203.73)	0.746(0.705,0.788)	0	2887.01 (1978.15,4111.63)	3616.57 (2488.92,5139.19)	0.714(0.68,0.747)	<0.001	25.89 (11.51,53.11)	32.31 (14.56,68.03)	0.703(0.667,0.739)	<0.001
Greenland	49.05 (34.69,68.9)	65.26 (46.18,93.26)	0.924(0.897,0.952)	0	1227.79 (853.57,1725.29)	1629.81 (1146.26,2310.59)	0.91(0.859,0.96)	<0.001	10.97 (4.78,22.49)	14.55 (6.51,31.04)	0.906(0.845,0.968)	<0.001
Grenada	39.13 (26.59,55.64)	55.96 (37.72,78.63)	1.18(1.065,1.294)	0	1012.47 (686.36,1452)	1453.75 (974.11,2070.27)	1.189(1.082,1.297)	<0.001	8.96 (3.86,18.58)	12.85 (5.78,27.57)	1.185(1.069,1.302)	<0.001
Guam	86.32 (58.77,124.28)	121.65 (83.72,171.37)	1.102(1.059,1.144)	0	2229.17 (1532.6,3173.87)	3133.82 (2156.98,4475.7)	1.095(1.05,1.14)	<0.001	19.56 (8.5,40.64)	27.47 (12.13,57.31)	1.089(1.048,1.131)	<0.001
Guatemala	56.21 (37.96,80.7)	80.2 (53.7,116.27)	1.146(1.101,1.19)	0	1441.02 (977.12,2090.17)	2065.87 (1361.35,3003.97)	1.146(1.092,1.2)	<0.001	12.4 (5.29,26.42)	17.7 (7.62,36.65)	1.149(1.065,1.233)	<0.001
Guinea	16.92 (11.8,23.47)	25.26 (17.73,35.88)	1.312(1.239,1.386)	0	424.68 (293.62,604.59)	644.69 (446.28,924.09)	1.361(1.29,1.433)	<0.001	3.63 (1.54,7.38)	5.53 (2.38,11.99)	1.389(1.283,1.495)	<0.001
Guinea-Bissau	17 (11.9,24.38)	25.19 (17.47,35.6)	1.289(1.104,1.475)	0	425.17 (295.81,627.82)	643.63 (442.85,927.29)	1.362(1.195,1.53)	<0.001	3.66 (1.54,7.63)	5.56 (2.39,11.56)	1.358(1.244,1.474)	<0.001
Guyana	40.25 (27.22,58.	58.12 (39.35,81.	1.224(1.174,1.274)	0	1047.04 (706.71,1541	1517.95 (1022.21,216	1.243(1.19,1.296)	<0.001	9.22 (3.95,19.7	13.32 (5.77,28.5	1.229(1.147,1.311)	<0.001

	03)	73)			)	9.77)			8)	)		
Haiti	30.65 (20.52,44.73)	38.13 (26.31,54.71)	0.746(0.699,0.794)	0	788.95 (524.28,1148.73)	976.99 (669.63,1376.63)	0.731(0.685,0.777)	<0.001	6.95 (3.06,15)	8.42 (3.65,17.67)	0.667(0.619,0.714)	<0.001
Honduras	53.72 (36.58,78.36)	81.11 (54.85,116.84)	1.338(1.309,1.367)	0	1385.67 (938.08,2027.87)	2086.66 (1429.96,3031.72)	1.328(1.282,1.374)	<0.001	12.02 (5.19,25.84)	17.99 (7.91,38.23)	1.309(1.254,1.365)	<0.001
Hungary	6.91 (4.51,9.99)	8.5 (5.68,12.68)	0.681(0.66,0.702)	0	174.62 (110.61,261.41)	215.28 (141.05,329.6)	0.686(0.653,0.719)	<0.001	1.52 (0.65,3.29)	1.87 (0.78,3.93)	0.692(0.597,0.786)	<0.001
Iceland	116.71 (80.64,166.97)	146.71 (100.65,205.53)	0.742(0.708,0.775)	0	2981.12 (2066.51,4193.15)	3733.33 (2607.91,5311.92)	0.727(0.695,0.76)	<0.001	26.78 (12.25,55.56)	33.43 (15.1,68.93)	0.715(0.676,0.754)	<0.001
India	26.95 (19.55,37.06)	46.93 (33.28,64.95)	1.85(1.804,1.895)	0	679.25 (489,938.81)	1269.76 (896.84,1778.02)	2.082(2.009,2.155)	<0.001	6 (2.64,12.71)	11.07 (4.86,23.2)	2.008(1.972,2.045)	<0.001
Indonesia	58.17 (40.8,80.27)	111.3 (78.67,155.47)	2.131(2.073,2.189)	0	1499.36 (1061.71,2116.89)	2876.58 (2028.48,4056.86)	2.138(2.097,2.18)	<0.001	13.3 (5.84,27.16)	25.38 (11.36,52.45)	2.119(2.087,2.152)	<0.001
Iran (Islamic Republic of)	63.81 (44.36,89.88)	84.97 (59.63,120.59)	0.956(0.88,1.032)	0	1670.73 (1158.19,2352.29)	2218.89 (1544.61,3143.54)	0.954(0.876,1.032)	<0.001	15.33 (6.79,32.2)	20.33 (9.07,42.81)	0.946(0.789,1.103)	<0.001
Iraq	67.32 (46.8,96.79)	77.47 (53.29,110.53)	0.489(0.412,0.565)	0	1766.26 (1226.68,2552.35)	2031.04 (1375.42,2881.58)	0.485(0.402,0.568)	<0.001	15.58 (6.72,32.44)	17.84 (7.89,36.82)	0.459(0.408,0.51)	<0.001

Ireland	109.11 (73.79,154.14)	135.89 (95.25,194.96)	0.713(0.691,0.735)	0	2782.52 (1917.65,3985.77)	3456.86 (2412.02,4875.75)	0.708(0.687,0.729)	<0.001	24.98 (10.96,51.69)	30.91 (13.88,63.01)	0.692(0.664,0.721)	<0.001
Israel	96.25 (66.52,137.62)	124.98 (85.87,176.13)	0.843(0.801,0.884)	0	2434.98 (1697.02,3500.33)	3154.91 (2193.73,4470.66)	0.834(0.801,0.866)	<0.001	21.83 (9.77,45.24)	28.12 (12.55,58.11)	0.816(0.782,0.85)	<0.001
Italy	361.55 (250.68,514.27)	326.18 (227.34,458.58)	-0.341(-0.409,-0.274)	0	8559.23 (6129.29,11790.13)	8113.16 (5757.74,11265.85)	-0.175(-0.222,-0.128)	<0.001	74.94 (33.82,158.92)	71.69 (32.16,152.77)	-0.146(-0.198,-0.094)	<0.001
Jamaica	43.86 (29.7,61.25)	58.91 (39.66,82.96)	0.966(0.908,1.023)	0	1142.29 (771.08,1615.72)	1537.25 (1041.9,2211.88)	0.972(0.912,1.032)	<0.001	10.12 (4.38,21.22)	13.6 (5.83,28.88)	0.961(0.87,1.052)	<0.001
Japan	245.52 (175.82,342.02)	266.51 (189.72,375.55)	0.264(0.196,0.332)	0	5930.96 (4259.12,8254.48)	6334.11 (4579.73,8798.66)	0.203(0.155,0.252)	<0.001	51.73 (22.91,104.01)	55.14 (25.1,111.98)	0.198(0.131,0.265)	<0.001
Jordan	62.94 (43.71,88.79)	79.57 (54.97,112.66)	0.816(0.754,0.878)	0	1640.37 (1137.13,2336.56)	2079.34 (1454.02,2938.43)	0.789(0.765,0.813)	<0.001	14.73 (6.37,30.72)	18.36 (7.94,38.84)	0.71(0.615,0.805)	<0.001
Kazakhstan	14.5 (9.88,21.01)	20.32 (13.97,28.66)	1.09(1.032,1.147)	0	368.26 (249.86,541.9)	519.72 (349.98,738.04)	1.117(1.075,1.16)	<0.001	3.26 (1.39,6.99)	4.56 (1.97,9.67)	1.085(1.027,1.143)	<0.001
Kenya	24.02 (17.28,33.52)	28.93 (20.71,40.66)	0.616(0.55,0.681)	0	600.15 (424.71,856.92)	745.75 (525.02,1066.22)	0.73(0.574,0.887)	<0.001	5.2 (2.23,11.05)	6.42 (2.75,13.58)	0.708(0.571,0.845)	<0.001
Kiribati	53.16 (36.63,73.	78.42 (55.09,110	1.268(1.199,1.337)	0	1368.14 (925.82,1945	2019.01 (1411.32,283	1.274(1.209,1.34)	<0.001	11.94 (5.26,25.3	17.64 (7.79,36.5	1.276(1.213,1.34)	<0.001

	86)	.62)			.68)	9.09)			5)	4)		
Kuwait	85.47 (59.03,122.9)	103.15 (71.59,147.51)	0.661(0.609,0.713)	0	2248.21 (1536.04,3220.09)	2710.67 (1867.51,3844.2)	0.643(0.587,0.699)	<0.001	19.88 (8.75,42.07)	23.93 (10.5,49.37)	0.624(0.536,0.712)	<0.001
Kyrgyzstan	13.35 (9.27,18.85)	15.96 (10.75,22.84)	0.59(0.534,0.645)	0	338.67 (230.07,489.15)	406.84 (272.05,592.79)	0.605(0.561,0.649)	<0.001	2.95 (1.24,6.29)	3.55 (1.48,7.69)	0.603(0.541,0.665)	<0.001
Lao People's Democratic Republic	51.33 (35.64,72.65)	95.13 (67.72,133.06)	2.027(1.965,2.09)	0	1307.3 (916.42,1836.9)	2434.47 (1718.64,3501.88)	2.033(1.967,2.099)	<0.001	11.53 (5.12,23.59)	21.43 (9.6,44.1)	2.027(1.974,2.079)	<0.001
Latvia	8.54 (5.93,12.53)	11.05 (7.72,15.8)	0.84(0.819,0.861)	0	211.55 (142.74,323.62)	277.56 (189.97,403.99)	0.883(0.861,0.904)	<0.001	1.88 (0.78,4.08)	2.44 (1.02,5.21)	0.848(0.771,0.925)	<0.001
Lebanon	67.24 (46.94,95.9)	88.4 (61.19,125.89)	0.896(0.861,0.93)	0	1772.91 (1219.44,2557.32)	2333.51 (1616.6,3359.94)	0.922(0.879,0.965)	<0.001	15.49 (6.75,31.54)	20.34 (8.84,41.92)	0.907(0.876,0.938)	<0.001
Lesotho	24.55 (17.16,35.09)	36.43 (25.21,52.58)	1.314(1.233,1.395)	0	624.98 (433.34,898.78)	934.81 (634.27,1354.64)	1.339(1.247,1.432)	<0.001	5.46 (2.37,11.53)	8.08 (3.52,17.79)	1.314(1.212,1.417)	<0.001
Liberia	19.21 (13.55,27.36)	27.66 (19.31,39.77)	1.204(1.104,1.304)	0	486.12 (334.17,708.53)	711.82 (489.58,1042.57)	1.254(1.148,1.359)	<0.001	4.17 (1.77,8.93)	6.03 (2.6,12.44)	1.23(1.071,1.389)	<0.001
Libya	76.75 (52.63,109	84.61 (58,121.75	0.35(0.299,0.402)	0	2004.97 (1363.27,287	2217.09 (1533.97,317	0.36(0.306,0.415)	<0.001	17.78 (7.79,36.3	19.61 (8.52,39.9	0.33(0.297,0.364)	<0.001

	.84)	)			6.03)	7.09)			9)	9)		
Lithuania	7.78 (5.2,11.35)	10.68 (7.48,15.6)	1.032(1.012,1.052)	0	192.4 (126.52,284.54)	267.43 (184.63,401.79)	1.078(1.045,1.111)	<0.001	1.71 (0.69,3.58)	2.34 (0.95,5.02)	1.046(0.977,1.115)	<0.001
Luxembourg	110.81 (74.77,158.45)	142.81 (98.04,203.2)	0.829(0.809,0.848)	0	2824.46 (1930.64,3999.6)	3617.44 (2530.86,5086.89)	0.804(0.784,0.824)	<0.001	25.37 (11.33,52.03)	32.39 (14.42,67.35)	0.793(0.771,0.815)	<0.001
Madagascar	19.08 (13.5,26.9)	21.82 (15.25,31.05)	0.469(0.388,0.55)	0	480.36 (331.6,693.22)	555.02 (380.82,810.24)	0.5(0.424,0.576)	<0.001	4.2 (1.81,9.06)	4.89 (2.09,9.97)	0.513(0.437,0.589)	<0.001
Malawi	25.28 (17.55,36.25)	28.76 (20.26,41.04)	0.44(0.339,0.541)	0	646.77 (441.65,938.9)	720.04 (497.66,1039.89)	0.367(0.266,0.467)	<0.001	5.52 (2.33,11.95)	6.15 (2.71,12.74)	0.379(0.3,0.458)	<0.001
Malaysia	93.46 (65.99,132.7)	161.5 (112.86,226.19)	1.793(1.683,1.903)	0	2395.22 (1691.73,3456.2)	4125.99 (2878.52,5885.36)	1.784(1.693,1.874)	<0.001	20.75 (8.91,41.81)	35.66 (15.84,72.57)	1.762(1.657,1.868)	<0.001
Maldives	54.46 (37.79,75.72)	128.78 (89.7,183.56)	2.824(2.689,2.959)	0	1414.17 (981.78,1979.59)	3297.11 (2261.38,4751.03)	2.766(2.636,2.897)	<0.001	12.51 (5.41,25.61)	29.2 (12.85,63.36)	2.763(2.645,2.881)	<0.001
Mali	14.63 (10.39,20.62)	22.87 (15.87,32.43)	1.467(1.367,1.568)	0	362.79 (250.43,519.38)	579.36 (394.12,836.53)	1.534(1.454,1.614)	<0.001	3.16 (1.36,6.88)	4.99 (2.18,10.79)	1.496(1.392,1.599)	<0.001
Malta	103.7 (72.51,146.94)	141.4 (95.89,201.87)	1.008(0.972,1.045)	0	2654.36 (1833.01,3784.69)	3600.05 (2508.73,5102.58)	0.997(0.968,1.025)	<0.001	23.9 (10.65,47.79)	32.21 (14.45,65.51)	0.973(0.927,1.019)	<0.001
Marshall	45.42	76.66	1.687(1.606,	0	1167.2	1974.44	1.695(1.602,	<0.0	10.22	17.24	1.686(1.582,	<0.0

Islands	(31.36,64.73)	(53.65,106.94)	1.767)		(801.76,1680.51)	(1363.98,2758.18)	1.788)	01	(4.53,21.51)	(7.5,36.11)	1.791)	01
Mauritania	23.53 (16.59,34.12)	32.51 (22.66,46.17)	1.068(0.975,1.161)	0	596.35 (409.12,882.89)	840.8 (576.26,1205.45)	1.131(1.041,1.221)	<0.001	5.21 (2.25,11.3)	7.34 (3.15,14.94)	1.129(1.029,1.229)	<0.001
Mauritius	91.41 (65.12,130.37)	147.1 (103.43,208.27)	1.56(1.468,1.651)	0	2339.78 (1639.26,3402.77)	3769.91 (2642.56,5384.13)	1.566(1.538,1.594)	<0.001	20.49 (9.2,41.69)	32.96 (14.5,69.47)	1.561(1.531,1.59)	<0.001
Mexico	142.27 (97.55,201.58)	141.45 (99.1,197.25)	-0.063(-0.206,0.08)	0.388	3593 (2488.62,4991.27)	3622.47 (2553.1,5009.74)	0.008(-0.134,0.15)	0.914	31.28 (13.89,65.38)	31.26 (13.85,66.05)	-0.024(-0.225,0.177)	0.813
Micronesia (Federated States of)	58.29 (40.79,83.06)	84.43 (59.11,119.36)	1.149(1.074,1.224)	0	1500.49 (1041.3,2118.6)	2173.82 (1520.35,3095.74)	1.158(1.09,1.226)	<0.001	13.16 (5.86,27.34)	19.04 (8.5,39.28)	1.148(1.07,1.226)	<0.001
Monaco	121.29 (84.08,171.57)	143.38 (98.27,204.74)	0.534(0.519,0.55)	0	3091.82 (2103.11,4432.41)	3641.66 (2511.44,5143.38)	0.52(0.495,0.546)	<0.001	27.81 (12.28,58.26)	32.58 (14.67,68.3)	0.496(0.467,0.525)	<0.001
Mongolia	12.82 (8.81,18.05)	18.08 (12.37,25.9)	1.143(1.087,1.199)	0	322.94 (215.88,460.86)	460.34 (310.36,656.75)	1.177(1.113,1.24)	<0.001	2.82 (1.21,5.92)	4.04 (1.69,8.81)	1.184(1.119,1.248)	<0.001
Montenegro	6.69 (4.43,9.94)	8.54 (5.77,12.6)	0.818(0.783,0.854)	0	168.82 (109.77,256.02)	216.34 (141.41,323.08)	0.827(0.787,0.866)	<0.001	1.47 (0.63,3.12)	1.89 (0.76,4.03)	0.826(0.768,0.883)	<0.001
Morocco	58.93	77.28	0.929(0.895,	0	1524.67	2030.18	0.972(0.9,1.	<0.0	13.8	18.05	0.894(0.848,	<0.0

	(40.56,83.27)	(54.07,111.57)	0.963)		(1037.99,2170.91)	(1397.84,2905.2)	043)	01	(6.1,28.55)	(8.04,37.17)	0.94)	01
Mozambique	18.61 (13.01,26.07)	27.65 (19.07,39.32)	1.293(1.265,1.32)	0	470.1 (321.11,685.54)	711.86 (483.64,1033.43)	1.354(1.316,1.393)	<0.001	4.03 (1.7,7.97)	6.07 (2.57,12.45)	1.336(1.289,1.384)	<0.001
Myanmar	47.4 (33,67.47)	97.89 (69.86,137.51)	2.403(2.331,2.476)	0	1205.63 (838.29,1732.92)	2487.69 (1744.39,3493.6)	2.398(2.326,2.471)	<0.001	10.63 (4.68,22.3)	21.97 (9.74,45.39)	2.411(2.331,2.491)	<0.001
Namibia	26.25 (18.52,36.87)	34.88 (24.46,49.9)	0.962(0.896,1.028)	0	660.12 (456.37,927.9)	885.34 (609.78,1270.79)	0.972(0.935,1.01)	<0.001	5.73 (2.42,12.32)	7.69 (3.41,15.66)	0.965(0.88,1.051)	<0.001
Nauru	67.64 (46.69,96.07)	98.26 (68.62,140.24)	1.182(1.104,1.259)	0	1744.98 (1208.58,2499.65)	2531.5 (1764.66,3680.18)	1.176(1.102,1.25)	<0.001	15.25 (6.79,32.17)	22.1 (9.74,45.92)	1.185(1.108,1.262)	<0.001
Nepal	15.43 (11.02,21.57)	26.39 (18.33,36.92)	1.756(1.7,1.813)	0	384.67 (266.18,550.92)	655.33 (450.84,937.3)	1.744(1.684,1.803)	<0.001	3.39 (1.44,7.33)	5.86 (2.48,12.16)	1.784(1.718,1.85)	<0.001
Netherlands	97.46 (66.35,137.9)	123.5 (85.5,177.57)	0.757(0.744,0.77)	0	2491.91 (1701.29,3511.06)	3147.42 (2172.38,4452.61)	0.747(0.734,0.76)	<0.001	22.48 (10.04,46.05)	28.23 (12.41,58.9)	0.719(0.686,0.752)	<0.001
New Zealand	236.74 (164.69,338.4)	236.5 (167.69,326.85)	-0.01(-0.038,0.018)	0.497	5616.24 (3953.22,7885.09)	5689.13 (4094.5,7762.63)	0.039(0.019,0.058)	<0.001	48.86 (21.76,100.15)	49.57 (22.7,103.17)	0.043(0.027,0.06)	<0.001
Nicaragua	61.65 (41.12,90.54)	87.88 (58.85,127.37)	1.145(1.106,1.184)	0	1575.26 (1059.42,2304.09)	2246.36 (1501.49,3214.56)	1.145(1.113,1.177)	<0.001	13.74 (5.89,28.78)	19.53 (8.54,42.28)	1.135(1.096,1.174)	<0.001

Niger	15.23 (10.9,21.01)	20.38 (14.43,28.8)	0.954(0.855, 1.054)	0	381.8 (265.36,538.1)	517.15 (357.86,748.6)	0.998(0.878, 1.118)	<0.001	3.34 (1.43,6.98)	4.47 (1.88,9.34)	0.952(0.808, 1.096)	<0.001
Nigeria	21.57 (15.6,29.9)	28.39 (20.27,39.84)	0.905(0.796, 1.013)	0	540.92 (382.38,778.28)	739.31 (518.44,1052.63)	1.028(0.912, 1.144)	<0.001	4.64 (2,9.74)	6.39 (2.75,13.68)	1.058(0.943, 1.173)	<0.001
Niue	73.71 (49.99,104.45)	112.47 (78.48,161.45)	1.363(1.329, 1.396)	0	1903.64 (1296.57,2712.76)	2895.05 (2019.02,4151.71)	1.356(1.32,1.392)	<0.001	16.69 (7.3,34.62)	25.32 (11.3,52.79)	1.348(1.308, 1.388)	<0.001
North Macedonia	5.63 (3.8,8.5)	7.77 (5.16,11.52)	1.059(1.038, 1.079)	0	141.64 (92.27,219.15)	196.09 (127.73,299.07)	1.071(1.048, 1.095)	<0.001	1.24 (0.49,2.75)	1.71 (0.73,3.62)	1.061(0.994, 1.128)	<0.001
Northern Mariana Islands	82.43 (56.28,117.27)	106.78 (74.03,150.86)	0.836(0.821, 0.852)	0	2127.89 (1465.89,3008.36)	2752.4 (1891.74,3874.16)	0.836(0.793, 0.878)	<0.001	18.66 (8.28,39.85)	24.12 (10.51,51.12)	0.831(0.781, 0.882)	<0.001
Norway	109.14 (76.47,153.31)	125.2 (88.34,177.42)	0.452(0.405, 0.499)	0	2807.05 (1957.05,3960.95)	3217.26 (2259.05,4591.03)	0.442(0.404, 0.481)	<0.001	25.18 (11.32,52.12)	28.8 (12.89,59.17)	0.437(0.404, 0.469)	<0.001
Oman	55.56 (37.72,79.05)	91.72 (63.06,129.69)	1.648(1.622, 1.674)	0	1453.82 (980.78,2101.72)	2410.09 (1651.57,3437.57)	1.681(1.622, 1.74)	<0.001	12.81 (5.59,26.63)	21.2 (9.25,44.09)	1.657(1.608, 1.706)	<0.001
Pakistan	26.15 (18.92,36.52)	30.39 (21.44,42.85)	0.497(0.438, 0.556)	0	656.67 (463.86,945.5)	765.75 (526.76,1091.9)	0.553(0.338, 0.768)	<0.001	5.84 (2.5,12.49)	6.89 (2.97,14.96)	0.581(0.368, 0.796)	<0.001
Palau	77.39 (53,111.83	112.01 (76.55,161	1.182(1.162, 1.203)	0	1997.33 (1383.37,284	2887.7 (1990.57,412	1.178(1.105, 1.252)	<0.001	17.48 (7.66,37.1	25.24 (11.1,51.3	1.176(1.138, 1.214)	<0.001

	)	.1)			9.89)	6.06)			2)	7)		
Palestine	58.43 (40,83.26)	73.97 (50.93,103.68)	0.76(0.684,0.836)	0	1530.73 (1029.05,2236.15)	1942.17 (1327.01,2740.61)	0.77(0.698,0.842)	<0.001	13.52 (5.7,28.21)	17.1 (7.73,36.17)	0.767(0.704,0.83)	<0.001
Panama	56.21 (37.96,80.7)	90.09 (61.25,131.72)	1.536(1.495,1.577)	0	1455.4 (982.73,2122.06)	2331.37 (1607.36,3400.98)	1.535(1.494,1.575)	<0.001	12.59 (5.45,27.39)	20.12 (8.73,43.82)	1.526(1.469,1.584)	<0.001
Papua New Guinea	43.75 (30.16,61.26)	64.43 (44.85,90.65)	1.231(1.166,1.295)	0	1109.48 (759.78,1554.15)	1637.42 (1134.3,2363.63)	1.241(1.168,1.314)	<0.001	9.6 (4.31,20.19)	14.19 (6.16,29.9)	1.248(1.178,1.318)	<0.001
Paraguay	14.97 (10.2,22.09)	23.19 (15.89,33.63)	1.433(1.397,1.47)	0	385.93 (259.62,572.06)	604.4 (409.3,878.16)	1.47(1.431,1.508)	<0.001	3.41 (1.41,7.24)	5.3 (2.25,11.07)	1.444(1.4,1.488)	<0.001
Peru	96.39 (66.02,137.32)	129.39 (87.3,188)	0.968(0.91,1.026)	0	2280.86 (1525.39,3206.98)	3241.41 (2215.89,4687.01)	1.158(1.005,1.311)	<0.001	19.76 (8.67,42.94)	27.92 (12.06,59.4)	1.169(1.052,1.286)	<0.001
Philippines	64.13 (45.13,88.91)	108.84 (77.28,151.59)	1.75(1.668,1.832)	0	1644.28 (1149,2331.76)	2801.89 (1957.64,3975.8)	1.766(1.654,1.879)	<0.001	14.47 (6.23,29.52)	24.85 (10.81,51.3)	1.791(1.683,1.9)	<0.001
Poland	10.17 (6.98,14.9)	10.53 (7.59,14.17)	0.096(0.055,0.137)	0	256.52 (168.78,384.74)	269.48 (190.73,368.48)	0.144(0.109,0.179)	<0.001	2.26 (0.94,4.62)	2.36 (1.05,4.77)	0.122(0.094,0.15)	<0.001
Portugal	93.62 (64.85,132.19)	128.22 (87.3,180.67)	1.018(0.968,1.069)	0	2401.97 (1664.57,3408.13)	3268.08 (2250.48,4623.77)	0.989(0.945,1.034)	<0.001	21.57 (9.61,43.98)	29.25 (13.51,58.84)	0.982(0.942,1.023)	<0.001
Puerto	68.39	87.35	0.794(0.772,	0	1778.93	2271.06	0.795(0.774,	<0.0	15.76	20.05	0.79(0.766,0	<0.0

Rico	(46.04,97.24)	(59.06,125.31)	0.816)		(1200.86,2550.48)	(1510.57,3321.18)	0.817)	01	(6.64,33.39)	(8.88,41.54)	.813)	01
Qatar	85.6 (59.34,123.4)	101.08 (68.65,146.01)	0.561(0.513, 0.608)	0	2248.66 (1549.95,3269.52)	2660.35 (1810.21,3885.75)	0.575(0.533, 0.618)	<0.001	19.84 (8.63,40.76)	23.43 (10.24,48.28)	0.555(0.513, 0.597)	<0.001
Republic of Korea	83.28 (58.61,117.62)	130.61 (91.77,185.36)	1.486(1.384, 1.587)	0	2011.11 (1412.84,2882.32)	3117.94 (2187.29,4453.2)	1.459(1.349, 1.568)	<0.001	17.62 (7.83,36.09)	27.23 (12.49,55.05)	1.445(1.314, 1.577)	<0.001
Republic of Moldova	6.86 (4.65,10.02)	9.9 (6.83,14.06)	1.193(1.147, 1.239)	0	168.37 (111.28,250.76)	247.11 (167.49,364.08)	1.249(1.203, 1.294)	<0.001	1.5 (0.63,3.2)	2.2 (0.91,4.71)	1.242(1.147, 1.337)	<0.001
Romania	5.82 (3.82,8.66)	8.21 (5.55,12.24)	1.165(1.073, 1.258)	0	146.28 (94.26,220.69)	207.94 (136.2,318.3)	1.184(1.103, 1.266)	<0.001	1.28 (0.51,2.67)	1.81 (0.75,3.8)	1.19(1.094,1.286)	<0.001
Russian Federation	8.75 (6.11,12.36)	11.18 (7.86,15.88)	0.796(0.771, 0.822)	0	211.11 (142.86,303.3)	273.16 (189.03,391.57)	0.843(0.818, 0.868)	<0.001	1.87 (0.77,4.05)	2.41 (0.99,5.02)	0.836(0.806, 0.866)	<0.001
Rwanda	22.88 (15.98,32.64)	28.51 (19.76,41.16)	0.746(0.713, 0.779)	0	568.63 (392.21,820.52)	719.98 (494.75,1039.74)	0.77(0.716,0.825)	<0.001	4.96 (2.11,10.48)	6.23 (2.68,13.28)	0.742(0.703, 0.782)	<0.001
Saint Kitts and Nevis	51.86 (35.31,73.46)	70.38 (47.73,102.7)	0.99(0.972,1.009)	0	1347.52 (919.18,1910.24)	1831.33 (1243.87,2655.58)	1(0.958,1.042)	<0.001	11.92 (5.17,24.77)	16.17 (7.2,33.53)	0.989(0.943, 1.034)	<0.001
Saint Lucia	43.13 (29.33,61.09)	57.81 (39.37,82.03)	0.956(0.876, 1.036)	0	1117.02 (755.79,1587.3)	1501.8 (1022.68,2110.44)	0.974(0.877, 1.071)	<0.001	9.88 (4.29,20.73)	13.27 (5.86,27.62)	0.955(0.89,1.021)	<0.001

Saint Vincent and the Grenadines	39.41 (26.42,55.74)	59.34 (39.91,84.1)	1.35(1.278,1.421)	0	1019.92 (684.21,1450.15)	1539.91 (1040.44,2200.11)	1.348(1.316,1.379)	<0.001	9.04 (3.87,19.4)	13.61 (6.01,29.23)	1.355(1.277,1.434)	<0.001
Samoa	69.73 (47.83,101.2)	94.35 (65.04,135.51)	0.976(0.893,1.06)	0	1799.08 (1223.51,2616.2)	2430.46 (1677.51,3464.56)	0.973(0.903,1.043)	<0.001	15.75 (6.76,32.15)	21.25 (9.51,44.65)	0.969(0.899,1.04)	<0.001
San Marino	115.78 (80.13,164.41)	138.58 (94.84,193.99)	0.568(0.521,0.614)	0	2956.09 (2056.04,4220.14)	3519.75 (2397.78,4919.3)	0.554(0.523,0.586)	<0.001	26.61 (12.05,55.38)	31.49 (13.97,65.8)	0.536(0.503,0.569)	<0.001
Sao Tome and Principe	20.33 (14.35,28.92)	29.77 (20.97,42.74)	1.24(1.147,1.334)	0	508.44 (352.94,727.79)	756.38 (522.43,1083.55)	1.29(1.201,1.379)	<0.001	4.38 (1.88,9.48)	6.53 (2.85,13.49)	1.308(1.204,1.412)	<0.001
Saudi Arabia	70.27 (48.23,99.58)	99.3 (67.62,141.76)	1.175(1.131,1.22)	0	1845.04 (1267.78,2628.16)	2608.01 (1796.32,3769.73)	1.184(1.136,1.233)	<0.001	16.26 (7.14,33.65)	22.95 (10.33,48.13)	1.135(1.099,1.172)	<0.001
Senegal	21.56 (15.36,31.07)	27.06 (18.73,38.68)	0.745(0.678,0.811)	0	547.77 (381.45,799.51)	691.99 (476.55,995.18)	0.769(0.692,0.845)	<0.001	4.77 (2.03,10.45)	6.02 (2.62,12.94)	0.77(0.673,0.867)	<0.001
Serbia	5.82 (3.82,8.66)	7.84 (5.25,11.29)	0.987(0.958,1.015)	0	146.42 (94.4,221.26)	198.24 (130.49,289.42)	0.984(0.964,1.005)	<0.001	1.28 (0.52,2.68)	1.73 (0.72,3.68)	0.985(0.895,1.075)	<0.001
Seychelles	97.34 (68.68,138	137.81 (96.61,193	1.138(1.072,1.204)	0	2498.6 (1750.54,357	3523.9 (2459.9,5007	1.121(1.052,1.19)	<0.001	21.97 (9.62,45.0	30.96 (13.71,64.	1.113(1.055,1.171)	<0.001

	.13)	.5)			2.46)	.5)			4)	18)		
Sierra Leone	16.72 (11.65,23.11)	27.7 (19.15,40.03)	1.646(1.573,1.719)	0	419.42 (288.2,599.63)	713.61 (484.16,1047.16)	1.762(1.588,1.937)	<0.001	3.57 (1.57,7.24)	6.08 (2.6,12.95)	1.731(1.555,1.908)	<0.001
Singapore	95.56 (67,136.78)	154.59 (106.48,225.31)	1.559(1.525,1.593)	0	2295.47 (1619.09,3281.98)	3694.7 (2599.4,5295.41)	1.548(1.522,1.574)	<0.001	20.11 (9.06,40.92)	32.24 (14.9,68.63)	1.535(1.478,1.591)	<0.001
Slovakia	6.01 (4.01,8.92)	8.29 (5.71,12.21)	1.042(0.999,1.084)	0	150.96 (96.22,227.33)	209.94 (138.84,314.83)	1.064(1.024,1.104)	<0.001	1.31 (0.53,2.9)	1.83 (0.73,4.19)	1.084(1.002,1.167)	<0.001
Slovenia	6.42 (4.34,9.47)	8.89 (5.95,12.98)	1.079(1.008,1.151)	0	159.85 (104.18,243.3)	223.83 (145.4,331.38)	1.112(1.064,1.16)	<0.001	1.38 (0.57,2.91)	1.92 (0.82,4.06)	1.089(1.014,1.164)	<0.001
Solomon Islands	45.14 (31.32,64.19)	71.08 (48.69,100.38)	1.429(1.366,1.492)	0	1159.64 (791.36,1675.47)	1827.09 (1265.61,2539.07)	1.437(1.372,1.502)	<0.001	10.16 (4.48,21.21)	15.99 (7.1,34.09)	1.439(1.381,1.498)	<0.001
Somalia	18.94 (13.25,26.64)	21.92 (15.42,31.51)	0.469(0.43,0.509)	0	476.9 (329.34,682.32)	556.85 (381.31,821.91)	0.493(0.453,0.533)	<0.001	4.1 (1.75,8.66)	4.82 (2.02,10.22)	0.517(0.482,0.552)	<0.001
South Africa	35.82 (25.09,50.69)	46.64 (32.6,66.11)	0.882(0.793,0.971)	0	919.13 (637,1310.8)	1179.02 (803.6,1670.77)	0.843(0.733,0.952)	<0.001	8.04 (3.45,17.35)	10.22 (4.4,21.26)	0.817(0.694,0.94)	<0.001
South Sudan	20.85 (14.65,29.39)	23.01 (16.04,32.97)	0.337(0.283,0.39)	0	527.51 (363.47,759.55)	586.39 (401.64,846.72)	0.357(0.31,0.404)	<0.001	4.57 (1.89,9.95)	5.09 (2.19,10.82)	0.363(0.297,0.429)	<0.001
Spain	105.68	132.93	0.746(0.726,	0	2604.85	3307.02	0.781(0.765,	<0.0	23.33	29.3	0.746(0.722,	<0.0

	(72.61,147.39)	(91.49,189.51)	0.767)		(1798.29,3664.3)	(2315.93,4612.92)	0.797)	01	(10.71,49.07)	(13.32,61.49)	0.771)	01
Sri Lanka	73.74 (51.72,104.79)	124.05 (87.31,172.3)	1.731(1.644, 1.818)	0	1894.99 (1335.07,2717.99)	3164.18 (2231.82,4418.47)	1.712(1.621, 1.803)	<0.001	16.92 (7.53,35.24)	27.8 (12.32,55.29)	1.635(1.497, 1.773)	<0.001
Sudan	39.11 (26.86,55.01)	68.77 (47.85,96.5)	1.837(1.783, 1.891)	0	1020.97 (687.98,1446.97)	1817.65 (1262.35,2594.65)	1.894(1.799, 1.99)	<0.001	8.89 (3.92,19.29)	15.67 (7.05,32.71)	1.848(1.778, 1.917)	<0.001
Suriname	45.02 (30.63,64.12)	61.38 (41.59,86.89)	1.007(0.956, 1.059)	0	1168.72 (790.65,1650.18)	1595.49 (1084.73,2279.93)	1.017(0.97,1.064)	<0.001	10.32 (4.47,21.66)	14.04 (6.19,30.03)	1.012(0.949, 1.076)	<0.001
Sweden	82.87 (58.14,118.45)	101.12 (70.76,143.7)	0.658(0.607, 0.708)	0	2153.21 (1500.37,3085.94)	2606.76 (1811.07,3683.24)	0.631(0.583, 0.678)	<0.001	19.36 (8.63,40.09)	23.32 (10.52,47.78)	0.611(0.555, 0.668)	<0.001
Switzerland	109.15 (74.78,155.16)	122.96 (85.77,176.87)	0.382(0.368, 0.396)	0	2775.55 (1920.43,3961.33)	3125.63 (2182.43,4408.64)	0.382(0.373, 0.391)	<0.001	24.88 (10.89,50.64)	27.95 (12.46,57)	0.369(0.347, 0.392)	<0.001
Syrian Arab Republic	58.69 (39.67,82.71)	77.22 (53.73,110.46)	0.913(0.876, 0.95)	0	1525.26 (1028.35,2162.87)	2026.43 (1402.83,2878.71)	0.947(0.906, 0.989)	<0.001	13.38 (5.95,27.83)	17.7 (7.85,36.9)	0.931(0.878, 0.985)	<0.001
Taiwan (Province of China)	59.85 (40.47,85.44)	96.73 (69.47,131.02)	1.568(1.531, 1.606)	0	1540.73 (1034.64,2232.94)	2489.94 (1762.39,3400.01)	1.563(1.54,1.586)	<0.001	13.33 (5.76,28.5)	21.51 (9.46,43.18)	1.564(1.509, 1.62)	<0.001
Tajikistan	10.82 (7.52,15.6)	13.91 (9.48,19.9	0.814(0.775, 0.854)	0	270.08 (184.9,397.0	348.14 (231.14,507.	0.825(0.778, 0.872)	<0.001	2.38 (0.99,5.32	3.1 (1.26,6.67	0.859(0.79,0.928)	<0.001

		4)			6)	29)			)	)		
Thailand	69.74 (48.86,98.38)	136.04 (96.2,190.05)	2.212(2.127,2.296)	0	1787.45 (1248.06,2560.4)	3482.2 (2427.75,4950.67)	2.202(2.129,2.275)	<0.001	16.23 (7.13,33.2)	30.98 (13.79,65.46)	2.14(2.044,2.236)	<0.001
Timor-Leste	48.62 (33.99,68.41)	80.27 (57.49,116.88)	1.659(1.6,1.718)	0	1237.8 (863.34,1736.14)	2052.11 (1441.38,2978.82)	1.668(1.609,1.727)	<0.001	10.95 (4.86,22.52)	18.19 (7.98,36.77)	1.675(1.602,1.747)	<0.001
Togo	17.55 (12.32,24.85)	26.21 (18.27,37.77)	1.323(1.225,1.421)	0	437.35 (300.23,626.48)	669.85 (459.08,982.57)	1.394(1.276,1.512)	<0.001	3.79 (1.6,8.14)	5.81 (2.5,12.39)	1.391(1.26,1.522)	<0.001
Tokelau	61.85 (42.9,88.77)	101.55 (70.21,144.88)	1.615(1.551,1.679)	0	1593.51 (1104.38,2283.83)	2616.3 (1803.44,3743.89)	1.616(1.556,1.677)	<0.001	13.96 (6.12,29.21)	22.85 (10.43,47.33)	1.608(1.545,1.672)	<0.001
Tonga	79.12 (54.16,112.92)	108.25 (73.8,156.06)	1.005(0.954,1.055)	0	2041.69 (1398.74,2982.69)	2790.79 (1907.11,3970.77)	0.986(0.925,1.048)	<0.001	17.87 (7.82,36.77)	24.38 (10.65,50.19)	0.981(0.922,1.039)	<0.001
Trinidad and Tobago	45.46 (30.5,64.88)	65.49 (43.65,94.12)	1.209(1.116,1.302)	0	1176.96 (792.17,1656.46)	1708.6 (1152.71,2458)	1.228(1.127,1.33)	<0.001	10.55 (4.57,22.2)	15.08 (6.58,31.19)	1.19(1.086,1.293)	<0.001
Tunisia	56.39 (38.56,82.4)	79.26 (54.87,112.67)	1.118(1.084,1.152)	0	1476.32 (994.13,2177.33)	2082.09 (1439.97,2956.93)	1.141(1.085,1.197)	<0.001	13.43 (5.77,28.09)	18.48 (8.25,38.75)	1.071(1.032,1.111)	<0.001
Turkey	53.53 (37.16,76.19)	74.78 (51.54,106.27)	1.103(0.96,1.247)	0	1393.28 (954.5,2010.92)	1954.56 (1341.83,2795.21)	1.077(0.855,1.298)	<0.001	12.72 (5.69,26.4)	17.25 (7.69,36.33)	0.997(0.844,1.15)	<0.001
Turkmen	13.55	19.54	1.208(1.136,	0	342.57	499.41	1.242(1.161,	<0.0	3	4.36	1.232(1.127,	<0.0

istan	(9.31,19.36)	(13.43,28.02)	1.279)		(234.05,499.55)	(335.7,734.32)	1.322)	01	(1.25,6.37)	(1.84,9.24)	1.338)	01
Tuvalu	57.71 (39.75,81.88)	91.62 (63.54,130.16)	1.484(1.453,1.514)	0	1486.83 (1036.95,2108.99)	2359.26 (1626.1,3363.82)	1.468(1.39,1.547)	<0.001	13.01 (5.73,26.78)	20.62 (9.44,43.69)	1.471(1.389,1.552)	<0.001
Uganda	21.34 (14.86,30)	26.55 (18.65,37.74)	0.732(0.664,0.8)	0	540.08 (368.37,774.96)	672.52 (462.55,967.04)	0.733(0.666,0.799)	<0.001	4.68 (1.98,10.12)	5.78 (2.48,11.92)	0.704(0.624,0.784)	<0.001
Ukraine	8.31 (5.84,11.94)	9.84 (6.9,14.14)	0.56(0.521,0.6)	0	198.28 (133.72,288.75)	237.18 (161.22,349.27)	0.6(0.577,0.622)	<0.001	1.78 (0.74,3.5)	2.13 (0.89,4.54)	0.59(0.522,0.658)	<0.001
United Arab Emirates	70.28 (48.76,100.07)	92.6 (63.36,132.81)	0.909(0.876,0.943)	0	1846.08 (1267.87,2644.64)	2433.55 (1675.23,3522.8)	0.915(0.875,0.954)	<0.001	16.25 (7.2,33.99)	21.37 (9.46,44.43)	0.909(0.867,0.952)	<0.001
United Kingdom	119.73 (84.47,168.47)	150.78 (106.22,211.32)	0.743(0.683,0.802)	0	3066.85 (2131.89,4361.89)	3835.91 (2672.27,5436.12)	0.722(0.67,0.774)	<0.001	27.48 (12.3,56.71)	34.25 (15.52,70.62)	0.709(0.654,0.765)	<0.001
United Republic of Tanzania	24.52 (16.89,35.06)	29.83 (20.77,42.01)	0.647(0.617,0.677)	0	629.06 (426.38,919.78)	766.13 (524.33,1092.09)	0.644(0.627,0.661)	<0.001	5.35 (2.28,10.7)	6.54 (2.92,13.54)	0.651(0.613,0.689)	<0.001
United States of America	125.86 (88.65,176.34)	157.14 (116.75,206.42)	0.615(0.345,0.885)	0	3144.31 (2220.62,4423.21)	3935.93 (2940.53,5138.6)	0.652(0.406,0.898)	<0.001	28.11 (12.42,57.74)	34.83 (16.07,70.21)	0.638(0.477,0.8)	<0.001
United States	66.26 (45.08,96.	83.82 (56.13,120	0.758(0.72,0.796)	0	1724 (1162.13,253	2177.74 (1449.14,312	0.754(0.717,0.79)	<0.001	15.29 (6.65,32.6	19.22 (8.4,41.13	0.74(0.701,0.78)	<0.001

Virgin Islands	74)	.67)			7.13)	1.48)			9)	)		
Uruguay	47.08 (33.17,66.75)	77.63 (54.72,112.02)	1.632(1.58,1.684)	0	1191.16 (826.85,1708.67)	1968.43 (1386.43,2858.94)	1.651(1.601,1.701)	<0.001	10.56 (4.58,21.74)	17.38 (7.59,34.85)	1.63(1.567,1.693)	<0.001
Uzbekistan	13.93 (9.53,19.93)	19.28 (13.34,26.71)	1.06(1.005,1.114)	0	351.27 (234.79,514.12)	491.56 (333.67,691.99)	1.093(1.048,1.139)	<0.001	3.07 (1.26,6.56)	4.29 (1.84,9.33)	1.091(1.015,1.167)	<0.001
Vanuatu	52.13 (36.57,74.17)	77.93 (53.18,112.57)	1.282(1.256,1.309)	0	1341.47 (926.99,1923.6)	2007.65 (1362.31,2935.24)	1.262(1.199,1.326)	<0.001	11.75 (5.21,23.98)	17.58 (7.79,37.45)	1.26(1.194,1.326)	<0.001
Venezuela (Bolivarian Republic of)	77.95 (52.5,114.59)	98.33 (68.01,141.95)	0.744(0.711,0.776)	0	2011.15 (1356.69,2941.55)	2531.18 (1709.99,3685.17)	0.735(0.702,0.768)	<0.001	17.58 (7.64,37.43)	22.06 (9.61,46.27)	0.728(0.691,0.765)	<0.001
Viet Nam	45 (31.36,62.39)	90.74 (63.35,127.03)	2.321(2.252,2.39)	0	1133.23 (781.23,1595.74)	2281.42 (1589.77,3247.08)	2.319(2.247,2.392)	<0.001	9.95 (4.37,20.61)	20.02 (8.54,41.66)	2.315(2.242,2.388)	<0.001
Yemen	40.51 (28.47,58.27)	49.82 (33.8,71.41)	0.689(0.651,0.728)	0	1054.43 (736.22,1554.34)	1304.84 (876.74,1890.88)	0.706(0.678,0.733)	<0.001	9.25 (4.11,19.57)	11.45 (4.94,24.53)	0.702(0.644,0.761)	<0.001
Zambia	28.23 (19.39,40.23)	34.33 (23.39,48.84)	0.654(0.596,0.712)	0	725.52 (490.1,1053.21)	884.16 (592.83,1290.42)	0.66(0.598,0.723)	<0.001	6.19 (2.61,13.45)	7.6 (3.27,15.8)	0.687(0.629,0.744)	<0.001

Zimbabwe	29.99 (20.42,42.9)	32.29 (22.62,45.66)	0.28(0.159,0.402)	0	762.12 (511.8,1096.6)	823.01 (564.1,1177.99)	0.259(0.198,0.319)	<0.001	6.57 (2.87,13.85)	7.08 (3.01,15.03)	0.256(0.172,0.34)	<0.001

Table S9 The global burden of 10-24 PCOS in GBD regions, between 1990 and 2036.

	**Rate**			**Number**		
Year	incidence	prevalence	DALYs	incidence	prevalence	DALYs
1990	187.9652	1633.329057	14.84767	1430611	12431336.22	113006.2177
1991	189.2787	1651.146866	15.01912	1449498	12644498.39	115016.5433
1992	190.809	1666.970151	15.16534	1470281	12844862.92	116856.7297
1993	192.4156	1681.127928	15.29751	1493180	13045851.66	118711.431
1994	194.0541	1692.305077	15.4053	1518207	13239963.92	120525.3509
1995	195.6699	1699.878794	15.47516	1545336	13425076.55	122217.6253
1996	197.7187	1708.117137	15.55251	1577916	13631819.77	124118.5126
1997	200.4519	1721.593185	15.67234	1617265	13889973.15	126445.8726
1998	203.409	1738.352856	15.82161	1659650	14183529.94	129091.2883
1999	206.0591	1756.259497	15.98863	1700802	14496080.38	131969.3694
2000	207.9165	1773.211399	16.14566	1736255	14807617.46	134828.0881
2001	209.3467	1792.226588	16.32587	1767836	15134520.46	137864.369
2002	210.7779	1815.807426	16.54914	1798697	15495402.28	141223.9708
2003	211.9574	1841.184168	16.79531	1826200	15863419.46	144706.3646
2004	212.698	1866.033014	17.02888	1848232	16214830.66	147971.8824
2005	212.8416	1888.073364	17.23454	1863287	16528835.48	150877.0016
2006	212.3758	1906.198375	17.39701	1871956	16801913.55	153343.4585
2007	211.7081	1921.917111	17.54257	1877155	17041081.03	155544.8362
2008	211.1742	1937.37004	17.68516	1880888	17255783.24	157518.3466

2009	211.1349	1954.006365	17.82968	1885865	17453256.38	159255.355
2010	211.8464	1971.517457	17.98354	1893589	17622409.67	160745.873
2011	213.4525	1990.173156	18.14174	1906039	17771393.51	161997.9743
2012	215.6762	2009.536013	18.27887	1922961	17916955.91	162973.7507
2013	218.2526	2027.438825	18.40677	1942932	18048700.37	163861.0457
2014	220.8996	2042.041974	18.50729	1964345	18158812.04	164575.6179
2015	223.3706	2052.191208	18.5828	1986359	18249441.8	165250.527
2016	226.0543	2062.972613	18.6861	2014389	18383326.41	166513.4288
2017	229.3617	2078.198403	18.8338	2052511	18597368.93	168539.8146
2018	232.9046	2094.31907	18.98937	2096609	18853078.06	170942.4986
2019	236.2399	2108.763904	19.12308	2141787	19118375.93	173372.7953
2020	240.4725	2134.783083	19.34193	2196573	19499972.22	176677.0042
2021	242.5261	2141.618666	19.39231	2232036	19709917.6	178472.8417
2022	244.561	2153.066169	19.47991	2267415	19923847.02	180312.8665
2023	245.3764	2166.840921	19.58984	2287925	20140968.41	182189.7063
2024	246.1653	2181.79004	19.71318	2306538	20360647.21	184097.2189
2025	246.5228	2197.331764	19.84456	2316700	20582374.97	186030.2867
2026	247.093	2213.172526	19.98077	2327406	20805744.36	187984.6461
2027	247.7268	2229.164187	20.11987	2336010	21030429.02	189956.7447
2028	248.7122	2245.231995	20.26071	2348209	21256167.45	191943.6229
2029	249.9371	2261.338227	20.4026	2362519	21482750.17	193942.8148
2030	251.4722	2277.463849	20.54511	2381775	21710009.32	195952.2661
2031	253.1973	2293.599255	20.68799	2404392	21937810.43	197970.2649
2032	255.0825	2309.739598	20.8311	2431086	22166045.75	199995.3853
2033	257.023	2325.882433	20.97435	2460028	22394628.96	202026.439
2034	258.9726	2342.026525	21.11768	2490815	22623490.9	204062.4361

2035	260.8677	2358.171252	21.26106	2521786	22852576.15	206102.5519
2036	262.6873	2374.316298	21.40446	2552342	23081840.32	208146.0992

Table S10. Frontier DALYs,and effective difference by country or territory

Location	SDI	ASDR 2021	Frontier DALYs	Effective difference
Afghanistan	0.3372	11.105179	2.937900752	8.167277868
Albania	0.70685	1.6939818	1.087893838	0.606087974
Algeria	0.659501	19.418191	1.095451917	18.32273891
American Samoa	0.723728	24.405623	1.096071436	23.30955113
Andorra	0.869444	32.019246	1.085091806	30.93415392
Angola	0.453722	6.3422252	2.923622115	3.418603052
Antigua and Barbuda	0.749887	13.921026	1.081740621	12.83928539
Argentina	0.723123	15.89321	1.084759972	14.80844975
Armenia	0.701833	4.1486573	1.08939932	3.059257958
Australia	0.844253	40.196639	1.085844006	39.11079495
Austria	0.853837	34.937339	1.099291411	33.83804742
Azerbaijan	0.694851	4.4600245	1.080769287	3.379255249
Bahamas	0.805021	15.860376	1.094083184	14.76629291
Bahrain	0.753043	21.122009	1.084930981	20.03707794
Bangladesh	0.492421	5.4302955	2.3816782	3.048617333
Barbados	0.746749	15.411638	1.080792102	14.3308455
Belarus	0.784485	2.3494954	1.088919902	1.260575481
Belgium	0.853654	31.589488	1.088281533	30.50120664
Belize	0.610229	15.1203	1.095952037	14.02434845
Benin	0.373487	7.1983194	3.00439694	4.193922433
Bermuda	0.821365	17.712817	1.090125032	16.62269147

Bhutan	0.473062	8.4111773	2.402499981	6.008677364
Bolivia (Plurinational State of)	0.599011	26.007534	1.080862296	24.92667208
Bosnia and Herzegovina	0.723078	1.6687469	1.082887445	0.585859479
Botswana	0.642722	9.2256201	1.081892865	8.143727193
Brazil	0.653044	5.3786783	1.081893826	4.29678451
Brunei Darussalam	0.810234	33.119498	1.099631882	32.01986598
Bulgaria	0.768151	1.9002153	1.080377393	0.819837858
Burkina Faso	0.285118	5.976423	2.894923062	3.081499982
Burundi	0.289374	3.9849563	2.890713082	1.094243266
Cabo Verde	0.533535	7.2181381	2.384364577	4.833773544
Cambodia	0.473621	17.437979	2.408212167	15.02976657
Cameroon	0.479691	8.0828835	2.41738838	5.66549509
Canada	0.873171	16.476995	1.088044455	15.38895008
Central African Republic	0.309168	5.0171494	2.955364532	2.061784892
Chad	0.240436	4.4146827	2.895528273	1.519154411
Chile	0.771515	18.752416	1.088225541	17.66419
China	0.72163	13.340675	1.08480729	12.25586723
Colombia	0.655443	19.45477	1.088040777	18.36672901
Comoros	0.475979	6.8582945	2.381720114	4.476574418
Congo	0.583075	6.785749	1.090005733	5.695743316
Cook Islands	0.77911	27.02257	1.091236718	25.93133303
Costa Rica	0.70034	23.957924	1.083635596	22.87428824
Coed'Ivoire	0.425942	6.6311143	1.085030263	5.546084036
Croatia	0.798341	1.8520826	1.08983662	0.76224599
Cuba	0.66873	15.178489	1.080906846	14.09758176
Cyprus	0.835631	29.8057	1.0942226	28.71147695

Czechia	0.82845	1.8001728	1.089585634	0.710587128
Democratic People's Republic of Korea	0.569855	8.4779867	1.092652634	7.385334052
Democratic Republic of the Congo	0.38318	5.4906431	2.956144434	2.534498677
Denmark	0.896424	29.594995	1.082291905	28.51270326
Djibouti	0.487958	7.5760257	2.395196067	5.180829598
Dominica	0.746967	14.277171	1.089324619	13.18784617
Dominican Republic	0.619388	14.304983	1.089153317	13.21582976
Ecuador	0.661017	32.364798	1.096321116	31.26847651
Egypt	0.606787	20.619348	1.083094396	19.53625382
El Salvador	0.563775	20.895937	1.096156692	19.79978019
Equatorial Guinea	0.657857	9.4061504	1.081194086	8.324956298
Eritrea	0.403864	5.0361552	2.933155399	2.102999795
Estonia	0.844918	2.6808648	1.081067588	1.599797163
Eswatini	0.58546	9.775134	1.084599134	8.69053483
Ethiopia	0.358823	5.1761262	2.899673298	2.276452892
Fiji	0.675052	21.639939	1.087798917	20.5521405
Finland	0.859831	29.883612	1.089668199	28.79394421
France	0.838365	28.863167	1.084389104	27.77877805
Gabon	0.634691	8.3591932	1.086658652	7.272534546
Gambia	0.409714	6.0499702	2.904445092	3.145525088
Georgia	0.846564	6.3371961	1.097389859	5.239806246
Germany	0.902957	27.538715	1.082442975	26.4562723
Ghana	0.56493	6.264797	1.08510025	5.179696794
Greece	0.791854	32.306197	1.097420132	31.20877663
Greenland	0.82621	14.546448	1.099114525	13.44733385
Grenada	0.668993	12.850473	1.089201895	11.7612716

Guam	0.803982	27.468789	1.0946894	26.37409918
Guatemala	0.539972	17.698881	1.094159718	16.60472114
Guinea	0.336401	5.5277245	2.905813256	2.621911211
Guinea-Bissau	0.35311	5.5554814	2.898192795	2.657288591
Guyana	0.650812	13.32021	1.080577951	12.23963209
Haiti	0.448278	8.4174129	2.892273968	5.525138893
Honduras	0.513037	17.991197	2.381641391	15.60955579
Hungary	0.790755	1.8734838	1.087399662	0.786084116
Iceland	0.876362	33.432235	1.083645279	32.34858939
India	0.575402	11.073677	1.094236044	9.979440746
Indonesia	0.656868	25.384717	1.087763653	24.29695354
Iran (Islamic Republic of)	0.697207	20.32784	1.0830247	19.24481502
Iraq	0.662626	17.836685	1.084731354	16.75195377
Ireland	0.873754	30.91296	1.090689506	29.82227027
Israel	0.809012	28.122494	1.088407086	27.03408735
Italy	0.805774	71.693355	1.087065753	70.60628893
Jamaica	0.683263	13.602837	1.084788166	12.51804893
Japan	0.871242	55.143064	1.081438186	54.06162594
Jordan	0.725307	18.357363	1.080796118	17.27656699
Kazakhstan	0.725144	4.560451	1.082454601	3.477996359
Kenya	0.523768	6.4242029	2.384942158	4.039260723
Kiribati	0.527187	17.640321	2.382681998	15.25763871
Kuwait	0.846651	23.927405	1.083107099	22.84429833
Kyrgyzstan	0.603979	3.5530796	1.09533415	2.457745494
Lao People's Democratic Republic	0.489136	21.43408	2.382851863	19.05122836
Latvia	0.830664	2.4401885	1.089081272	1.351107189

Lebanon	0.744746	20.338249	1.085115557	19.25313321
Lesotho	0.510393	8.0808046	2.381737529	5.699067048
Liberia	0.352442	6.0296204	2.932045736	3.097574709
Libya	0.725771	19.605767	1.088524036	18.51724289
Lithuania	0.856484	2.3445656	1.082799893	1.261765686
Luxembourg	0.884429	32.386942	1.081143741	31.30579844
Madagascar	0.400247	4.8889205	2.911830918	1.977089572
Malawi	0.384554	6.1513422	2.899685919	3.251656296
Malaysia	0.742524	35.65852	1.081840188	34.5766801
Maldives	0.650887	29.199855	1.093960638	28.10589398
Mali	0.26858	4.994061	2.911374283	2.082686751
Malta	0.801585	32.209477	1.081140163	31.12833643
Marshall Islands	0.574091	17.237995	1.094800945	16.14319363
Mauritania	0.498945	7.3415449	2.408192826	4.933352066
Mauritius	0.71826	32.959442	1.085200954	31.87424109
Mexico	0.664575	31.263988	1.09094434	30.17304389
Micronesia (Federated States of)	0.587535	19.044726	1.094148354	17.95057774
Monaco	0.908263	32.582724	1.0852713	31.49745317
Mongolia	0.617622	4.0361588	1.084983384	2.951175445
Montenegro	0.795801	1.8875861	1.093196421	0.794389683
Morocco	0.562698	18.047846	1.083760087	16.96408596
Mozambique	0.326463	6.0695242	2.893105093	3.176419108
Myanmar	0.533901	21.968631	2.362164026	19.60646676
Namibia	0.617565	7.6935923	1.08411795	6.609474374
Nauru	0.625178	22.098997	1.081488425	21.01750901
Nepal	0.433175	5.8632197	2.890302944	2.972916764

Netherlands	0.888464	28.233001	1.084859483	27.14814141
New Zealand	0.849442	49.573073	1.097240949	48.47583216
Nicaragua	0.523958	19.534534	2.381665241	17.15286885
Niger	0.168073	4.465182	2.889793068	1.575388935
Nigeria	0.503391	6.3948824	2.381979756	4.012902676
Niue	0.726222	25.320538	1.085128034	24.23540986
North Macedonia	0.75063	1.7064653	1.085769599	0.620695734
Northern Mariana Islands	0.771535	24.115566	1.082291061	23.03327463
Norway	0.916133	28.804937	1.084734614	27.72020231
Oman	0.773392	21.196955	1.094798619	20.10215634
Pakistan	0.504029	6.8907082	2.383313449	4.507394747
Palau	0.754047	25.236549	1.095340832	24.14120864
Palestine	0.631012	17.104273	1.085351622	16.0189217
Panama	0.708865	20.117339	1.09556924	19.02177015
Papua New Guinea	0.417797	14.188292	2.901220083	11.28707207
Paraguay	0.635718	5.3043314	1.08081618	4.223515225
Peru	0.662054	27.921212	1.099200672	26.82201132
Philippines	0.651219	24.854655	1.088738584	23.7659166
Poland	0.812043	2.3627067	1.095219911	1.267486834
Portugal	0.744152	29.25462	1.084512125	28.17010831
Puerto Rico	0.825526	20.050866	1.084448689	18.9664174
Qatar	0.846861	23.426956	1.081305591	22.34565065
Republic of Korea	0.886675	27.227011	1.080809783	26.14620158
Republic of Moldova	0.732215	2.1999807	1.090242321	1.109738377
Romania	0.768454	1.8115696	1.082616267	0.728953329
Russian Federation	0.808536	2.4133327	1.083827137	1.32950557

Rwanda	0.435589	6.2256818	2.99245972	3.233222037
Saint Kitts and Nevis	0.754987	16.16843	1.088898044	15.07953235
Saint Lucia	0.67251	13.265041	1.094638885	12.17040186
Saint Vincent and the Grenadines	0.637196	13.614828	1.091666304	12.52316194
Samoa	0.593393	21.251092	1.096574776	20.15451753
San Marino	0.888005	31.488334	1.082738674	30.40559503
Sao Tome and Principe	0.505414	6.5290334	2.381716035	4.147317383
Saudi Arabia	0.815143	22.947172	1.081681075	21.86549081
Senegal	0.408054	6.0199818	2.987159938	3.032821885
Serbia	0.792416	1.7342838	1.081402163	0.652881685
Seychelles	0.730151	30.963675	1.091266285	29.87240902
Sierra Leone	0.358666	6.0835787	2.943542263	3.140036407
Singapore	0.856098	32.235629	1.08279553	31.15283359
Slovakia	0.810611	1.8323941	1.091296367	0.7410977
Slovenia	0.842431	1.922905	1.082123776	0.840781237
Solomon Islands	0.42936	15.985625	2.942217295	13.04340744
Somalia	0.077688	4.8162008	4.107641455	0.708559367
South Africa	0.679627	10.224953	1.095428175	9.129525135
South Sudan	0.278371	5.0862669	3.039286785	2.046980145
Spain	0.769284	29.302158	1.099470945	28.20268725
Sri Lanka	0.701535	27.800585	1.082915361	26.71767009
Sudan	0.54195	15.666739	1.094445895	14.57229328
Suriname	0.633666	14.041611	1.10443169	12.93717945
Sweden	0.88688	23.315872	1.08796159	22.2279103
Switzerland	0.933059	27.954443	1.096077793	26.85836492
Syrian Arab Republic	0.623004	17.702708	1.091596971	16.61111107

Taiwan (Province of China)	0.874747	21.509569	1.083874251	20.42569503
Tajikistan	0.541511	3.1025734	1.088768817	2.013804595
Thailand	0.682548	30.975014	1.088555402	29.88645814
Timor-Leste	0.444668	18.187912	2.937487898	15.25042393
Togo	0.408534	5.8114367	3.013323971	2.798112705
Tokelau	0.686426	22.85439	1.083622898	21.77076674
Tonga	0.62635	24.378768	1.0874148	23.29135337
Trinidad and Tobago	0.768763	15.07673	1.082577278	13.99415266
Tunisia	0.682432	18.483697	1.094223066	17.38947347
Turkey	0.712693	17.247504	1.0890528	16.15845092
Turkmenistan	0.682161	4.355616	1.08798277	3.267633232
Tuvalu	0.576621	20.621591	1.088374524	19.53321612
Uganda	0.423261	5.7750051	2.89793662	2.87706852
Ukraine	0.760774	2.1270556	1.081191552	1.045864029
United Arab Emirates	0.849318	21.374499	1.085596615	20.28890246
United Kingdom	0.859	34.249192	1.081232087	33.16796031
United Republic of Tanzania	0.446568	6.537738	2.939088981	3.598649051
United States of America	0.862448	34.826624	1.094007229	33.73261691
United States Virgin Islands	0.821831	19.220246	1.096292493	18.12395359
Uruguay	0.719283	17.381056	1.082790788	16.29826548
Uzbekistan	0.662622	4.2905129	1.094475154	3.196037782
Vanuatu	0.473101	17.580738	2.455408852	15.12532891
Venezuela (Bolivarian Republic of)	0.596513	22.058493	1.093040561	20.9654528
Viet Nam	0.627934	20.023908	1.089289691	18.93461843
Yemen	0.450376	11.449259	2.905057173	8.544201657
Zambia	0.505949	7.5953068	2.391366358	5.203940424

Zimbabwe	0.473819	7.0763509	2.396162144	4.680188706

Table S11. Frontier DALYs and Analysis of Effective Differences in the 15 Lowest-Ranked Countries or Territories Globally

Location	SDI	ASDR 2021	Frontier DALYs	Effective difference
Italy	0.805774	71.69335	1.087066	70.60629
Japan	0.871242	55.14306	1.081438	54.06163
New Zealand	0.849442	49.57307	1.097241	48.47583
Australia	0.844253	40.19664	1.085844	39.11079
Malaysia	0.742524	35.65852	1.08184	34.57668
Austria	0.853837	34.93734	1.099291	33.83805
United States of America	0.862448	34.82662	1.094007	33.73262
United Kingdom	0.859	34.24919	1.081232	33.16796
Iceland	0.876362	33.43223	1.083645	32.34859
Brunei Darussalam	0.810234	33.1195	1.099632	32.01987
Mauritius	0.71826	32.95944	1.085201	31.87424
Monaco	0.908263	32.58272	1.085271	31.49745
Luxembourg	0.884429	32.38694	1.081144	31.3058
Ecuador	0.661017	32.3648	1.096321	31.26848
Greece	0.791854	32.3062	1.09742	31.20878

Table S12. Frontier DALYs and Analysis of Effective Differences in the Top 5 Performing Countries or Territories in Low-SDI Regions

Location	SDI	ASDR 2021	Frontier DALYs	Effective difference
Somalia	0.077688	4.816201	4.107641	0.708559
Burundi	0.289374	3.984956	2.890713	1.094243
Chad	0.240436	4.414683	2.895528	1.519154
Niger	0.168073	4.465182	2.889793	1.575389
Madagascar	0.400247	4.88892	2.911831	1.97709

Table S13. Frontier DALYs and Analysis of Effective Differences in the 5 Lowest-Performing Countries or Territories in High-SDI Regions

Location	SDI	ASDR 2021	Frontier DALYs	Effective difference
Japan	0.871242	55.14306	1.081438	54.06163
Austria	0.853837	34.93734	1.099291	33.83805
United States of America	0.862448	34.82662	1.094007	33.73262
United Kingdom	0.859	34.24919	1.081232	33.16796
Iceland	0.876362	33.43223	1.083645	32.34859

Table S14. Incidence of PCOS in adolescents and young adults aged 10-24 years in 2021 and their AAPCs between 1990-2021 in 204 countries and territories

	**Cases (n), 1990**	**Incidence (per100,000 population), 1990**	**Cases (n), 2021**	**Incidence (per100,000 population), 2021**	**AAPC (95%CI)**	***P* value**
**Afghanistan**	**2,450 (1,683-3,481)**	**141.61**	**10,325 (7,018-14,910)**	**199.77**	**1.102(0.818-**	**<0.001**

		(97.26-201.21)		(135.78-288.48)	1.387)	
Albania	101 (64-157)	20.97 (13.32-32.45)	65 (42-97)	25.84 (16.83-38.89)	0.697(0.628-0.766)	<0.001
Algeria	9,615 (6,573-13,718)	229.7 (157.02-327.71)	16,675 (11,097-24,172)	335.32 (223.15-486.09)	1.255(1.196-1.313)	<0.001
American Samoa	22 (15-30)	294.96 (205.85-415.56)	31 (21-46)	460.54 (316.64-676.35)	1.44(1.348-1.532)	<0.001
Andorra	24 (16-34)	407.25 (277.83-588.3)	33 (23-47)	526.27 (362.04-744.68)	0.882(0.604-1.161)	<0.001
Angola	966 (650-1,418)	60.64 (40.79-89.05)	6,130 (4,074-9,009)	113.65 (75.54-167.05)	2.072(1.969-2.175)	<0.001
Antigua and Barbuda	15 (10-21)	170.01 (113.23-241.15)	21 (14-30)	217.92 (148.29-309.53)	0.786(0.709-0.864)	<0.001
Argentina	7,990 (5,471-11,533)	183.19 (125.44-264.42)	14,205 (9,979-20,450)	268.55 (188.66-386.63)	1.257(1.223-1.291)	<0.001
Armenia	197 (132-289)	46.04 (30.9-67.46)	183 (121-272)	72.06 (47.76-106.89)	1.463(1.379-1.546)	<0.001
Australia	12,069 (8,930-15,625)	619.55 (458.4-802.07)	16,983 (12,067-23,749)	738.34 (524.64-1,032.52)	0.575 (0.482-0.667)	<0.001

**Austria**	**3,802 (2,721-5,214)**	**479.25 (342.92-657.21)**	**3,718 (2,588-5,196)**	**548.41 (381.78-766.43)**	**0.442 (0.284-0.599)**	**<0.001**
**Azerbaijan**	**479 (322-702)**	**45.84 (30.82-67.21)**	**755 (499-1,076)**	**72.57 (47.98-103.48)**	**1.516 (1.447-1.585)**	**<0.001**
**Bahamas**	**88 (58-128)**	**216.96 (143.6-316.49)**	**133 (90-195)**	**266.83 (179.42-391.01)**	**0.675 (0.613-0.738)**	**<0.001**
**Bahrain**	**187 (127-268)**	**301.13 (204.74-430.65)**	**490 (340-694)**	**342.1 (237.26-483.86)**	**0.427 (0.124-0.731)**	**0.006**
**Bangladesh**	**9,745 (6,443-14,182)**	**55.06 (36.4-80.12)**	**21,097 (14,596-29,694)**	**89.47 (61.9-125.93)**	**1.588 (1.455-1.721)**	**<0.001**
**Barbados**	**70 (47-101)**	**210.31 (140.85-302.11)**	**69 (47-98)**	**245.64 (167.22-348.74)**	**0.505 (0.383-0.628)**	**<0.001**
**Belarus**	**309 (205-461)**	**28.09 (18.63-41.91)**	**264 (174-396)**	**38.3 (25.27-57.37)**	**1.035 (0.985-1.085)**	**<0.001**
**Belgium**	**4,117 (2,795-5,885)**	**418.12 (283.8-597.61)**	**5,010 (3,518-7,060)**	**520.73 (365.66-733.83)**	**0.713 (0.654-0.771)**	**<0.001**
**Belize**	**58 (38-83)**	**185.48 (122.12-267.56)**	**166 (114-239)**	**250.65 (171.43-360.19)**	**0.992 (0.922-1.062)**	**<0.001**
**Benin**	**503 (343-729)**	**68.05 (46.42-98.49)**	**2,872 (1,911-4,179)**	**128.85 (85.74-187.48)**	**2.088 (1.98-2.196)**	**<0.001**

**Bermuda**	**15 (10-21)**	**239.77 (157.77-344.97)**	**13 (9-20)**	**290.77 (197.63-430.51)**	**0.642 (0.552-0.733)**	**<0.001**
**Bhutan**	**84 (57-119)**	**80.63 (54.44-114.32)**	**136 (93-197)**	**136.21 (92.88-197.25)**	**1.724 (1.616-1.833)**	**<0.001**
**Bolivia (Plurinational State of)**	**3,794 (2,530-5,563)**	**376.63 (251.11-552.21)**	**7,400 (4,958-10,444)**	**462.78 (310.04-653.18)**	**0.678 (0.635-0.722)**	**<0.001**
**Bosnia and Herzegovina**	**96 (59-154)**	**17.35 (10.64-27.72)**	**70 (46-104)**	**26.4 (17.28-39.61)**	**1.377 (1.215-1.539)**	**<0.001**
**Botswana**	**253 (172-370)**	**107.94 (73.08-157.69)**	**524 (349-746)**	**161.1 (107.23-229.27)**	**1.309 (1.099-1.519)**	**<0.001**
**Brazil**	**20,595 (13,684-30,331)**	**87.98 (58.46-129.58)**	**20,903 (14,226-29,639)**	**87.24 (59.37-123.7)**	**-0.01 (-0.182-0.162)**	**0.911**
**Brunei Darussalam**	**142 (98-206)**	**386.85 (266.94-560.18)**	**296 (202-419)**	**599.65 (410.62-849.86)**	**1.429 (1.357-1.502)**	**<0.001**
**Bulgaria**	**224 (142-341)**	**24.85 (15.79-37.85)**	**152 (99-228)**	**32.27 (21-48.37)**	**0.859 (0.696-1.021)**	**<0.001**
**Burkina Faso**	**992 (672-1,461)**	**66.32 (44.91-97.67)**	**4,004 (2691-5682)**	**105.85 (71.14-150.2)**	**1.538 (1.452-1.624)**	**<0.001**
**Burundi**	**559 (376-817)**	**65.24 (43.84-95.33)**	**1,579 (1,063-2,281)**	**71.32 (48.03-103.03)**	**0.323 (0.187-0.459)**	**<0.001**
**Cabo Verde**	**43 (29-62)**	**74.26 (50.01-107.78)**	**89 (61-129)**	**120.75 (82.91-173.86)**	**1.584 (1.456-1.713)**	**<0.001**

**Cambodia**	**2,963 (2,009-4,252)**	**179.74 (121.92-257.94)**	**6,868 (4,793-9,853)**	**303.45 (211.77-435.32)**	**1.735 (1.692-1.778)**	**<0.001**
**Cameroon**	**1,706 (1,124-2,468)**	**103.46 (68.2-149.7)**	**7,305 (4,777-10,652)**	**140.71 (92.01-205.17)**	**1.007 (0.919-1.094)**	**<0.001**
**Canada**	**6,142 (4,249-8,922)**	**214.75 (148.56-311.95)**	**8,717 (6,043-12,596)**	**278.27 (192.92-402.08)**	**0.834 (0.8-0.867)**	**<0.001**
**Central African Republic**	**331 (227-489)**	**77.79 (53.47-114.9)**	**787 (531-1,128)**	**85.51 (57.66-122.6)**	**0.328 (0.281-0.376)**	**<0.001**
**Chad**	**479 (314-697)**	**50.98 (33.43-74.19)**	**2,365 (1,599-3,424)**	**79.95 (54.04-115.74)**	**1.477 (1.336-1.618)**	**<0.001**
**Chile**	**3,485 (2,426-5,031)**	**185.98 (129.46-268.47)**	**5,950 (4169-8614)**	**311.41 (218.17-450.81)**	**1.63 (1.476-1.783)**	**<0.001**
**China**	**194,627 (133,727-274,770)**	**110.83 (76.15-156.46)**	**244,889 (169,307-349547)**	**224.47 (155.19-320.4)**	**2.364 (2.186-2.542)**	**<0.001**
**Colombia**	**13,197 (8,847-19,442)**	**253.81 (170.15-373.92)**	**18,030 (12,351-26,512)**	**308.96 (211.65-454.31)**	**0.637 (0.592-0.681)**	**<0.001**
**Comoros**	**77 (52-113)**	**99.49 (67.18-146.71)**	**124 (83-177)**	**113.28 (76.29-162.02)**	**0.444 (0.331-0.557)**	**<0.001**
**Congo**	**355 (238-515)**	**86.18 (57.8-125.03)**	**1,015 (689-1,470)**	**120.68 (81.95-174.75)**	**1.107 (1.03-1.183)**	**<0.001**
**Cook Islands**	**9 (6-13)**	**320.56 (215.42-467.04)**	**10 (7-14)**	**458.75 (316.79-667.27)**	**1.165 (1.017-1.314)**	**<0.001**
**Costa Rica**	**1,436 (964-2,138)**	**313.57**	**2,124**	**388.51**	**0.694**	**<0.001**

		(210.5-466.87)	(1,430-3,050)	(261.7-558)	(0.659-0.729)	
**Coted'Ivoire**	**1,319 (880-1,949)**	**68.73 (45.84-101.56)**	**4,835 (3,224-7,194)**	**29.37 (19.02-43.5)**	**1.715 (1.628-1.802)**	**<0.001**
**Croatia**	**112 (73-174)**	**22.23 (14.49-34.36)**	**95 (61-140)**	**237.28 (159.12-334.07)**	**0.898 (0.81-0.985)**	**<0.001**
**Cuba**	**2,611 (1,756-3,704)**	**171.73 (115.49-243.63)**	**2,238 (1,501-3,152)**	**472.12 (324.17-678.57)**	**1.045 (0.927-1.162)**	**<0.001**
**Cyprus**	**311 (214-438)**	**340.48 (234.65-479.05)**	**493 (338-708)**	**31.02 (19.9-47.05)**	**1.041 (0.902-1.18)**	**<0.001**
**Czechia**	**283 (176-429)**	**24.33 (15.15-36.92)**	**236 (151-358)**	**116.17 (77.46-172.85)**	**0.763 (0.625-0.901)**	**<0.001**
**Democratic People's Republic of Korea**	**3,662 (2,567-5,277)**	**123.28 (86.42-177.65)**	**3,715 (2,532-5,251)**	**139.18 (94.87-196.72)**	**0.397 (0.369-0.426)**	**<0.001**
**Democratic Republic of the Congo**	**3,879 (2,616-5,707)**	**65 (43.83-95.64)**	**14,254 (9,713-20,691)**	**97.17 (66.21-141.05)**	**1.372 (1.229-1.516)**	**<0.001**
**Denmark**	**2,008 (1,382-2,869)**	**376.64 (259.25-538.02)**	**2,491 (1,719-3,565)**	**486.67 (335.71-696.39)**	**0.827 (0.753-0.901)**	**<0.001**
**Djibouti**	**53 (36-78)**	**76 (51.67-111.79)**	**203 (135-299)**	**127.94 (85.31-188.74)**	**1.723(1.598-1.849)**	**<0.001**
**Dominica**	**19 (13-29)**	**176.87 (118.63-259.8)**	**19 (13-27)**	**239.07 (158.75-339.58)**	**0.986(0.91-1.062)**	**<0.001**

**Dominican Republic**	**1,881 (1,274-2,718)**	**153.11 (103.69-221.27)**	**3,301 (2,193-4,702)**	**233.03 (154.79-331.94)**	**1.374(1.315-1.434)**	**<0.001**
**Ecuador**	**7,594 (5,227-10,738)**	**461.95 (317.97-653.18)**	**13,887 (9,683-19,218)**	**578.95 (403.72-801.24)**	**0.716(0.633-0.8)**	**<0.001**
**Egypt**	**23,053 (15,601-33,313)**	**277.93 (188.09-401.63)**	**49,864 (34,360-71,102)**	**347.98 (239.78-496.19)**	**0.718(0.613-0.823)**	**<0.001**
**El Salvador**	**2,418 (1,611-3,609)**	**267.52 (178.27-399.35)**	**3020 (2,014-4,401)**	**346.64 (231.18-505.22)**	**0.83(0.748-0.913)**	**<0.001**
**Equatorial Guinea**	**46 (31-69)**	**69.19 (46.61-102.15)**	**375 (251-537)**	**163.78 (109.92-234.64)**	**2.835(2.651-3.019)**	**<0.001**
**Eritrea**	**315 (211-452)**	**57.43 (38.44-82.57)**	**856 (581-1250)**	**85.27 (57.92-124.48)**	**1.32(1.216-1.424)**	**<0.001**
**Estonia**	**48 (32-71)**	**30.29 (20.23-44.97)**	**43 (29-66)**	**44.09 (29.01-67.02)**	**1.249(1.192-1.305)**	**<0.001**
**Eswatini**	**205 (139-296)**	**142.34 (96.31-205.77)**	**306 (209-436)**	**169.15 (115.48-240.91)**	**0.589(0.469-0.71)**	**<0.001**
**Ethiopia**	**4,505 (2,980-6,607)**	**56.2 (37.17-82.41)**	**16,137 (11,066-23,324)**	**86.92 (59.6-125.64)**	**1.428(1.397-1.459)**	**<0.001**
**Fiji**	**285 (194-412)**	**244.02 (166.27-352.53)**	**441 (305-638)**	**376.24 (260.06-544.26)**	**1.403(1.345-1.46)**	**<0.001**
**Finland**	**1,856 (1,251-2,683)**	**389.58 (262.63-563.33)**	**2,262 (1,548-3,236)**	**506.13 (346.38-724)**	**0.851(0.754-0.949)**	**<0.001**
**France**	**22,333 (15,412-31,833)**	**360.14 (248.53-513.32)**	**28,471 (19,558-40,536)**	**476.27 (327.18-678.09)**	**0.885(0.811-0.959)**	**<0.001**
**Gabon**	**151 (102-219)**	**95.77 (64.72-139.15)**	**429 (285-632)**	**143.82 (95.62-211.87)**	**1.308(1.221-1.395)**	**<0.001**

**Gambia**	**112 (75-160)**	**69.67 (46.78-100.05)**	**439 (301-637)**	**105.72 (72.51-153.39)**	**1.371(1.249-1.494)**	**<0.001**
**Georgia**	**373 (243-543)**	**58.84 (38.37-85.75)**	**316 (214-444)**	**107.89 (72.96-151.73)**	**2.026(1.889-2.163)**	**<0.001**
**Germany**	**23,614 (16,461-32,932)**	**325.67 (227.03-454.19)**	**26,655 (18,585-37,589)**	**445.99 (310.96-628.94)**	**0.997(0.84-1.154)**	**<0.001**
**Ghana**	**1,712 (1,160-2,467)**	**72.54 (49.14-104.52)**	**5,636 (3,909-8,224)**	**107.34 (74.46-156.64)**	**1.268(1.212-1.325)**	**<0.001**
**Greece**	**4,869 (3,308-6,903)**	**424.07 (288.15-601.26)**	**4,140 (2,785-5,925)**	**546.43 (367.53-781.99)**	**0.816(0.765-0.866)**	**<0.001**
**Greenland**	**10 (7-14)**	**156.6 (110.1-220.13)**	**14 (10-20)**	**248.27 (175.27-356.95)**	**1.501(1.289-1.714)**	**<0.001**
**Grenada**	**20 (14-29)**	**156.9 (105.09-226.57)**	**24 (16-34)**	**200.05 (134.32-282.51)**	**0.8(0.689-0.91)**	**<0.001**
**Guam**	**57 (39-83)**	**321.51 (218.47-462.53)**	**81 (56-115)**	**470.9 (324.08-665.95)**	**1.233(1.173-1.293)**	**<0.001**
**Guatemala**	**3,325 (2,203-4,842)**	**242.3 (160.56-352.82)**	**7,481 (5,004-10,918)**	**300.23 (200.83-438.13)**	**0.68(0.624-0.737)**	**<0.001**
**Guinea**	**541 (358-778)**	**63.13 (41.81-90.88)**	**2,183 (1,492-3,183)**	**98.3 (67.17-143.32)**	**1.441(1.333-1.55)**	**<0.001**
**Guinea-Bissau**	**107 (72-159)**	**64.43 (43.29-96.21)**	**330 (225-480)**	**97.59 (66.65-142.1)**	**1.356(1.245-1.467)**	**<0.001**
**Guyana**	**204 (136-298)**	**155.13 (103.5-226.86)**	**219 (146-310)**	**211.14 (141.27-299.6)**	**1.006(0.888-1.124)**	**<0.001**
**Haiti**	**1,220 (796-1,802)**	**121.35 (79.23-179.31)**	**2,752 (1,858-3,988)**	**146.99 (99.23-213.01)**	**0.639(0.525-0.753)**	**<0.001**

**Honduras**	**1,755 (1,166-2,586)**	**226.89 (150.79-334.42)**	**4,899 (3,319-7,056)**	**306.85 (207.89-442.02)**	**0.975(0.943-1.007)**	**<0.001**
**Hungary**	**295 (186-443)**	**26.19 (16.56-39.44)**	**216 (142-330)**	**30.61 (20.13-46.71)**	**0.483(0.282-0.684)**	**<0.001**
**Iceland**	**136 (95-194)**	**439.58 (306.31-626.16)**	**186 (127-261)**	**558.23 (381.89-783.31)**	**0.764(0.657-0.871)**	**<0.001**
**India**	**123,779 (86,749-176,204)**	**100.07 (70.13-142.45)**	**329,841 (230,378-462,508)**	**172.71 (120.63-242.18)**	**1.806(1.72-1.892)**	**<0.001**
**Indonesia**	**66,946 (46,858-94,063)**	**224.29 (156.99-315.14)**	**140,300 (98,986-197,447)**	**418.3 (295.12-588.68)**	**2.046(1.999-2.093)**	**<0.001**
**Iran (Islamic Republic of)**	**23,321 (15,995-33,373)**	**257.24 (176.43-368.11)**	**28,786 (19,863-41,239)**	**333.01 (229.78-477.07)**	**0.888(0.73-1.047)**	**<0.001**
**Iraq**	**8,117 (5,522-11,812)**	**275.52 (187.44-400.96)**	**18,546 (12,614-26,545)**	**306.29 (208.32-438.39)**	**0.368(0.257-0.479)**	**<0.001**
**Ireland**	**2,126 (1,425-3,016)**	**447.26 (299.93-634.67)**	**2,613 (1,828-3,764)**	**541.88 (378.97-780.39)**	**0.617(0.536-0.698)**	**<0.001**
**Israel**	**2,600 (1,792-3,746)**	**391.24 (269.69-563.57)**	**5,468 (3,755-7,725)**	**496.95 (341.28-702.04)**	**0.769(0.751-0.788)**	**<0.001**
**Italy**	**76,492 (54,195-107,226)**	**1246.82 (883.38-1747.8)**	**51,372 (35,957-72,126)**	**1225.13 (857.51-1720.08)**	**-0.049(-0.143-0.044)**	**0.301**
**Jamaica**	**652 (437-917)**	**169.3 (113.35-238.01)**	**733 (490-1,042)**	**210.14 (140.58-298.91)**	**0.703(0.64-0.765)**	**<0.001**
**Japan**	**135,250 (94,903-192,844)**	**985.75 (691.69-1405.51)**	**86,680 (61,099-122,862)**	**1012.94 (714-1435.76)**	**0.111(0.038-0.184)**	**0.003**
**Jordan**	**1,618 (1,113-2,305)**	**253.18**	**5,672 (3,898-8,086)**	**315.34 (216.71-**	**0.754(0.618-**	**<0.001**

		(174.24-360.75)		449.54)	0.89)	
Kazakhstan	1,175 (779-1,741)	54.7 (36.26-81.05)	1,611 (1,062-2,336)	79.83 (52.64-115.75)	1.231(1.149-1.313)	<0.001
Kenya	3,623 (2,467-5,295)	89.92 (61.22-131.39)	9,372 (6,437-13,510)	108.57 (74.58-156.51)	0.639(0.561-0.717)	<0.001
Kiribati	22 (15-31)	198.67 (135.18-279.51)	55 (38-78)	311.76 (215.84-442.79)	1.46(1.33-1.591)	<0.001
Kuwait	721 (496-1,038)	319.96 (219.75-460.51)	1545 (1,068-2,229)	380.99 (263.31-549.4)	0.584(0.34-0.828)	<0.001
Kyrgyzstan	323 (213-472)	50.46 (33.29-73.61)	523 (340-770)	60.01 (39-88.31)	0.581(0.496-0.666)	<0.001
Lao People's Democratic Republic	1,366 (947-1,945)	206.5 (143.17-294.03)	3,765 (2,640-5,324)	364.35 (255.51-515.23)	1.874(1.815-1.933)	<0.001
Latvia	78 (51-119)	29.54 (19.5-45.24)	54 (36-80)	40.68 (27.12-60.45)	1.05(0.995-1.105)	<0.001
Lebanon	1,110 (765-1,592)	261.49 (180.27-375.08)	1949 (1341-2792)	349.09 (240.16-500.04)	0.996(0.805-1.188)	<0.001
Lesotho	247 (170-362)	97.4 (66.9-143)	424 (286-616)	138.69 (93.7-201.36)	1.176(1.104-1.248)	<0.001
Liberia	269 (181-398)	72.48 (48.86-107.38)	970 (661-1,415)	108.18 (73.77-157.83)	1.283(1.017-1.55)	<0.001
Libya	2,341 (1,583-3,369)	321.36 (217.29-462.56)	2,723 (1,864-3,901)	318.52 (218.06-456.37)	-0.006(-0.049-0.036)	0.767
Lithuania	107 (70-161)	26.77 (17.47-40.05)	73 (49-109)	36.4 (24.4-54.44)	1.03(0.906-1.154)	<0.001

**Luxembourg**	**133 (90-191)**	**375.3 (254.25-537.51)**	**267 (184-380)**	**517.6 (355.55-736.29)**	**1.051(0.97-1.132)**	**<0.001**
**Madagascar**	**1,404 (953-2,077)**	**72.69 (49.34-107.52)**	**4,074 (2,726-5,897)**	**83.55 (55.9-120.94)**	**0.491(0.402-0.581)**	**<0.001**
**Malawi**	**1,549 (1,042-2,268)**	**96.18 (64.72-140.81)**	**4,120 (2,812-5,958)**	**113.48 (77.46-164.13)**	**0.55(0.496-0.605)**	**<0.001**
**Malaysia**	**9,841 (6,872-14,039)**	**369.32 (257.9-526.85)**	**23,577 (16,458-32,960)**	**600.24 (419.02-839.13)**	**1.593(1.48-1.706)**	**<0.001**
**Maldives**	**77 (53-109)**	**215.82 (148.62-304.31)**	**223 (155-320)**	**498.19 (344.99-713.13)**	**2.768(2.685-2.851)**	**<0.001**
**Mali**	**699 (468-1,030)**	**53.86 (36.04-79.36)**	**3,568 (2,374-5,140)**	**88.99 (59.22-128.21)**	**1.648(1.526-1.77)**	**<0.001**
**Malta**	**166 (116-236)**	**417.6 (291.43-594.34)**	**159 (108-226)**	**513.96 (348.83-733.49)**	**0.648(0.55-0.746)**	**<0.001**
**Marshall Islands**	**14 (9-21)**	**188.78 (125.86-276.05)**	**25 (17-35)**	**303.47 (209.33-424.98)**	**1.526(1.484-1.567)**	**<0.001**
**Mauritania**	**292 (200-435)**	**90.71 (62.13-135)**	**945 (641-1,369)**	**129.02 (87.45-186.85)**	**1.159(1.06-1.258)**	**<0.001**
**Mauritius**	**560 (394-797)**	**349.95 (246.15-498.65)**	**691 (486-969)**	**539.3 (379.86-757.05)**	**1.396(1.247-1.546)**	**<0.001**
**Mexico**	**83,994 (57,279-119,124)**	**570.39 (388.97-808.95)**	**88,467 (61,806-123,445)**	**536.14 (374.57-748.12)**	**-0.2(-0.397--0.003)**	**0.047**
**Micronesia (Federated States of)**	**42 (29-60)**	**245.93 (168.94-352.21)**	**51 (35-71)**	**332.16 (231.28-469.94)**	**0.913(0.838-0.988)**	**<0.001**
**Monaco**	**8 (6-12)**	**387.24**	**14 (9-20)**	**535.58 (364.66-**	**1.052(0.902-**	**<0.001**

		(267.95-554.4)		762.95)	1.201)	
Mongolia	170 (110-245)	48.2 (31.23-69.77)	258 (171-376)	69.05 (45.73-100.55)	1.172(1.135-1.208)	<0.001
Montenegro	19 (12-29)	24.43 (15.74-37.41)	17 (11-26)	30.5 (19.93-45.73)	0.751(0.694-0.809)	<0.001
Morocco	9,458 (6,358-13,455)	232.75 (156.46-331.1)	13,648 (9,454-19,763)	297.66 (206.2-431.04)	0.828(0.787-0.87)	<0.001
Mozambique	1,566 (1,042-2,294)	71.82 (47.8-105.25)	5,936 (3,966-8,607)	107.8 (72.02-156.29)	1.312(1.224-1.399)	<0.001
Myanmar	12,132 (8,427-17,421)	183.72 (127.62-263.82)	28,792 (20,372-40,779)	377.38 (267.02-534.49)	2.383(2.274-2.491)	<0.001
Namibia	245 (169-350)	101.72 (70.17-145.11)	503 (345-727)	133.7 (91.7-193.33)	0.927(0.852-1.002)	<0.001
Nauru	4 (3-6)	270.51 (184.92-387.66)	6 (5-9)	390.96 (271.16-561.65)	1.163(1.085-1.241)	<0.001
Nepal	1,709 (1,164-2,472)	56.86 (38.73-82.23)	4,594 (3,127-6,501)	95.64 (65.1-135.34)	1.702(1.618-1.785)	<0.001
Netherlands	5,411 (3,715-7,698)	337.67 (231.84-480.33)	6,673 (4,631-9,601)	453.55 (314.76-652.55)	0.952(0.921-0.984)	<0.001
New Zealand	3,758 (2,633-5,303)	906.8 (635.39-1279.53)	4,342 (3,073-6,010)	889.53 (629.43-1231.24)	-0.053(-0.185-0.08)	0.437
Nicaragua	1,716 (1,137-2,545)	256.57 (169.92-380.39)	3,098 (2,059-4,507)	336.29 (223.51-489.26)	0.876(0.802-0.95)	<0.001
Niger	730 (487-1,042)	57.3 (38.27-81.8)	3,420 (2,354-4,978)	80.05 (55.09-116.54)	1.09(0.979-1.201)	<0.001
Nigeria	11,917 (8,102-17,313)	79.49	44,352 (30,194-	108.57 (73.91-	1.026(0.904-	<0.001

		(54.05-115.49)	64,127)	156.97)	1.148)	
Niue	1 (1-1)	303.9 (200.29-437.38)	1 (1-1)	442.25 (307.59-636.54)	1.193(1.051-1.335)	<0.001
North Macedonia	51 (32-80)	20.29 (13.01-32.05)	48 (31-72)	26.72 (17.38-40.39)	0.907(0.846-0.968)	<0.001
Northern Mariana Islands	19 (13-27)	283.66 (192.05-400.48)	24 (16-34)	429.45 (295-610.54)	1.497(0.744-2.256)	<0.001
Norway	1,741 (1,221-2,454)	389.02 (272.95-548.45)	2,247 (1,582-3,194)	469.34 (330.3-667.12)	0.649(0.484-0.815)	<0.001
Oman	579 (378-844)	237.42 (155.01-346.26)	1,611 (1,091-2,323)	371.68 (251.62-535.89)	1.473(1.396-1.55)	<0.001
Pakistan	16,824 (11,643-24,311)	99.4 (68.79-143.63)	39,368 (26,913-57,047)	112.57 (76.95-163.12)	0.408(0.345-0.471)	<0.001
Palau	7 (5-10)	299.65 (204.48-433.78)	7 (5-11)	454.42 (308.89-655.92)	1.311(1.164-1.458)	<0.001
Palestine	783 (528-1,133)	236.35 (159.18-341.84)	2,365 (1,611-3,333)	293.5 (199.92-413.56)	0.698(0.643-0.754)	<0.001
Panama	814 (549-1,177)	217.72 (146.74-314.83)	1,842 (1,248-2,704)	342.56 (232.01-502.87)	1.467(1.42-1.515)	<0.001
Papua New Guinea	1,076 (731-1,524)	171.1 (116.14-242.22)	3,620 (2,485-5,098)	249.99 (171.59-352.01)	1.213(1.155-1.27)	<0.001
Paraguay	350 (226-534)	56.98 (36.71-86.75)	838 (558-1,234)	86.02 (57.25-126.64)	1.343(1.307-1.378)	<0.001
Peru	13,636 (9,320-19,558)	385.43 (263.43-552.83)	20,883 (14,048-30,317)	478.85 (322.12-695.18)	0.721(0.656-0.786)	<0.001
Philippines	25,601 (17,797-36,125)	251.38	66,527 (46,839-	419.96 (295.67-	1.69(1.594-	<0.001

		(174.75-354.73)	93,074)	587.53)	1.786)	
**Poland**	**1,591 (1,023-2,419)**	**37.95 (24.39-57.69)**	**1,095 (770-1,494)**	**38.58 (27.13-52.64)**	**0.01(-0.083-0.103)**	**0.833**
**Portugal**	**4,470 (3,098-6,390)**	**363.65 (252.05-519.8)**	**3,773 (2,567-5,353)**	**469.5 (319.43-666.09)**	**0.838(0.76-0.916)**	**<0.001**
**Puerto Rico**	**1,305 (867-1,865)**	**267.2 (177.59-381.91)**	**954 (641-1381)**	**316.06 (212.33-457.75)**	**0.53(0.452-0.609)**	**<0.001**
**Qatar**	**140 (95-202)**	**356.68 (242.34-514)**	**702 (469-1,031)**	**414.07 (276.93-608.23)**	**0.469(0.314-0.624)**	**<0.001**
**Republic of Korea**	**20,969 (14,361-29,575)**	**329.4 (225.6-464.59)**	**17,134 (11,909-24,519)**	**459.87 (319.62-658.08)**	**1.121(0.867-1.376)**	**<0.001**
**Republic of Moldova**	**123 (78-190)**	**24.12 (15.22-37.16)**	**92 (61-135)**	**34.53 (23.02-51.04)**	**1.167(1.111-1.222)**	**<0.001**
**Romania**	**606 (384-932)**	**20.91 (13.25-32.15)**	**457 (298-700)**	**30.3 (19.8-46.44)**	**1.253(1.066-1.441)**	**<0.001**
**Russian Federation**	**4,491 (2,932-6,696)**	**29.09 (18.99-43.37)**	**4,455 (2,970-6,614)**	**38.75 (25.84-57.53)**	**0.932(0.876-0.987)**	**<0.001**
**Rwanda**	**1,029 (700-1,524)**	**88.98 (60.56-131.79)**	**2,386 (1,616-3,532)**	**109.59 (74.2-162.2)**	**0.694(0.63-0.757)**	**<0.001**
**Saint Kitts and Nevis**	**13 (9-18)**	**203.95 (137.6-291.4)**	**15 (10-22)**	**247.58 (169.68-360.8)**	**0.626(0.601-0.652)**	**<0.001**
**Saint Lucia**	**39 (26-55)**	**168.85 (112.22-241.95)**	**36 (25-52)**	**206.08 (139.93-294.53)**	**0.649(0.572-0.726)**	**<0.001**
**Saint Vincent and the Grenadines**	**29 (19-42)**	**156.98 (103.33-225.69)**	**29 (19-41)**	**225.02 (150.13-320.11)**	**1.183(1.108-1.258)**	**<0.001**
**Samoa**	**79 (54-116)**	**289.7**	**117 (80-170)**	**387.23 (265.05-**	**0.944(0.865-**	**<0.001**

		(197.09-425.75)		561.25)	1.023)	
San Marino	11 (8-16)	407.66 (280.39-580.72)	14 (9-19)	515.51 (353.78-719.6)	0.753(0.712-0.793)	<0.001
Sao Tome and Principe	16 (11-24)	79.64 (53.22-117.62)	41 (28-60)	116.98 (80.05-169.79)	1.255(1.171-1.34)	<0.001
Saudi Arabia	6,703 (4,525-9,629)	288.35 (194.66-414.22)	13,110 (8,947-18,716)	357.57 (244.02-510.48)	0.716(0.644-0.788)	<0.001
Senegal	1,051 (717-1,552)	83.54 (57.03-123.4)	2,639 (1,780-3,828)	104.68 (70.6-151.82)	0.74(0.66-0.82)	<0.001
Serbia	227 (144-350)	21.24 (13.47-32.71)	215 (142-313)	28.18 (18.54-41.01)	0.922(0.809-1.034)	<0.001
Seychelles	42 (30-60)	379.06 (265.91-541.5)	58 (40-81)	542.47 (378.22-763.51)	1.178(1.097-1.26)	<0.001
Sierra Leone	378 (252-532)	60.21 (40.09-84.66)	1563 (1,057-2,279)	105.27 (71.23-153.52)	1.836(1.772-1.901)	<0.001
Singapore	1,337 (912-1,911)	338.46 (230.76-483.92)	2,000 (1,378-2,920)	561.31 (386.87-819.66)	1.633(1.406-1.861)	<0.001
Slovakia	141 (90-216)	22.56 (14.36-34.53)	122 (80-183)	29.95 (19.75-45.02)	0.921(0.839-1.003)	<0.001
Slovenia	50 (32-76)	22.77 (14.65-34.88)	47 (30-71)	32.92 (21.18-49.35)	1.19(1.071-1.31)	<0.001
Solomon Islands	103 (70-148)	182.73 (124.55-263.94)	289 (197-410)	283.77 (193.21-402.1)	1.4(1.346-1.455)	<0.001
Somalia	875 (583-1,297)	74.62 (49.7-110.64)	2,954 (2,004-4,338)	84.88 (57.59-124.64)	0.408(0.365-0.451)	<0.001
South Africa	8,140 (5,606-11,732)	134.7	12,432 (8,534-17,861)	176.07 (120.86-	0.901(0.819-	<0.001

		(92.77-194.14)		252.96)	0.983)	
South Sudan	751 (509-1,092)	79.41 (53.77-115.45)	1,509 (1,012-2,220)	90.71 (60.84-133.48)	0.459(0.347-0.572)	<0.001
Spain	19,092 (13,142-26,830)	401.38 (276.3-564.08)	17,716 (12,173-25,334)	519.73 (357.14-743.23)	0.866(0.806-0.926)	<0.001
Sri Lanka	7,394 (5,158-10,552)	287.39 (200.47-410.14)	12,760 (8,958-17,768)	488.96 (343.25-680.86)	1.774(1.635-1.914)	<0.001
Sudan	4,977 (3,355-7,123)	154.56 (104.19-221.2)	19,096 (13,107-27,030)	274.77 (188.59-388.93)	1.885(1.789-1.982)	<0.001
Suriname	101 (68-145)	170.81 (115.26-244.92)	162 (109-231)	234.14 (157.67-333.26)	1.036(0.979-1.093)	<0.001
Sweden	2,417 (1,696-3,434)	299.18 (209.87-425.04)	3,389 (2,357-4,829)	396.68 (275.9-565.13)	0.907(0.788-1.027)	<0.001
Switzerland	2,396 (1,652-3,398)	371.39 (256.1-526.74)	2,967 (2,068-4,259)	452.3 (315.19-649.23)	0.642(0.578-0.706)	<0.001
Syrian Arab Republic	5,151 (3,437-7,354)	242.47 (161.81-346.19)	7,230 (5,021-10,435)	300.21 (208.47-433.28)	0.738(0.519-0.959)	<0.001
Taiwan (Province of China)	6,296 (4,192-9,037)	226.13 (150.55-324.61)	5,476 (3,911-7,419)	325.06 (232.15-440.41)	1.162(1.068-1.255)	<0.001
Tajikistan	332 (222-493)	40.02 (26.8-59.47)	692 (455-1,029)	51.22 (33.71-76.14)	0.807(0.717-0.896)	<0.001
Thailand	23,491 (16,327-33,332)	266.2 (185.02-377.71)	28,987 (20,460-40,777)	497.95 (351.47-700.48)	2.044(1.929-2.158)	<0.001
Timor-Leste	199 (137-283)	182.23 (125.7-259.62)	735 (520-1,064)	316.88 (224.08-458.4)	1.829(1.738-1.92)	<0.001
Togo	398 (266-583)	66.18	1,293 (879-1,908)	101.31 (68.89-	1.424(1.301-	<0.001

		(44.2-97.04)		149.53)	1.546)	
**Tokelau**	**1 (0-1)**	**248.94 (169.58-359.67)**	**1 (1-1)**	**415.53 (285.28-598.01)**	**1.664(1.154-2.175)**	**<0.001**
**Tonga**	**53 (36-76)**	**328.49 (221.84-471.29)**	**66 (45-96)**	**437.29 (296.25-634.53)**	**0.917(0.887-0.947)**	**<0.001**
**Trinidad and Tobago**	**302 (199-437)**	**177.99 (117.03-257.55)**	**343 (227-495)**	**254.69 (168.84-367.84)**	**1.175(1.077-1.273)**	**<0.001**
**Tunisia**	**2,905 (1,960-4,311)**	**220.83 (148.99-327.67)**	**3,754 (2,581-5,373)**	**306.94 (211.01-439.35)**	**1.108(1.04-1.177)**	**<0.001**
**Turkey**	**19,083 (13,066-27,369)**	**212.32 (145.37-304.51)**	**26,153 (17,929-37,195)**	**278.75 (191.1-396.45)**	**0.883(0.709-1.059)**	**<0.001**
**Turkmenistan**	**289 (190-426)**	**51.03 (33.56-75.12)**	**468 (318-682)**	**73.42 (49.88-107.03)**	**1.197(1.146-1.248)**	**<0.001**
**Tuvalu**	**3 (2-4)**	**218.17 (149.35-312.4)**	**6 (4-8)**	**356.65 (246.11-507.76)**	**1.582(1.532-1.633)**	**<0.001**
**Uganda**	**2,366 (1,592-3,406)**	**81.49 (54.82-117.33)**	**7,828 (5,390-11,344)**	**103.64 (71.36-150.19)**	**0.805(0.735-0.876)**	**<0.001**
**Ukraine**	**1,460 (955-2,229)**	**27.28 (17.84-41.64)**	**1,056 (678-1,592)**	**33.04 (21.22-49.81)**	**0.675(0.626-0.725)**	**<0.001**
**United Arab Emirates**	**504 (343-729)**	**274.82 (187.3-397.51)**	**1,988 (1,344-2,876)**	**369.56 (249.83-534.54)**	**0.931(0.7-1.163)**	**<0.001**
**United Kingdom**	**24,318 (17,020-34,003)**	**417.68 (292.32-584.02)**	**33,698 (23,658-47,283)**	**566.29 (397.57-794.6)**	**0.996(0.947-1.046)**	**<0.001**
**United Republic of Tanzania**	**4,207 (2,793-6,154)**	**95.17 (63.18-139.22)**	**11,474 (7,737-16,453)**	**115.88 (78.14-166.16)**	**0.645(0.605-0.686)**	**<0.001**
**United States of**	**126,225 (88,392-176,969)**	**467.03**	**192,835 (141,430-**	**607.94 (445.88-**	**0.805(0.608-**	**<0.001**

America		(327.05-654.78)	256,567)	808.87)	1.002)	
United States Virgin Islands	38 (25-56)	269.62 (180.74-396.27)	23 (15-33)	332.31 (221.86-481.62)	0.685(0.634-0.735)	<0.001
Uruguay	701 (487-1,004)	184.74 (128.3-264.7)	1,034 (721-1,496)	291.9 (203.51-422.66)	1.502(1.437-1.566)	<0.001
Uzbekistan	1,674 (1,113-2,458)	52.53 (34.93-77.13)	2,785 (1,865-3,937)	71.28 (47.73-100.79)	1.005(0.947-1.063)	<0.001
Vanuatu	48 (33-70)	208.27 (143.27-301.31)	140 (95-204)	305.36 (207.44-444.64)	1.208(1.163-1.253)	<0.001
Venezuela (Bolivarian Republic of)	9,283 (6,207-13,742)	311.06 (207.98-460.47)	12,062 (8,271-17,473)	401.76 (275.47-581.98)	0.836(0.682-0.99)	<0.001
Viet Nam	18,904 (12,987-26,610)	174.58 (119.95-245.76)	36,698 (25,380-51,715)	357.39 (247.17-503.64)	2.367(2.288-2.446)	<0.001
Yemen	3,433 (2,334-5,025)	169.23 (115.02-247.71)	11,058 (7,404-16,094)	204.58 (136.98-297.77)	0.627(0.586-0.669)	<0.001
Zambia	1,587 (1,078-2,313)	110.93 (75.33-161.63)	4,532 (3,017-6,520)	134.57 (89.59-193.62)	0.642(0.561-0.724)	<0.001
Zimbabwe	2,191 (1,453-3,185)	119.6 (79.31-173.85)	3,277 (2,244-4704)	126.14 (86.38-181.09)	0.212(0.091-0.333)	0.001

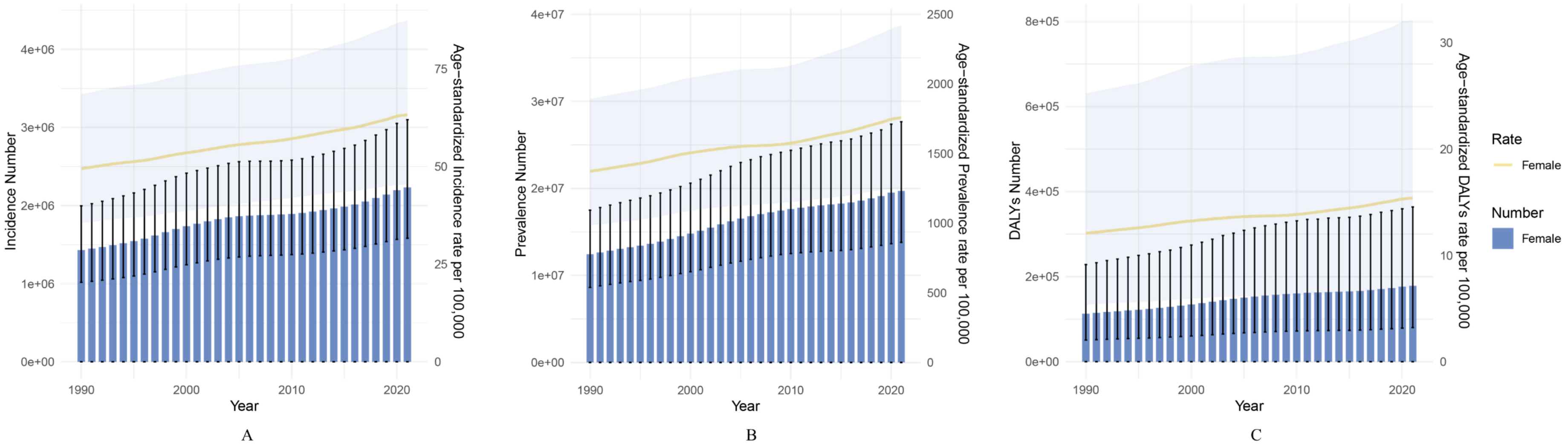

Figure S1. Global trends in the burden of PCOS among females aged 10–24 from 1990 to 2021 (with ten-year intervals).A. Changes in the number of PCOS cases and the age-standardized incidence rate.B. Changes in the number of prevalent PCOS cases and the age-standardized prevalence rate.C. Changes in the number of DALYs caused by PCOS and the age-standardized DALYs rate.The shaded areas represent the 95% confidence intervals.

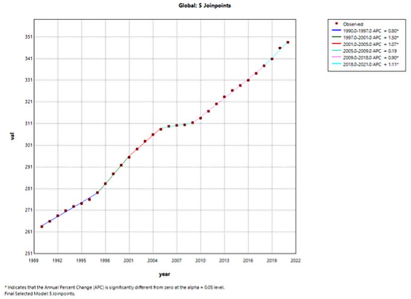

A

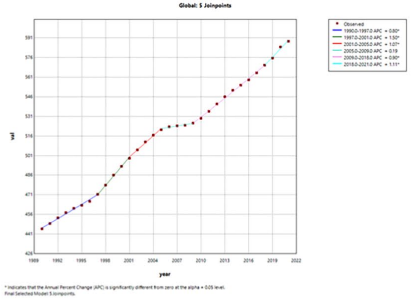

B

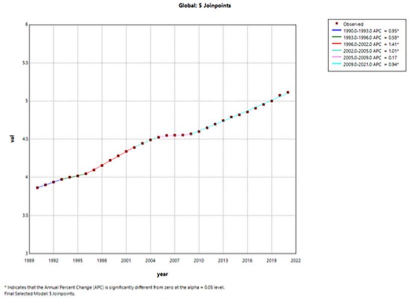

C

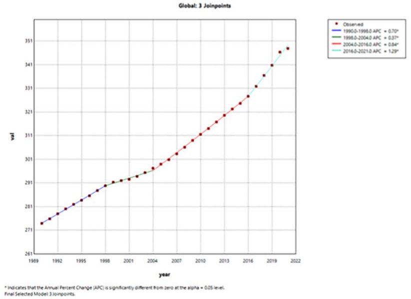

D

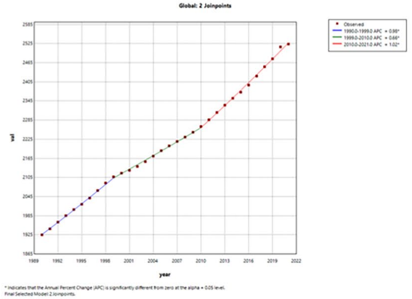

E

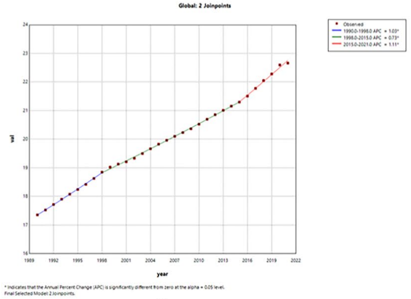

F

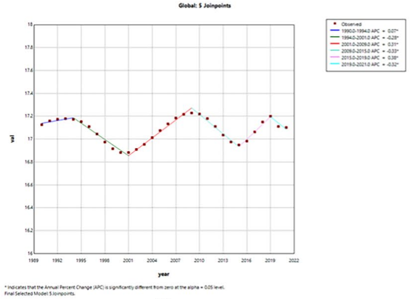

G

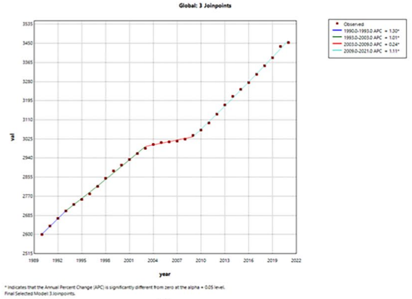

H

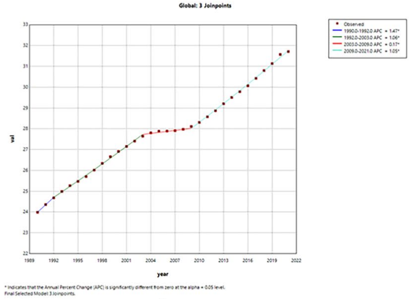

I

Figure S2. Joinpoint regression analysis of PCOS among females aged 10-24 worldwide from 1990 to 2021. A. Trends in the incidence rate of PCOS among females aged 10-14; B. Trends in the prevalence rate of PCOS among females aged 10-14; C. Trends in the DALYs rate of PCOS among

females aged 10-14; D. Trends in the incidence rate of PCOS among females aged 15-19; E. Trends in the prevalence rate of PCOS among females aged 15-19; F. Trends in the DALYs rate of PCOS among females aged 15-19; G. Trends in the incidence rate of PCOS among females aged 20-24; H. Trends in the prevalence rate of PCOS among females aged 20-24; I. Trends in the DALYs rate of PCOS among females aged 20-24.

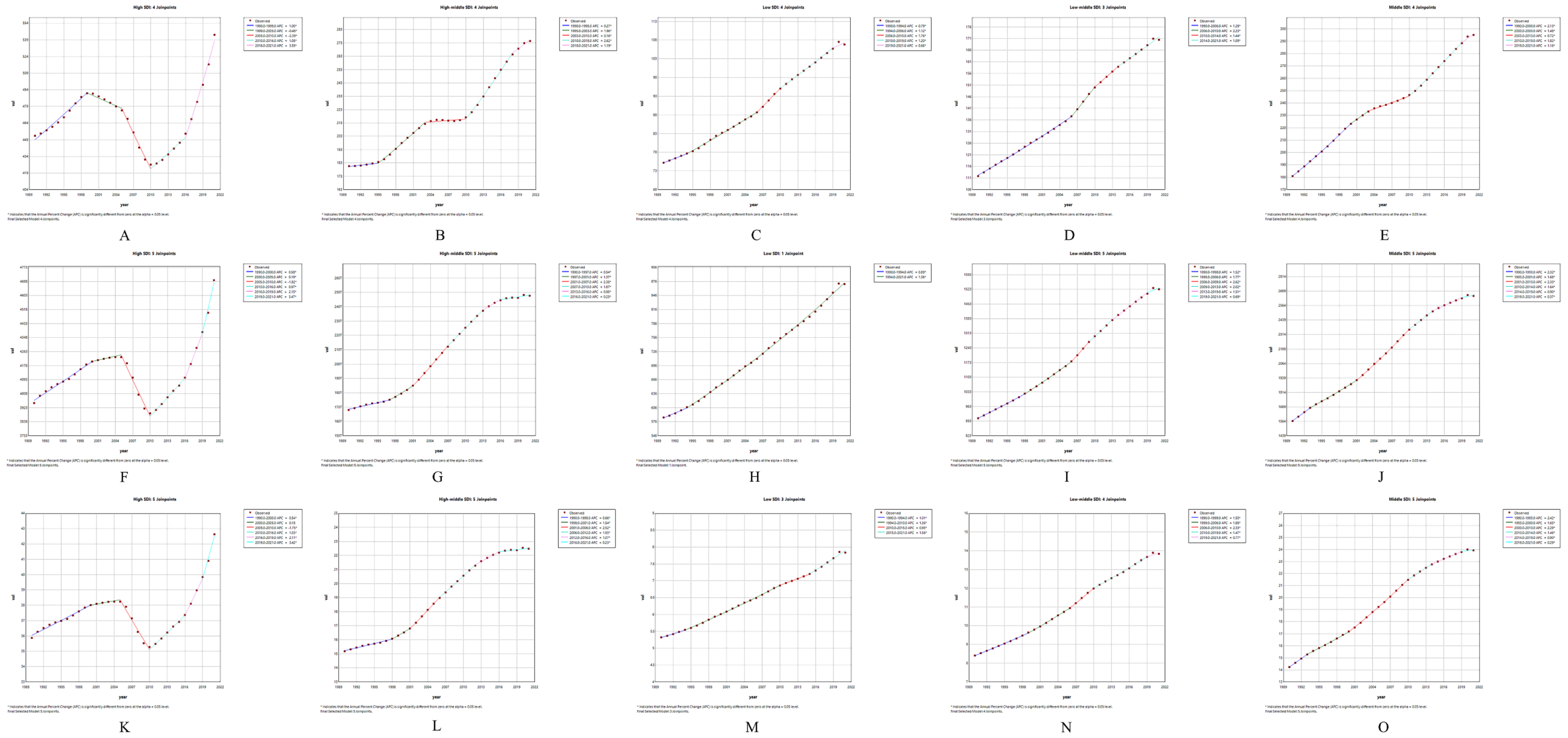

Figure S3. Joinpoint regression analysis of PCOS among females aged 10–24 globally from 1990 to 2021, stratified by Socio-demographic Index (SDI) quintiles. A. Trends in PCOS incidence rates for females aged 10–24 in high SDI regions; B. Trends in PCOS incidence rates for females aged 10–24 in high-middle SDI regions; C. Trends in PCOS incidence rates for females aged 10–24 in middle SDI regions; D. Trends in PCOS incidence rates for females aged 10–24 in low-middle SDI regions; E. Trends in PCOS incidence rates for females aged 10–24 in low SDI regions; F. Trends in

PCOS prevalence rates for females aged 10–24 in high SDI regions; G. Trends in PCOS prevalence rates for females aged 10–24 in high-middle SDI regions; H. Trends in PCOS prevalence rates for females aged 10–24 in middle SDI regions; I. Trends in PCOS prevalence rates for females aged 10–24 in low-middle SDI regions; J. Trends in PCOS prevalence rates for females aged 10–24 in low SDI regions; K. Trends in PCOS DALYs for females aged 10–24 in high SDI regions; L. Trends in PCOS DALYs for females aged 10–24 in high-middle SDI regions; M. Trends in PCOS DALYs for females aged 10–24 in middle SDI regions; N. Trends in PCOS DALYs for females aged 10–24 in low-middle SDI regions; O. Trends in PCOS DALYs for females aged 10–24 in low SDI regions.

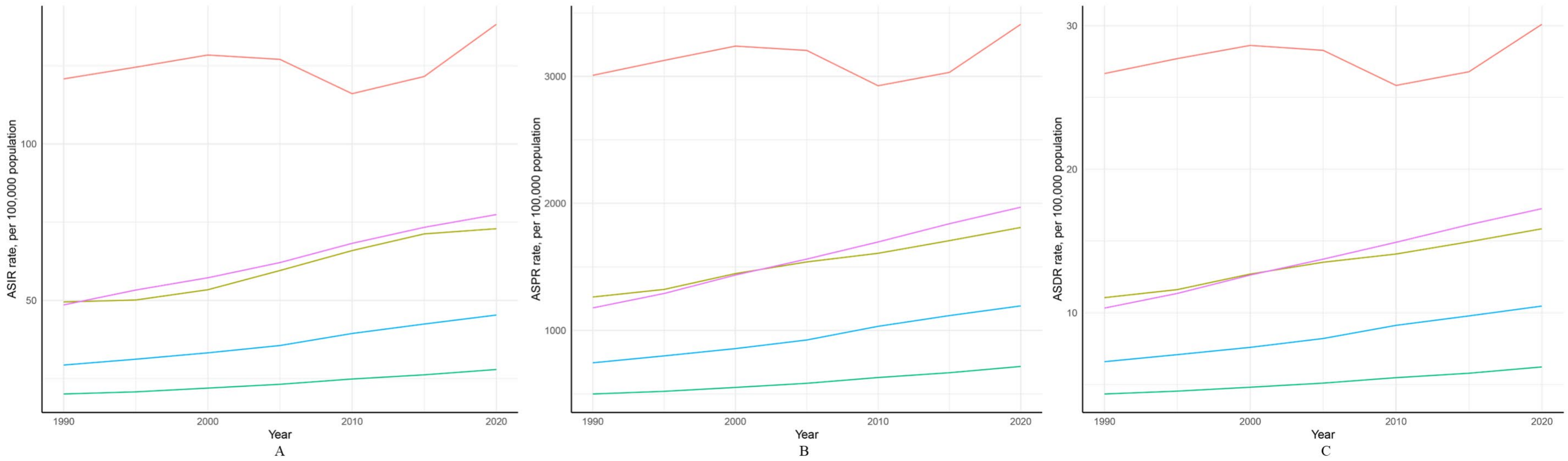

Figure S4. Global trends of PCOS among females aged 10–24 from 1990 to 2021, analyzed by SDI quintiles. A. Age-standardized incidence rates stratified by SDI quintiles; B. Age-standardized prevalence rates stratified by SDI quintiles; C. Age-standardized DALY rates stratified by SDI quintiles.

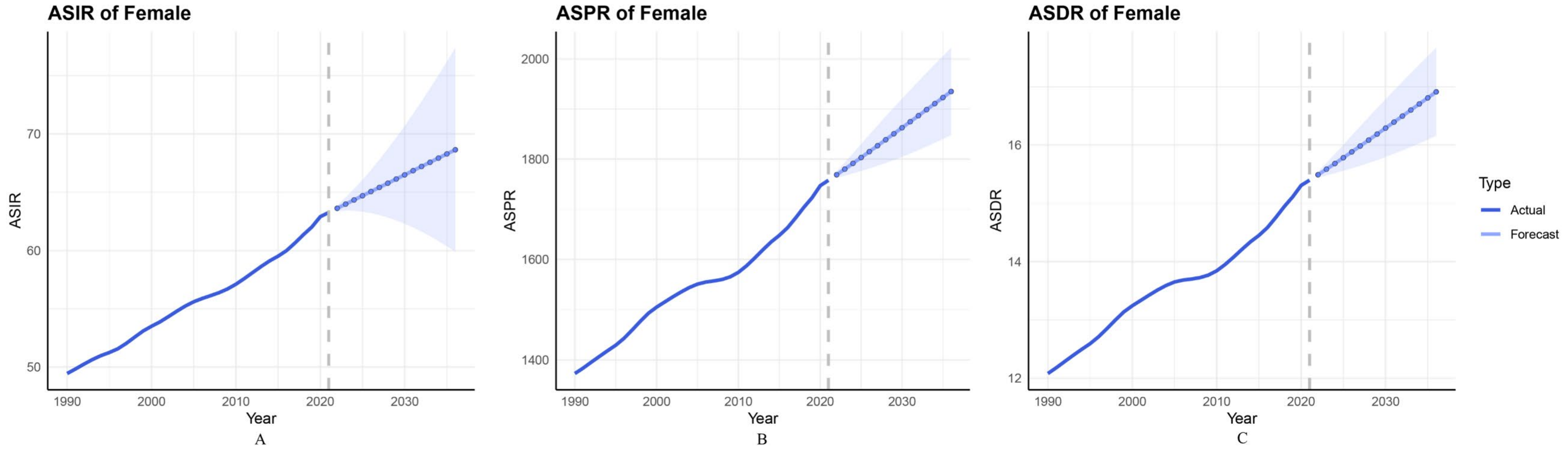

Figure S5. Trends and projections of age-standardized incidence rate, prevalence rate, and DALY rate of PCOS among females aged 10-24 from 1990 to 2036. A. Actual and projected age-standardized incidence rate of PCOS; B. Actual and projected age-standardized prevalence rate of PCOS; C. Actual and projected age-standardized DALY rate of PCOS. Solid lines represent actual data from 1990 to 2021, while dashed lines and markers represent projections for 2022 to 2036; error bars indicate the uncertainty range of the projections.

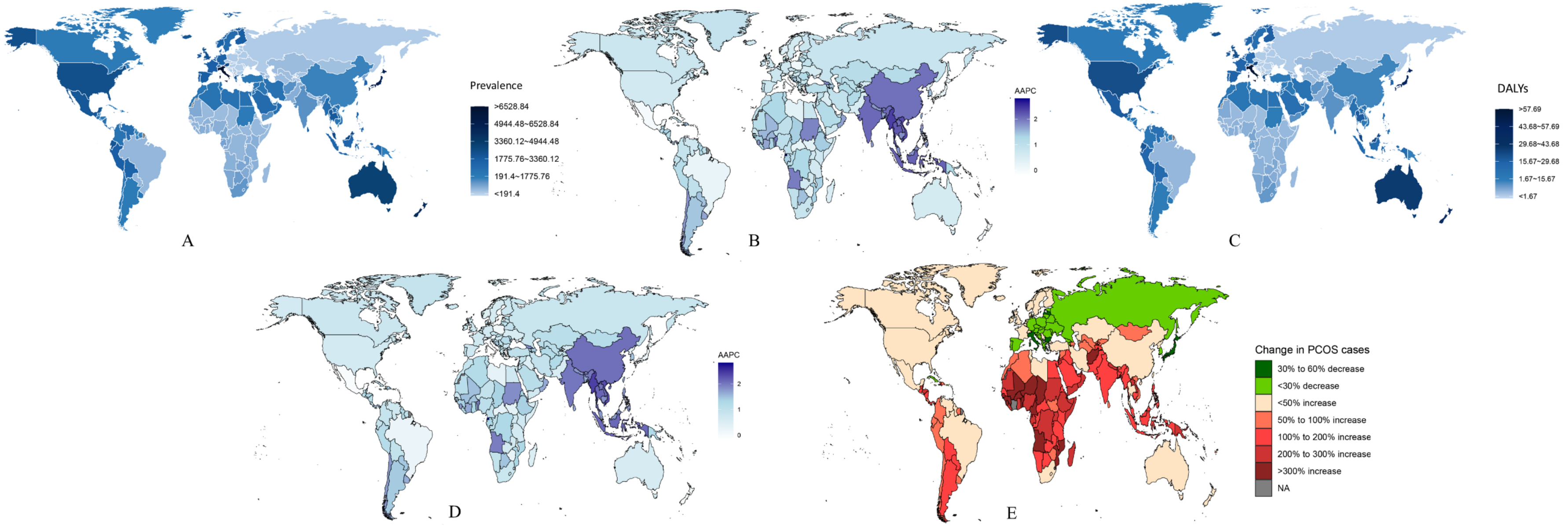

Figure S6. National burden of PCOS.A. Estimated prevalence rates in 2021.B. AAPC in prevalence rates by country. C. Estimated DALYs rates in 2021. D. AAPC in DALYs rates by country. E. Trends in DALYs rates from 1990 to 2019.
